# Supplementary material for: Efficacy and moderators of efficacy of trauma-focused cognitive behavioural therapies in children and adolescents: protocol for an individual participant data meta-analysis from randomised trials
Source: BMJ Open. 2021 Feb 24;11(2):e047212. doi: 10.1136/bmjopen-2020-047212 (PMC7908282; doi:10.1136/bmjopen-2020-047212)
Supplement: Supplementary data [file bmjopen-2020-047212supp001.pdf]

Search strategy de Haan et al.

Database	Number of hits
Medline	1913
Embase	2000
CINAHL	2506
Cochrane	2413
PsycINFO	2105
Total	10937
Total deduplicated	7091

Searches run 12th November 2019

Limited by publication date 2018 to date

Medline

Database: Ovid MEDLINE(R) and Epub Ahead of Print, In-Process & Other Non-Indexed Citations, Daily and Versions(R) <1946 to November 08, 2019>

Search Strategy:

----

1 stress disorders, traumatic/ or combat disorders/ or psychological trauma/ or stress disorders, post-traumatic/ or stress disorders, traumatic, acute/ or stress, psychological/ (147421)

2 (railway spine or (rape adj2 trauma*) or reexperienc* or re experienc* or torture syndrome or traumatic neuros* or traumatic stress).ti,ab. (15096)

3 (trauma* and (avoidance or grief or horror or death* or nightmare* or night mare* or emotion*)).ti,ab. (35178)

4 (posttraumatic* or post traumatic* or stress disorder* or acute stress or ptsd or asd or desnos or (combat neuros* or combat syndrome or concentration camp syndrome or extreme stress or flashback* or flash back* or hypervigilan* or hypervigilen* or psych* stress or psych* trauma* or psycho?trauma* or psychotrauma*) or (posttrauma* or traumagenic* or traumatic stress*)).ti,ab. (106800)

5 or/1-4 (245567)

6 psychotherapy/ (53279)

7 (((psycholog* or psycho social* or psychosocial*) adj3 (intervention* or program* or therap* or treat*)) or psychotherap* or psycho therap* or talk* therap* or therapeutic technique* or therapist* or third wave or time limited).ti,ab,sh. (144180)

8 exp behavior therapy/ (72171)

9 (((behaviour* or behavior*) adj2 cognitiv*) or cbt or ccbt or ((behav* or cognitive*) adj3 (intervention* or manag* or program* or restructure* or therap* or treat*)) or (stress inoculation adj2 (intervention* or program* or therap* or train* or treat*)) or (behav* adj2 activat*) or ((trauma adj (based or focused or led)) or exposure based or prolonged exposure)).ti,ab. (115271)

10 emotions/ (63893)

11 (((compassion or emotion* or emotive*) adj (based or focused or led)) or emotional processing or ((compassion or emotion* or emotive*) adj3 (coach* or intervention* orprogram* or therap* or treat*))).ti,ab. (7907)

12 implosive therapy/ or narrative therapy/ or virtual reality exposure therapy/ (1886)

13 (((augmented or virtual) adj2 reality) or (virtual adj (environment or restorative)) or ((exposure or implosive or virtual reality) adj2 (intervention* or program* or therap* or train*))).ti,ab. (16289)

14 ((imagery adj2 (rehears* or re hears*)) or (((lower* or reduc*) adj3 (bad dream* or nightmare*)) and (intervention* or program* or therap* or treat*)) or ((intervention* or program* or therap* or treat*) adj3 nightmare*)).mp. or ((presleep or presleep) adj2 imagery).ti,ab. (267)

15 (mindfulness or ((exposure or narrative) adj therapy)).sh. (2743)

16 (kidnet or mindful* or narrative therap*).ti,ab. (8835)

17 debrief*.ti,ab. (3803)

18 eye movement desensitization reprocessing/ (205)

19 (emdr or (eye movement adj2 desensiti*)).ti,ab. (671)

20 psychotherapy, psychodynamic/ (529)

21 (psychodynamic or (dynamic adj (psychotherapy* or therap*)) or incident reduction or ((brain or transcranial) adj2 stimulat*) or rtms).ti,ab. (36027)

22 (psychoanal* or psychosomatic*).ti,ab. (26156)

23 exp counseling/ (42669)

24 counsel*.ti,ab. (103170)

25 (hg therap* or human givens).ti,ab. (14)

26 couples therapy/ or family therapy/ or marital therapy/ or exp parents/ed (22720)

27 (((con?joint or couple* or family or families or husband* or marriage* or marital* or partner* or relations* or spous* or wife or wives* or (child* adj5 parent*)) adj6 (counsel* or intervention* or program* or support* or therap* or treat*)) or ((couples* or family* or relations*) adj (based or focused or led)) or ecological therap* or expressed emotion or family dynamics or family relationships).tw. (171018)

28 ((child* adj2 family traumatic stress intervention) or cftsi).ti,ab. (2)

29 psychoeducation.sh. or (psychoed* or psycho ed*).ti,ab. (5975)

30 community networks/ or friends/ or exp peer group/ or self care/ or self-help groups/ or social networking/ or social support/ (133084)

31 ((self adj (administer* or assess* or attribut* or care or change or directed or efficacy or help* or guide* or instruct* or manag* or medicat* or monitor* or regulat* or reinforc* or re inforc* or support* or technique* or therap* or train* or treat*)) or selfadminister* or selfassess* or selfattribut* or selfcare or selfchange or selfdirected or selfefficacy or selfhelp* or selfguide* or selfinstruct* or selfmanag* or selfmedicat* or selfmonitor* or selfregulat* or selfreinforc* or self re inforc* or selfsupport* or selftechnique* or selftherap* or selftrain* or selftreat* or (wellness adj (therap* or train* or treat*))).ti,ab,sh. (139336)

32 (befriend* or be*1 friend* or buddy or buddies or ((community or lay or paid or support) adj (person or worker*))).ti,ab. (3080)

33 (((consumer* or famil* or friend* or lay or mutual* or peer* or social* or spous* or voluntary or volunteer*) adj3 (assist* or advice* or advis* or counsel* or educat* or forum* or help* or mentor* or network* or support* or visit*)) or ((consumer* or famil* or peer* or self help or social* or support* or voluntary or volunteer*) adj2 group*) or ((consumer* or famil* or friend* or lay or mutual* or peer* or self

help or social* or spous* or support* or voluntary or volunteer*) adj3 (intervention* or program* or rehab* or therap* or service* or skill* or treat*)) or (((consumer* or famil* or friend* or lay* or peer* or spous* or user* or support* or voluntary or volunteer*) adj (based or counsel* or deliver* or interact* or led or mediat* or operated or provides or provider* or run*)) or ((consumer* or famil* or friend* or lay* or peer* or relation* or spous* or support*) adj3 trust*) or voluntary work*)).ti,ab. (314043)

34 (((lay or peer*) adj3 (advis* or consultant or educator* or expert* or facilitator* or instructor* or leader* or mentor* or person* or tutor* or worker*)) or expert patient* or mutual aid).ti,ab. (6617)

35 (peer* adj3 (assist* or counsel* or educat* or program* or rehab* or service* or supervis*)).ti,ab. (5735)

36 ((psychoeducat* or psycho educat*) adj3 (group or network* or service*)).ti,ab. (774)

37 ((psychosocial or social) adj work*).ti,ab. (15927)

38 ((ptsd or posttrauma* or post trauma* or trauma*) adj2 support*).ti,ab. (1565)

39 recovery support.ti,ab. (173)

40 assisted living facilities/ or emergency shelter/ or group homes/ or halfway houses/ or housing/ or independent living/ or residential facilities/ (30265)

41 ((resident* or hous* or accommod* or commun* or comu* or home*) adj5 (support* or support* or shelter* or outreach* or visit* or appointment*)).ti,ab. (50036)

42 (residential treatm* or residential facility* or supported hous* or public hous*).ti,ab. (3249)

43 (accomod* or assertive community treatment* or home* or housing* or outreach* or residential*).ti,ab. (525321)

44 placement.ti,ab. (122669)

45 or/6-44 (1653237)

46 meta analysis.sh,pt. or "meta-analysis as topic"/ or "review literature as topic"/ (129841)

47 (exp databases, bibliographic/ or (((electronic or computer* or online) adj database*) or bids or cochrane or embase or index medicus or isi citation or medline or psyclit or psychlit or scisearch or science citation or (web adj2 science)).ti,ab.) and (review*.ti,ab,sh,pt. or systematic*.ti,ab.) (163499)

48 ((analy* or assessment* or evidence* or methodol* or quantativ* or systematic*) adj2 (overview* or review*)).tw. or ((analy* or assessment* or evidence* or methodol* or quantativ* or systematic*).ti. and review*.ti,pt.) or (systematic* adj2 search*).ti,ab. (329117)

49 (metaanal* or meta anal* or (research adj (review* or integration))).ti,ab. or reference list*.ab. or bibliograph*.ab. or published studies.ab. or relevant journals.ab. or selection criteria.ab. or (data adj (extraction or synthesis)).ab. or (handsearch* or ((hand or manual) adj search*)).ti,ab. or (mantel haenszel or peto or dersimonian or der simonian).ti,ab. or (fixed effect* or random effect*).ti,ab. or ((pool* or combined or combining) adj2 (data or trials or studies or results)).ti,ab. (300023)

50 or/46-49 (562928)

51 exp clinical trial/ or exp "clinical trials as topic"/ or cross-over studies/ or double-blind method/ or placebos/ or random allocation/ or single-blind method/ (1194973)

52 ((clinical adj2 trial*) or (crossover or cross over)).ti,ab. (429760)

53 (((single* or doubl* or trebl* or tripl*) adj2 blind*) or mask* or dummy or doubleblind* or singleblind* or trebleblind* or tripleblind*).ti,ab. (240549)

54 (placebo* or random*).ti,ab. (1162532)

55 or/51-54 (2043304)

56 5 and 45 and (50 or 55) (12008)

57 animals/ not human*.mp. (4342443)

58 56 not 57 (11757)

59 limit 58 to yr="2018 -Current" (1913)

****

Embase

Database: Embase <1974 to 2019 November 08>

Search Strategy:

----

1 *acute stress/ or *behavioural stress/ or *emotional stress/ or *critical incident stress/ or *mental stress/ or *posttraumatic stress disorder/ or *psychotrauma/ (69082)

2 (trauma* and (avoidance or grief or horror or death* or nightmare* or night mare* or emotion*)).ti,ab. (50168)

3 (railway spine or (rape adj2 trauma*) or reexperienc* or re experienc* or torture syndrome or traumatic neuros* or traumatic stress).ti,ab. (20306)

4 (posttraumatic* or post traumatic* or stress disorder* or acute stress or ptsd or asd or desnos or (combat neuros* or combat syndrome or concentration camp syndrome or extreme stress or flashback* or flash back* or hypervigilan* or hypervigilen* or psych* stress or psych* trauma* or psycho?trauma* or psychotrauma*) or (posttrauma* or traumagenic* or traumatic stress*)).ti,ab. (139168)

5 or/1-4 (212899)

6 *psychotherapy/ (38266)

7 exp *behavior therapy/ or exp *cognitive therapy/ (29422)

8 (((psycholog* or psycho social* or psychosocial*) adj3 (intervention* or program* or therap* or treat*)) or psychotherap* or psycho therap* or talk* therap* or therapeutic technique* or therapist* or third wave or time limited).ti,ab,sh. (246754)

9 (((behaviour* or behavior*) adj2 cognitiv*) or cbt or ccbt or ((behav* or cognitive*) adj3 (intervention* or manag* or program* or restructure* or therap* or treat*)) or (stress inoculation adj2 (intervention* or program* or therap* or train* or treat*)) or (behav* adj2 activat*) or ((trauma adj (based or focused or led)) or exposure based or prolonged exposure)).ti,ab. (158532)

10 *emotion/ (28919)

11 (((compassion or emotion* or emotive*) adj (based or focused or led)) or emotional processing or ((compassion or emotion* or emotive*) adj3 (coach* or intervention* orprogram* or therap* or treat*))).ti,ab. (10888)

12 exposure therapy/ or narrative therapy/ or virtual reality exposure therapy/ (1602)

13 (((augmented or virtual) adj2 reality) or (virtual adj (environment or restorative)) or ((exposure or implosive or virtual reality) adj2 (intervention* or program* or therap* or train*))).ti,ab. (21776)

14 ((imagery adj2 (rehears* or re hears*)) or (((lower* or reduc*) adj3 (bad dream* or nightmare*)) and (intervention* or program* or therap* or treat*)) or ((intervention* or program* or therap* or treat*) adj3 nightmare*)).mp. or ((presleep or presleep) adj2 imagery).ti,ab. (454)

15 (mindfulness or ((exposure or narrative) adj therapy)).sh. (23980)

16 (kidnet or mindful* or narrative therap*).ti,ab. (12154)

17 debrief*.ti,ab. (6509)

18 (emdr or (eye movement adj2 desensiti*)).ti,ab. (925)

19 psychodynamic psychotherapy/ (1214)

20 (psychodynamic or (dynamic adj (psychotherapy* or therap*)) or incident reduction or ((brain or transcranial) adj2 stimulat*) or rtms).ti,ab. (53209)

21 (psychoanal* or psychosomatic*).ti,ab. (35159)

22 exp counseling/ (159666)

23 counsel*.ti,ab. (148163)

24 (hg therap* or human givens).ti,ab. (21)

25 couple therapy/ or family therapy/ or marital therapy/ or exp parent/ (246712)

26 (((con?joint or couple* or family or families or husband* or marriage* or marital* or partner* or relations* or spous* or wife or wives* or (child* adj5 parent*)) adj6 (counsel* or intervention* or program* or support* or therap* or treat*)) or ((couples* or family* or relations*) adj (based or focused or led)) or ecological therap* or expressed emotion or family dynamics or family relationships).tw. (226599)

27 ((child* adj2 family traumatic stress intervention) or cftsi).ti,ab. (6)

28 psychoeducation.sh. or (psychoed* or psycho ed*).ti,ab. (12914)

29 friendship/ or peer counseling/ or peer group/ or self help/ or self care/ or social network/ or social support/ or support group/ (193307)

30 ((self adj (administer* or assess* or attribut* or care or change or directed or efficacy or help* or guide* or instruct* or manag* or medicat* or monitor* or regulat* or reinforc* or re inforc* or support* or technique* or therap* or train* or treat*)) or selfadminister* or selfassess* or selfattribut* or selfcare or selfchange or selfdirected or selfefficacy or selfhelp* or selfguide* or selfinstruct* or selfmanag* or selfmedicat* or selfmonitor* or selfregulat* or selfreinforc* or self re inforc* or selfsupport* or selftechnique* or selftherap* or selftrain* or selftreat* or (wellness adj (therap* or train* or treat*))).ti,ab,sh. (188037)

31 (befriend* or be*1 friend* or buddy or buddies or ((community or lay or paid or support) adj (person or worker*))).ti,ab. (4130)

32 (((consumer* or famil* or friend* or lay or mutual* or peer* or social* or spous* or voluntary or volunteer*) adj3 (assist* or advice* or advis* or counsel* or educat* or forum* or help* or mentor* or network* or support* or visit*)) or ((consumer* or famil* or peer* or self help or social* or support* or voluntary or volunteer*) adj2 group*) or ((consumer* or famil* or friend* or lay or mutual* or peer* or self help or social* or spous* or support* or voluntary or volunteer*) adj3 (intervention* or program* or rehab* or therap* or service* or skill* or treat*)) or (((consumer* or famil* or friend* or lay* or peer* or spous* or user* or support* or voluntary or volunteer*) adj (based or counsel* or deliver* or interact* or led or mediat* or operated or provides or provider* or run*)) or ((consumer* or famil* or friend* or lay* or peer* or relation* or spous* or support*) adj3 trust*) or voluntary work*)).ti,ab. (410161)

33 (((lay or peer*) adj3 (advis* or consultant or educator* or expert* or facilitator* or instructor* or leader* or mentor* or person* or tutor* or worker*)) or expert patient* or mutual aid).ti,ab. (8679)

34 (peer* adj3 (assist* or counsel* or educat* or program* or rehab* or service* or supervis*)).ti,ab. (7548)

35 ((psychoeducat* or psycho educat*) adj3 (group or network* or service*)).ti,ab. (1226)

36 ((psychosocial or social) adj work*).ti,ab. (22915)

37 ((ptsd or posttrauma* or post trauma* or trauma*) adj2 support*).ti,ab. (1890)

38 recovery support.ti,ab. (216)

39 assisted living facility/ or emergency shelter/ or halfway house/ or housing/ or independent living/ or residential home/ or residential home/ (36467)

40 ((resident* or hous* or accommod* or commun* or comu* or home*) adj5 (support* or support* or shelter* or outreach* or visit* or appointment*)).ti,ab. (66837)

41 (residential treatm* or residential facility* or supported hous* or public hous*).ti,ab. (3836)

42 (accomod* or assertive community treatment* or home* or housing* or outreach* or residential*).ti,ab. (693104)

43 placement.ti,ab. (174618)

44 or/6-43 (2352266)

45 meta analysis/ or "meta analysis (topic)"/ or systematic review/ (349285)

46 (exp bibliographic database/ or (((electronic or computer* or online) adj database*) or bids or cochrane or embase or index medicus or isi citation or medline or psyclit or psychlit orscisearch or science citation or (web adj2 science)).ti,ab.) and (review*.ti,ab,sh,pt. or systematic*.ti,ab.) (223431)

47 ((analy* or assessment* or evidence* or methodol* or quantativ* or systematic*) adj2 (overview* or review*)).tw. or ((analy* or assessment* or evidence* or methodol* or quantativ* or systematic*).ti. and review*.ti,pt.) or (systematic* adj2 search*).ti,ab. (392909)

48 (metaanal* or meta anal* or (research adj (review* or integration))).ti,ab. or reference list*.ab. or bibliograph*.ab. or published studies.ab. or relevant journals.ab. or selection criteria.ab. or (data adj (extraction or synthesis)).ab. or (handsearch* or ((hand or manual) adj search*)).ti,ab. or (mantel haenszel or peto or dersimonian or der simonian).ti,ab. or (fixed effect* or random effect*).ti,ab. or ((pool* or combined or combining) adj2 (data or trials or studies or results)).ti,ab. (393628)

49 or/45-48 (775067)

50 exp "clinical trial (topic)"/ or exp clinical trial/ or crossover procedure/ or double blind procedure/ or placebo/ or randomization/ or random sample/ or single blind procedure/ (1962363)

51 (clinical adj2 trial*).ti,ab. (504366)

52 (crossover or cross over).ti,ab. (101345)

53 (((single* or doubl* or trebl* or tripl*) adj2 blind*) or mask* or dummy or doubleblind* or singleblind* or trebleblind* or tripleblind*).ti,ab. (323907)

54 (placebo* or random*).ti,ab. (1578946)

55 or/50-54 (2965134)

56 5 and 44 and (49 or 55) (10225)

57 animals/ not human*.mp. (857762)

58 56 not 57 (10209)

59 limit 58 to yr="2018 -Current" (2000)

****

PsycINFO

#	Query	Results
S64	s59 not s60 Limiters - Publication Year: 2018-2020	2,105
S63		277,708
S62		1,761,949
S61	s59 not s60	16,053
S60	animal not human	289,401
S59	S5 AND S48 AND S58	16,256
S58	S56 OR S57	765,475
S57	((clinical trials or placebo or random sampling)) OR (clinical adj2 trial*) OR (crossover or cross over) OR ((((single* or doubl* or trebl* or tripl*) n2 blind*) or mask* or dummy or doubleblind* or singleblind* or trebleblind* or tripleblind*)) OR (placebo* or random*) OR treatment outcome*	347,654
S56	S49 OR S50 OR S51 OR S52 OR S53 OR S54 OR S55	468,514
S55	(metaanal* or meta anal* or (research n1 (review* or integration))) OR (reference list* or bibliograph* or published studies or relevant journals or selection criteria) OR ((data n1 (extraction or synthesis))) OR ((handsearch* or ((hand or manual) n1 search*))) OR ((mantel haenszel or peto or dersimonian or der simonian)) OR ((fixed effect* or random effect*)) OR (((pool* or combined or combining) n2 (data or trials or studies or results)))	142,371
S54	(systematic* n2 search*)	6,258
S53	(analy* or assessment* or evidence* or methodol* or quantativ* or systematic*) AND review*	297,715
S52	((analy* or assessment* or evidence* or methodol* or quantativ* or systematic*) n2 (overview* or review*))	317,006
S51	(((electronic or computer* or online) n1 database*) or bids or cochrane or embase or index medicus or isi citation or medline or psyclit or psychlit or scisearch or science citation or (web n2 science)) AND (review* or systematic*)	53,766
S50	computer searching	1,605

S49	((literature review or meta analysis)) OR systematic review	138,174
S48	S6 OR S7 OR S8 OR S9 OR S10 OR S11 OR S12 OR S13 OR S14 OR S15 OR S16 OR S17 OR S18 OR S19 OR S20 OR S21 OR S22 OR S23 OR S24 OR S25 OR S26 OR S27 OR S28 OR S29 OR S30 OR S31 OR S32 OR S33 OR S34 OR S35 OR S36 OR S37 OR S38 OR S39 OR S40 OR S41 OR S42 OR S43 OR S44 OR S45 OR S46 OR S47	1,682,227
S47	TI (placement or student placement) OR AB placement	26,717
S46	TI ((accomod* or assertive community treatment* or home* or housing* or outreach* or residential*)) OR AB ((accomod* or assertive community treatment* or home* or housing* or outreach* or residential*))	190,252
S45	TI ((residential treatm* or residential facility* or supported hous* or public hous*)) OR AB ((residential treatm* or residential facility* or supported hous* or public hous*))	9,115
S44	TI (((resident* or hous* or accommod* or commun* or comu* or home*) n5 (support* or support* or shelter* or outreach* or visit* or appointment*))) OR AB (((resident* or hous* or accommod* or commun* or comu* or home*) n5 (support* or support* or shelter* or outreach* or visit* or appointment*)))	39,683
S43	TI (((resident* or hous* or accommod* or commun* or comu* or home*) adj5 (support* or support* or shelter* or outreach* or visit* or appointment*))) OR AB (((resident* or hous* or accommod* or commun* or comu* or home*) adj5 (support* or support* or shelter* or outreach* or visit* or appointment*)))	0
S42	(((((DE "Assisted Living") OR (DE "Shelters")) OR (DE "Group Homes")) OR (DE "Halfway Houses")) OR (DE "Housing")) OR (DE "Residential Care Institutions")	19,622
S41	TI recovery support OR AB recovery support	2,144
S40	TI (((ptsd or posttrauma* or post trauma* or trauma*) n2 support*)) OR AB (((ptsd or posttrauma* or post trauma* or trauma*) n2 support*))	1,570
S39	TI (((psychosocial or social) n1 work*)) OR AB (((psychosocial or social) n1 work*))	48,187
S38	TI (((psychoeducat* or psycho educat*) n3 (group or network* or service*))) OR AB (((psychoeducat* or psycho educat*) n3 (group or network* or service*)))	1,967
S37	TI ((peer* n3 (assist* or counsel* or educat* or program* or rehab* or service* or supervis*))) OR AB ((peer* n3 (assist* or counsel* or educat* or program* or rehab* or service* or supervis*)))	7,910
S36	TI ((((lay or peer*) n3 (advis* or consultant or educator* or expert* or facilitator* or instructor* or leader* or mentor* or person* or tutor* or	10,195

	worker*)) or expert patient* or mutual aid)) OR AB ((((lay or peer*) n3 (advis* or consultant or educator* or expert* or facilitator* or instructor* or leader* or mentor* or person* or tutor* or worker*)) or expert patient* or mutual aid))	
S35	TI ((((consumer* or famil* or friend* or lay or mutual* or peer* or social* or spous* or voluntary or volunteer*) n3 (assist* or advice* or advis* or counsel* or educat* or forum* or help* or mentor* or network* or support* or visit*)) or ((consumer* or famil* or peer* or self help or social* or support* or voluntary or volunteer*) n2 group*) or ((consumer* or famil* or friend* or lay or mutual* or peer* or self help or social* or spous* or support* or voluntary or volunteer*) n3 (intervention* or program* or rehab* or therap* or service* or skill* or treat*)) or (((consumer* or famil* or friend* or lay* or peer* or spous* or user* or support* or voluntary or volunteer*) n1 (based or counsel* or deliver* or interact* or led or mediat* or operated or provides or provider* or run*)) or ((consumer* or famil* or friend* or lay* or peer* or relation* or spous* or support*) n3 trust*) or voluntary work*))) OR AB ((((consumer* or famil* or friend* or lay or mutual* or peer* or social* or spous* or voluntary or volunteer*) n3 (assist* or advice* or advis* or counsel* or educat* or forum* or help* or mentor* or network* or support* or visit*)) or ((consumer* or famil* or peer* or self help or social* or support* or voluntary or volunteer*) n2 group*) or ((consumer* or famil* or friend* or lay or mutual* or peer* or self help or social* or spous* or support* or voluntary or volunteer*) n3 (intervention* or program* or rehab* or therap* or service* or skill* or treat*)) or (((consumer* or famil* or friend* or lay* or peer* or spous* or user* or support* or voluntary or volunteer*) n1 (based or counsel* or deliver* or interact* or led or mediat* or operated or provides or provider* or run*)) or ((consumer* or famil* or friend* or lay* or peer* or relation* or spous* or support*) n3 trust*) or voluntary work*)))	413,615
S34	TI ((befriend* or be# friend* or buddy or buddies or ((community or lay or paid or support) n1 (person or worker*)))) OR AB ((befriend* or be# friend* or buddy or buddies or ((community or lay or paid or support) n1 (person or worker*))))	6,926
S33	SU (((self n1 (administer* or assess* or attribut* or care or change or directed or efficacy or help* or guide* or instruct* or manag* or medicat* or monitor* or regulat* or reinforc* or re inforc* or support* or technique* or therap* or train* or treat*)) or selfadminister* or selfassess* or selfattribut* or selfcare or selfchange or selfdirected or selfefficacy or selfhelp* or selfguide* or selfinstruct* or selfmanag* or selfmedicat* or selfmonitor* or selfregulat* or selfreinforc* or self re inforc* or selfsupport* or selftechnique* or selftherap* or selftrain* or selftreat* or (wellness n1 (therap* or train* or treat*)))) OR TI (((self n1 (administer* or assess* or attribut* or care or change or directed or efficacy or help* or guide* or instruct* or manag* or medicat* or monitor* or regulat* or reinforc* or re inforc* or support* or technique* or therap* or train* or treat*)) or selfadminister* or selfassess* or selfattribut* or selfcare or selfchange or selfdirected or selfefficacy or selfhelp* or selfguide* or selfinstruct* or selfmanag* or selfmedicat* or selfmonitor* or selfregulat* or selfreinforc* or self re inforc* or selfsupport* or selftechnique* or selftherap* or selftrain* or selftreat* or (wellness n1 (therap* or train* or treat*)))) OR AB (((self n1 (administer* or assess* or attribut* or care or change or directed or efficacy or help* or guide* or instruct* or manag* or medicat* or monitor* or regulat* or reinforc* or re inforc* or support* or technique* or therap* or train*	163,016

	or treat*)) or selfadminister* or selfassess* or selfattribut* or selfcare or selfchange or selfdirected or selfefficacy or selfhelp* or selfguide* or selfinstruct* or selfmanag* or selfmedicat* or selfmonitor* or selfregulat* or selfreinforc* or self re inforc* or selfsupport* or selftechnique* or selftherap* or selftrain* or selftreat* or (wellness n1 (therap* or train* or treat*))))	
S32	(((((((((DE "Friendship") OR (DE "Network Therapy")) OR (DE "Social Networks" OR DE "Online Social Networks")) OR (DE "Peer Relations")) OR (DE "Peers")) OR (DE "Peer Counseling")) OR (DE "Self-Care Skills")) OR (DE "Self-Help Techniques" OR DE "Self-Management")) OR (DE "Social Support")) OR (DE "Support Groups" OR DE "Twelve Step Programs")	120,236
S31	SU psychoeducation OR AB ((psychoed* or psycho ed*)) OR TI ((psychoed* or psycho ed*))	11,472
S30	TI (((child* n2 family traumatic stress intervention) or cftsi)) OR AB (((child* n2 family traumatic stress intervention) or cftsi))	6
S29	(((con*joint or couple* or family or families or husband* or marriage* or marital* or partner* or relations* or spous* or wife or wives* or (child* n5 parent*)) n6 (counsel* or intervention* or program* or support* or therap* or treat*)) or ((couples* or family* or relations*) n1 (based or focused or led)) or ecological therap* or expressed emotion or family dynamics or family relationships)	860,043
S28	((((DE "Couples Therapy") OR (DE "Family Intervention")) OR (DE "Family Therapy" OR DE "Conjoint Therapy" OR DE "Strategic Family Therapy" OR DE "Structural Family Therapy")) OR (DE "Marriage Counseling" OR DE "Conjoint Therapy")) OR (DE "Parent Training")	39,528
S27	TI ((hg therap* or human givens)) OR AB ((hg therap* or human givens))	1,727
S26	TI counsel* OR AB counsel*	102,924
S25	DE "Counseling" OR DE "Community Counseling" OR DE "Cross Cultural Counseling" OR DE "Educational Counseling" OR DE "Genetic Counseling" OR DE "Gerontological Counseling" OR DE "Grief Counseling" OR DE "Group Counseling" OR DE "Marriage Counseling" OR DE "Microcounseling" OR DE "Multicultural Counseling" OR DE "Occupational Guidance" OR DE "Pastoral Counseling" OR DE "Peer Counseling" OR DE "Premarital Counseling" OR DE "Psychotherapeutic Counseling" OR DE "Rehabilitation Counseling" OR DE "School Counseling"	62,561
S24	TI (psychoanal* or psychosomatic*) OR AB (psychoanal* or psychosomatic*)	90,066
S23	TI (((psychodynamic or (dynamic adj (psychotherapy* or therap*)) or incident reduction) or ((brain or transcranial) n2 stimulat*) or rtms)) OR AB (((psychodynamic or (dynamic adj (psychotherapy* or therap*)) or incident reduction) or ((brain or transcranial) n2 stimulat*) or rtms))	38,235

S22	DE "Psychodynamic Psychotherapy"	3,370
S21	TI ((emdr or (eye movement n2 desensiti*))) OR AB ((emdr or (eye movement n2 desensiti*)))	1,872
S20	DE "Eye Movement Desensitization Therapy"	1,437
S19	TI debrief* OR AB debrief*	2,815
S18	DE "Debriefing (Psychological)"	283
S17	TI ((kidnet or mindful* or narrative therap*)) OR AB ((kidnet or mindful* or narrative therap*))	16,869
S16	SU (mindfulness or ((exposure or narrative) n1 therapy))	14,174
S15	TI (((presleep or presleep) n2 imagery)) OR AB (((presleep or presleep) n2 imagery))	0
S14	((imagery n2 (rehears* or re hears*)) or (((lower* or reduc*) n3 (bad dream* or nightmare*)) and (intervention* or program* or therap* or treat*)) or ((intervention* or program* or therap* or treat*) n3 nightmare*))	1,694
S13	TI ((((augmented or virtual) n2 reality) or (virtual n1 (environment or restorative)) or ((exposure or implosive or virtual reality) n2 (intervention* or program* or therap* or train*)))) OR AB ((((augmented or virtual) n2 reality) or (virtual n1 (environment or restorative)) or ((exposure or implosive or virtual reality) n2 (intervention* or program* or therap* or train*))))	13,974
S12	((DE "Exposure Therapy") OR (DE "Narrative Therapy")) OR (DE "Virtual Reality")	10,913
S11	TI ((((compassion or emotion* or emotive*) n1 (based or focused or led)) or emotional processing or ((compassion or emotion* or emotive*) n3 (coach* or intervention* or program* or therap* or treat*)))) OR AB ((((compassion or emotion* or emotive*) n1 (based or focused or led)) or emotional processing or ((compassion or emotion* or emotive*) n3 (coach* or intervention* or program* or therap* or treat*))))	25,414
S10	(DE "Emotion Focused Therapy") OR (DE "Sympathy")	3,110
S9	TI ((((behaviour* or behavior*) n2 cognitiv*) or cbt or ccbt or ((behav* or cognitive*) n3 (intervention* or manag* or program* or restructure* or therap* or treat*)) or (stress inoculation n2 (intervention* or program* or therap* or train* or treat*)) or (behav* n2 activat*) or ((trauma n1 (based or focused or led)) or exposure based or prolonged exposure))) OR AB ((((behaviour* or behavior*) n2 cognitiv*) or cbt or ccbt or ((behav* or cognitive*) n3 (intervention* or manag* or program* or restructure* or therap* or treat*)) or (stress inoculation n2 (intervention* or program* or therap* or train* or treat*))	154,978

	or (behav* n2 activat*) or ((trauma n1 (based or focused or led)) or exposure based or prolonged exposure)))	
S8	(DE "Behavior Therapy" OR DE "Aversion Therapy" OR DE "Conversion Therapy" OR DE "Dialectical Behavior Therapy" OR DE "Exposure Therapy" OR DE "Implosive Therapy" OR DE "Reciprocal Inhibition Therapy" OR DE "Response Cost" OR DE "Systematic Desensitization Therapy") OR (DE "Cognitive Behavior Therapy" OR DE "Acceptance and Commitment Therapy" OR DE "Cognitive Processing Therapy" OR DE "Prolonged Exposure Therapy")	49,013
S7	TI ((((psycholog* or psycho social* or psychosocial*) n3 (intervention* or program* or therap* or treat*)) or psychotherap* or psycho therap* or talk* therap* or therapeutic technique* or therapist* or third wave or time limited)) OR AB ((((psycholog* or psycho social* or psychosocial*) n3 (intervention* or program* or therap* or treat*)) or psychotherap* or psycho therap* or talk* therap* or therapeutic technique* or therapist* or third wave or time limited)) OR SU ((((psycholog* or psycho social* or psychosocial*) n3 (intervention* or program* or therap* or treat*)) or psychotherap* or psycho therap* or talk* therap* or therapeutic technique* or therapist* or third wave or time limited))	281,242
S6	DE "Psychotherapy"	67,719
S5	S1 OR S2 OR S3 OR S4	140,653
S4	TI ((railway spine or (rape n2 trauma*) or reexperienc* or re experienc* or torture syndrome or traumatic neuros* or traumatic stress)) OR AB ((railway spine or (rape n2 trauma*) or reexperienc* or re experienc* or torture syndrome or traumatic neuros* or traumatic stress))	19,098
S3	TI ((trauma* and (avoidance or grief or horror or death* or nightmare* or night mare* or emotion*))) OR AB ((trauma* and (avoidance or grief or horror or death* or nightmare* or night mare* or emotion*)))	23,709
S2	TI ((posttraumatic* or post traumatic* or stress disorder* or acute stress or ptsd or asd or desnos or (combat neuros* or combat syndrome or concentration camp syndrome or extreme stress or flashback* or flash back* or hypervigilan* or hypervigilen* or psych* stress or psych* trauma* or psycho?trauma* or psychotrauma*) or (posttrauma* or traumagenic* or traumatic stress*))) OR AB ((posttraumatic* or post traumatic* or stress disorder* or acute stress or ptsd or asd or desnos or (combat neuros* or combat syndrome or concentration camp syndrome or extreme stress or flashback* or flash back* or hypervigilan* or hypervigilen* or psych* stress or psych* trauma* or psycho?trauma* or psychotrauma*) or (posttrauma* or traumagenic* or traumatic stress*)))	104,519
S1	((((((((DE "Posttraumatic Stress Disorder" OR DE "Complex PTSD" OR DE "DESNOS") OR (DE "Acute Stress Disorder")) OR (DE "Combat Experience")) OR (DE "Emotional Trauma")) OR (DE "Post-Traumatic Stress")) OR (DE "Traumatic Neurosis")) OR (DE "Trauma")) OR (DE "Psychological Stress")) OR (DE "Chronic Stress")	70,398

CINAHL

#	Query	Results
S54	S51 NOT S49	11,937
S53	S51 NOT S49 Limiters - Published Date: 20180101-20191231	2,506
S52	S51 NOT S49	11,937
S51	S6 AND S50	12,028
S50	S40 OR S48	670,268
S49	MH ANIMALS NOT MH HUMANS	79,628
S48	s41 or s42 or s43 or s44 or s45 or s46 or s47	505,183
S47	TI (placebo* or random*) OR AB (placebo* or random*)	318,823
S46	TI (single blind* or double blind* or treble blind* or mask* or dummy* or singleblind* or doubleblind* or trebleblind* or tripleblind*) OR AB (single blind* or double blind* or treble blind* or mask* or dummy* or singleblind* or doubleblind* or trebleblind* or tripleblind*)	54,242
S45	TI (crossover or cross over) OR AB (crossover or cross over)	17,078
S44	TI clinical n2 trial* OR AB clinical n2 trial*	95,858
S43	(MH "Crossover Design") OR (MH "Placebos") OR (MH "Random Assignment")	78,307
S42	MW double blind* or single blind* or triple blind*	55,488
S41	(MH "Clinical Trials+")	268,307
S40	s7 or s8 or s9 or s10 or s11 or s12 or s13 or s14 or s15 or s16 or s17 or s18 or s19 or s20 or s21 or s22 or s23 or s29 or s30 or s31 or s34 or s35 or s36 or s37 or s38 or s39	236,549
S39	TI ((analy* n5 review*) or (evidence* n5 review*) or (methodol* n5 review*) or (quantativ* n5 review*) or (systematic* n5 review*)) OR AB ((analy* n5 review*) or (evidence* n5 review*) or (methodol* n5 review*) or (quantativ* n5 review*) or (systematic* n5 review*))	121,320

S38	TI ((pool* n2 results) or (combined n2 results) or (combining n2 results)) OR AB ((pool* n2 results) or (combined n2 results) or (combining n2 results))	5,060
S37	TI ((pool* n2 studies) or (combined n2 studies) or (combining n2 studies)) OR AB ((pool* n2 studies) or (combined n2 studies) or (combining n2 studies))	5,591
S36	TI ((pool* n2 trials) or (combined n2 trials) or (combining n2 trials)) OR AB ((pool* n2 trials) or (combined n2 trials) or (combining n2 trials))	1,344
S35	TI ((pool* n2 data) or (combined n2 data) or (combining n2 data)) OR AB ((pool* n2 data) or (combined n2 data) or (combining n2 data))	8,792
S34	s32 and s33	3,735
S33	TI review* AND PT review*	24,679
S32	TI analy* or assessment* or evidence* or methodol* or quantativ* or qualitativ* or systematic*	393,433
S31	TI systematic* n5 search* OR AB systematic* n5 search*	14,939
S30	TI systematic* n5 review* OR AB systematic* n5 review*	88,888
S29	(s24 or s25 or s26) and (s27 or s28)	81,701
S28	TI systematic* OR AB systematic*	133,890
S27	TX review* OR MW review* OR PT review*	4,746,766
S26	(MH "Cochrane Library")	23,041
S25	TI (bids or cochrane or embase or “index medicus” or “isi citation” or medline or psyclit or psychlit or scisearch or “science citation” or (web n2 science)) OR AB (bids or cochrane or embase or “index medicus” or “isi citation” or medline or psyclit or psychlit or scisearch or “science citation” or (web n2 science))	65,455
S24	TI (“electronic database*” or “bibliographic database*” or “computeri?ed database*” or “online database*”) OR AB (“electronic database*” or “bibliographic database*” or “computeri?ed database*” or “online database*”)	14,423
S23	(MH "Literature Review")	7,803
S22	PT (systematic*) or PT(meta*)	86,982

S21	TI (“fixed effect*” or “random effect*”) OR AB (“fixed effect*” or “random effect*”)	17,363
S20	TI (“mantel haenszel” or peto or dersimonian or “der simonian”) OR AB (“mantel haenszel” or peto or dersimonian or “der simonian”)	1,726
S19	TI (handsearch* or "hand search*" or "manual search*") OR AB (handsearch* or "hand search*" or "manual search*")	5,430
S18	AB "data extraction" or "data synthesis"	8,188
S17	AB "selection criteria"	4,257
S16	AB "relevant journals"	433
S15	AB "published studies"	5,562
S14	AB bibliograph*	6,982
S13	TI "reference list*"	21
S12	AB "reference list*"	7,455
S11	TI (“research review*” or “research integration”) OR AB (“research review*” or “research integration”)	1,467
S10	(MH "Meta Analysis")	40,851
S9	TI (metaanal* or “meta anal*” or metasynthes* or “meta synethes*”) OR AB (metaanal* or “meta anal*” or metasynthes* or “meta synethes*”)	61,476
S8	(MH "Systematic Review")	74,342
S7	(MH "Literature Searching+")	7,587
S6	s1 or s2 or s3 or s4 or s5	91,542
S5	TI ((posttraumatic* or "post traumatic*" or "stress disorder*" or "acute stress" or ptsd or asd or desnos or ("combat neuros*" or "combat syndrome" or "concentration camp syndrome" or "extreme stress" or flashback* or "flash back*" or hypervigilan* or hypervigilen* or "psych* stress" or "psych* trauma*" or psychotrauma* or psychotrauma*) or (posttrauma* or traumagenic* or "traumatic stress*"))) or AB ((posttraumatic* or "post traumatic*" or "stress disorder*" or "acute stress" or ptsd or asd or desnos or ("combat neuros*" or "combat syndrome" or "concentration camp syndrome" or "extreme stress" or flashback* or "flash back*" or hypervigilan* or hypervigilen* or "psych* stress" or "psych* trauma*" or psychotrauma* or psychotrauma*) or (posttrauma* or traumagenic* or "traumatic stress*")))	35,785

S4	TI ((trauma* and (avoidance or grief or horror or death* or nightmare* or "night mare*" or emotion*))) or AB ((trauma* and (avoidance or grief or horror or death* or nightmare* or "night mare*" or emotion*)))	11,611
S3	TI (("railway spine" or (rape near/2 trauma*) or reexperienc* or "re experienc*" or "torture syndrome" or "traumatic neuros*" or "traumatic stress")) or AB (("railway spine" or (rape near/2 trauma*) or reexperienc* or "re experienc*" or "torture syndrome" or "traumatic neuros*" or "traumatic stress"))	6,744
S2	(MH "stress, psychological")	44,491
S1	(MH "stress disorders, post-traumatic")	20,118

Cochrane Library

Search Name: anke

Date Run: 13/11/2019 09:24:52

Comment:

ID Search Hits

#1 MeSH descriptor: [Stress Disorders, Traumatic] explode all trees 2394

#2 MeSH descriptor: [Combat Disorders] explode all trees 125

#3 MeSH descriptor: [Psychological Trauma] explode all trees 57

#4 MeSH descriptor: [Stress Disorders, Post-Traumatic] explode all trees 2275

#5 MeSH descriptor: [Stress Disorders, Traumatic, Acute] explode all trees 43

#6 MeSH descriptor: [Stress, Psychological] explode all trees 5703

#7 ("railway spine" or (rape near/2 trauma*) or reexperienc* or "re experienc*" or "torture syndrome" or "traumatic neuros*" or "traumatic stress"):ti 716

#8 ("railway spine" or (rape near/2 trauma*) or reexperienc* or "re experienc*" or "torture syndrome" or "traumatic neuros*" or "traumatic stress"):ab 1672

#9 (trauma* and (avoidance or grief or horror or death* or nightmare* or "night mare*" or emotion*)):ti 162

#10 (trauma* and (avoidance or grief or horror or death* or nightmare* or "night mare*" or emotion*)):ab 2642

#11 (posttraumatic* or "post traumatic*" or "stress disorder*" or "acute stress" or ptsd or asd or desnos or ("combat neuros*" or "combat syndrome" or "concentration camp syndrome" or "extreme stress" or flashback* or "flash back*" or hypervigilan* or hypervigilen* or "psych* stress" or "psych*

trauma*" or psychotrauma* or psychotrauma*) or (posttrauma* or traumagenic* or "traumatic stress*")):ti 4046

#12 (posttraumatic* or "post traumatic*" or "stress disorder*" or "acute stress" or ptsd or asd or desnos or ("combat neuros*" or "combat syndrome" or "concentration camp syndrome" or "extreme stress" or flashback* or "flash back*" or hypervigilan* or hypervigilen* or "psych* stress" or "psych* trauma*" or psychotrauma* or psychotrauma*) or (posttrauma* or traumagenic* or "traumatic stress*")):ab 7920

#13 #1 or #2 or #3 or #4 or #5 or #6 or #7 or #8 or #9 or #10 or #11 or #12 with Publication Year from 2018 to 2019, with Cochrane Library publication date Between Jan 2018 and Dec 2019, in Trials 2413
