# Supplementary material for: Efficacy and moderators of efficacy of trauma-focused cognitive behavioural therapies in children and adolescents: protocol for an individual participant data meta-analysis from randomised trials
Source: BMJ Open. 2021 Feb 24;11(2):e047212. doi: 10.1136/bmjopen-2020-047212 (PMC7908282; doi:10.1136/bmjopen-2020-047212)
Supplement: Author's manuscript [file bmjopen-2020-047212.draft_revisions.pdf]

BMJ Open is committed to open peer review. As part of this commitment we make the peer review history of every article we publish publicly available.

When an article is published we post the peer reviewers' comments and the authors' responses online. We also post the versions of the paper that were used during peer review. These are the versions that the peer review comments apply to.

The versions of the paper that follow are the versions that were submitted during the peer review process. They are not the versions of record or the final published versions. They should not be cited or distributed as the published version of this manuscript.

BMJ Open is an open access journal and the full, final, typeset and author-corrected version of record of the manuscript is available on our site with no access controls, subscription charges or pay-per-view fees (http://bmjopen.bmj.com).

If you have any questions on BMJ Open's open peer review process please email info.bmjopen@bmj.com

BMJ Open

Efficacy and moderators of efficacy of trauma-focused cognitive behavioural therapies in children and adolescents – protocol for an individual participant data meta-analysis from randomized trials

Journal:	*BMJ Open*
Manuscript ID	bmjopen-2020-047212
Article Type:	Protocol
Date Submitted by the Author:	23-Nov-2020
Complete List of Authors:	de Haan, Anke; University of Cambridge, MRC Cognition and Brain Sciences Unit; University Children's Hospital Zurich, Department of Psychosomatics and Psychiatry<br>Hitchcock, Caitlin; University of Cambridge, MRC Cognition and Brain Sciences Unit<br>Meiser-Stedman , Richard ; University of East Anglia, Norwich Medical School, Department of Clinical Psychology and Psychological Therapies<br>Landolt, Markus; University Children's Hospital Zurich, Department of Psychosomatics and Psychiatry; University of Zurich, Department of Psychology - Division of Child and Adolescent Health Psychology<br>Kuhn, Isla; University of Cambridge, Medical Library<br>Black, Melissa; University of Cambridge, MRC Cognition and Brain Sciences Unit; Cambridgeshire and Peterborough NHS Foundation Trust (CPFT)<br>Klaus, Kristel; University of Cambridge, MRC Cognition and Brain Sciences Unit<br>Patel, Shivam; University of Cambridge, MRC Cognition and Brain Sciences Unit<br>Fisher, David; University College London, MRC Clinical Trials Unit at UCL, Institute of Clinical Trials and Methodology<br>Dalgleish, Tim; University of Cambridge, MRC Cognition and Brain Sciences Unit; Cambridgeshire and Peterborough NHS Foundation Trust (CPFT)
Keywords:	Child & adolescent psychiatry < PSYCHIATRY, Anxiety disorders < PSYCHIATRY, MENTAL HEALTH

SCHOLARONE™
Manuscripts

I, the Submitting Author has the right to grant and does grant on behalf of all authors of the Work (as defined in the below author licence), an exclusive licence and/or a non-exclusive licence for contributions from authors who are: i) UK Crown employees; ii) where BMJ has agreed a CC-BY licence shall apply, and/or iii) in accordance with the terms applicable for US Federal Government officers or employees acting as part of their official duties; on a worldwide, perpetual, irrevocable, royalty-free basis to BMJ Publishing Group Ltd ("BMJ") its licensees and where the relevant Journal is co-owned by BMJ to the co-owners of the Journal, to publish the Work in this journal and any other BMJ products and to exploit all rights, as set out in our licence.

The Submitting Author accepts and understands that any supply made under these terms is made by BMJ to the Submitting Author unless you are acting as an employee on behalf of your employer or a postgraduate student of an affiliated institution which is paying any applicable article publishing charge ("APC") for Open Access articles. Where the Submitting Author wishes to make the Work available on an Open Access basis (and intends to pay the relevant APC), the terms of reuse of such Open Access shall be governed by a Creative Commons licence – details of these licences and which Creative Commons licence will apply to this Work are set out in our licence referred to above.

Other than as permitted in any relevant BMJ Author's Self Archiving Policies, I confirm this Work has not been accepted for publication elsewhere, is not being considered for publication elsewhere and does not duplicate material already published. I confirm all authors consent to publication of this Work and authorise the granting of this licence.

For peer review only

Efficacy and moderators of efficacy of trauma-focused cognitive behavioural therapies in children and adolescents – protocol for an individual participant data meta-analysis from randomized trials

Anke de Haan[1,2,3], Caitlin Hitchcock[1], Richard Meiser-Stedman[4], Markus A. Landolt[2,3], Isla Kuhn[5], Melissa Black[1,6], Kristel Klaus[1], Shivam D. Patel[1], David J. Fisher[7] , Tim Dalgleish[1,6]

1 Medical Research Council Cognition and Brain Sciences Unit, University of Cambridge, Cambridge, UK

2 Department of Psychology - Division of Child and Adolescent Health Psychology, University of Zurich, Zurich, Switzerland

3 Department of Psychosomatics and Psychiatry, University Children's Hospital Zurich, Zurich, Switzerland

4 Department of Clinical Psychology and Psychological Therapies, Norwich Medical School, University of East Anglia, Norwich, UK

5 Medical Library, University of Cambridge, Cambridge, UK

6 Cambridgeshire and Peterborough NHS Foundation Trust (CPFT), Cambridge, UK

7 MRC Clinical Trials Unit at UCL, Institute of Clinical Trials and Methodology, University College London, London, UK

Correspondence concerning this article should be addressed to Dr. Anke de Haan, Medical Research Council Cognition and Brain Sciences Unit, University of Cambridge, 15 Chaucer Rd, Cambridge CB2 7EF, United Kingdom.

E-mail: Anke.deHaan@kispi.uzh.ch

Telephone: (+44) 01223 767528

ORCID: 0000-0002-4676-348X

For peer review only

Keywords: children and adolescents; individual participant data meta-analysis; posttraumatic stress disorder (PTSD); trauma; trauma-focused cognitive behavioural therapy

Word count: 3712 words

For peer review only

ABSTRACT

Introduction. Trauma-focused cognitive behavioural therapies are the first-line treatment for posttraumatic stress disorder (PTSD) in children and adolescents. Nevertheless, open questions remain with respect to efficacy: why does this first-line treatment not work for everyone? For whom does it work best? Individual clinical trials often do not provide sufficient statistical power to examine and substantiate moderating factors. To overcome the issue of limited power, an individual participant data meta-analysis (IPD-MA) of randomized trials evaluating forms of trauma-focused cognitive behavioural therapy in children and adolescents aged 6 to 18 years will be conducted.

Methods and analysis. We will update the National Institute for Health and Care Excellence (NICE) guideline literature search from 2018 with an electronic search in the databases PsycINFO, MEDLINE, Embase, Cochrane Central Register of Controlled Trials, and CINAHL with the terms (trauma* OR stress*) AND (cognitive therap* OR psychotherap*) AND (trial* OR review*). Electronic searches will be supplemented by a comprehensive grey literature search in archives and trial registries. Only randomized trials that used any manualised psychological treatment – that is a trauma-focused cognitive behavioural therapy for children and adolescents – will be included. The primary outcome variable will be child-reported posttraumatic stress symptoms (PTSS) post-treatment. Proxy-reports (teacher, parent, and caregiver) will be analysed separately. Secondary outcomes will include follow-up assessments of PTSS, PTSD diagnosis, and symptoms of comorbid disorders such as depression, anxiety-related, and externalizing problems. Random-effects models applying restricted maximum likelihood estimation will be used for all analyses. We will utilize the Revised Cochrane Risk of Bias tool to measure risk of bias. The analysis is registered on PROSPERO.

For peer review only

Ethics and dissemination. Results will be published in a peer-reviewed journal and presented at international conferences. The findings will enhance the future provision and development of trauma-focused cognitive behavioural therapies in children and adolescents.

PROSPERO registration number CRD42019151954.

STRENGTHS AND LIMITATIONS OF THIS STUDY

- This is the first individual participant data meta-analysis (IPD-MA) of trauma-focused cognitive behavioural therapies in children and adolescents.
- An IPD-MA can overcome statistical power issues to examine moderating factors of trauma-focused cognitive behavioural therapies in children and adolescents.
- A variety of measures for the primary and secondary outcomes will have been used in the individual trials with commensurate methodological and statistical complexities.
- Study findings will enhance the future provision and development of trauma-focused cognitive behavioural therapies in children and adolescents.

For peer review only

INTRODUCTION

Within the last two decades, research in children and adolescents has tremendously increased our knowledge about trauma-related disorders such as posttraumatic stress disorder (PTSD), the long-lasting impact of potentially traumatic events (PTEs), and the efficacy of trauma-focused therapies in younger populations. Trauma-focused cognitive behavioural therapies are the first-line treatment for PTSD in children, adolescents, and adults[1]. They are a category of psychological interventions including Trauma-focused Cognitive Behavioral Therapy (Tf-CBT[2]), Cognitive Therapy for PTSD (CT for PTSD[3]), Prolonged Exposure Therapy for Adolescents (PE-A[4]), and the child-friendly version of Narrative Exposure Therapy (KidNET[5]) (see the recent guideline from the National Institute for Health and Care Excellence [NICE][6]).

Classic meta-analyses synthesising aggregated data from randomized controlled trials (RCTs) have shown that trauma-focused cognitive behavioural therapies are effective in reducing psychological distress including PTSD in children and adolescents[7-11]. However, open questions remain with respect to clinical outcome: Why does this first-line treatment not work for everyone? For whom does it work best? Factors that might impact the efficacy of trauma-focused cognitive behavioural therapies in children and adolescents form two broad categories: treatment-related and child-related factors. Treatment-related factors may include the length of therapy, involvement of parents in the intervention, and the balance of behavioural and cognitive intervention components. Child-related factors may include the type of trauma, the severity of symptoms, comorbid diagnoses, gender, age, and other trauma-related or demographic variables. Current stand-alone RCTs invariably lack the power to explore the contribution of these factors to clinical outcomes, and have produced a mixed pattern of findings (e.g.,[12-17]). Further, classical meta-analysis, due to its reliance on summary data, is typically unable to comprehensively evaluate such moderating factors.

For peer review only

To overcome these problems of limited power we propose an individual participant data meta-analysis (IPD-MA) of randomized trials. By addressing the critical question about what works for whom, we hope to enhance the future provision and development of trauma-focused cognitive behavioural therapies in children and adolescents. In a first step, our aim is to determine the efficacy of trauma-focused cognitive behavioural therapies for children and adolescents, relative to control and active comparison conditions. A second step then addresses our central aim to explore moderators of treatment effects, both treatment-related factors and child-related factors. Both of these aims are theory-driven and of high clinical relevance for successfully treating children and adolescents who have been exposed to trauma. The following hypotheses will be examined:

- Hypothesis 1: Trauma-focused cognitive behavioural therapies will produce a greater reduction in posttraumatic stress symptoms (PTSS) in children and adolescents compared with either (1) no intervention (no treatment, waitlist), (2) treatment-as-usual (TAU), (3) individual non-trauma focused psychosocial interventions, or (4) other individual trauma-focused psychosocial interventions.

- Hypothesis 2a: Efficacy of trauma-focused cognitive behavioural therapies will be significantly predicted by pre-defined treatment-related factors available at trial baseline. Due to the mixed findings from previous studies, non-directional hypotheses will be tested. Post-treatment PTSS will be significantly predicted by:
  - Pre-defined intended length of treatment (number of sessions).
  - Pre-defined intended involvement of caregivers.

- Hypothesis 2b: Child-related factors will serve as prognostic predictors for the efficacy of trauma-focused cognitive behavioural therapies. Due to the mixed

For peer review only

findings from previous studies, non-directional hypotheses will be tested. Post-treatment PTSS will be significantly predicted by:

- Age of the participants at the start of treatment.
- Gender.
- Trauma-type of index-event.
- Trauma-history.
- Severity of PTSS pre-treatment.

For peer review only

METHODS AND ANALYSIS

Study registration and management

This IPD-MA will be conducted in accordance with Preferred Reporting Items for a Systematic Review and Meta-analysis of Individual Participant Data (The PRISMA-IPD statement[18]), and is registered on PROSPERO (CRD42019151954). Regular email updates will be sent to inform the collaborating network of study progress. End-to-end encrypted electronic data-sharing clouds and email will be used to exchange pseudo-anonymised data and paperwork between researchers.

Criteria for included studies

Types of studies

Only randomized studies will be included in this IPD-MA. Articles must be written in English. Unpublished data will be actively sought; hence, non-peer-reviewed studies will also be included. We will perform sensitivity analyses to evaluate the impact of published vs. unpublished studies on our results.

Participants

Studies must have recruited children and adolescents aged 6 to 18 years exposed to a single-event trauma (e.g. road traffic accident) or multi-event trauma (e.g. domestic violence) sufficient to meet the DSM-IV or DSM-5 definitions of a qualifying traumatic event. We will request studies with a broader age range; however, only participants within our defined age range will be included in the IPD. Sensitivity analyses will be conducted if the adult version of a treatment were administered to an adolescent sample. A standardised outcome measure comprising either a diagnostic interview indexing symptom severity or a self-report measure of PTSS must have been administered before and after treatment. Furthermore, a clinically-relevant degree of severity of PTSS at trial baseline must have been present as assessed either by scoring above a validated cut-off on a PTSS rating scale or by meeting criteria for PTSD. We will also request studies that include both children and adolescents with and without clinically relevant severity of PTSS as defined above. However, again, only those participants with clinically relevant severity of PTSS will be included in the analyses.

Treatments

In line with the NICE guideline[6], we will include studies that used any manualised psychological treatment that we deem to be a trauma-focused cognitive behavioural therapy for children and adolescents. This includes cognitive therapy, cognitive processing therapy (CPT), compassion focused therapy, exposure therapy/prolonged exposure (PE), virtual reality exposure therapy (VRET), imagery rehearsal therapy, and KidNET. We furthermore adopt the NICE guideline description of trauma-focused cognitive behavioural therapy as laid out in the associated paper (Mavranezouli et al.[10], p. 19); namely, “a broad class of psychological interventions that predominantly use trauma-focused cognitive, behavioural or cognitive behavioural techniques and exposure approaches to treatment. Although some interventions

For peer review only

place their main emphasis on exposure (e.g. imaginal reliving, producing a written narrative or in vivo exposure) and others on cognitive techniques (e.g. restructuring of trauma-related appraisals), most use a combination.” Independent raters will evaluate author descriptions of their treatment with respect to this definition to determine inclusion within the IPD-MA. In contrast to the NICE guideline[6], we will not include mindfulness-based cognitive therapy (MBCT) as a trauma-focused cognitive behavioural therapy.

In addition, treatment may be delivered in-person or online, but must comprise an individual-, rather than group-format, and a multi-session treatment protocol. A minimum of at least one post-treatment / follow-up assessment must have been reported.

For peer review only

Comparison conditions

Trauma-focused cognitive behavioural therapies will be compared: (1) against no intervention (no treatment, waitlist); (2) against TAU; (3) against individual non-trauma focused psychosocial interventions; or (4) against other individual trauma-focused psychosocial interventions. Again, comparison condition type will be determined by two independent raters, based on the author descriptions.

Primary outcomes

The primary outcome variable will be child-reported PTSS using a standardised self-report post-treatment (see paragraph “Strategy for data synthesis” for further information). Proxy-reports (teacher, parent, and caregiver) will be analysed separately. The primary endpoint of post-treatment will be indexed as the assessment completed immediately after completion of trauma-focused cognitive behavioural therapy, less than one month after the final treatment session.

Secondary outcomes

Secondary outcomes will include: a) follow-up assessments of PTSS; b) PTSD diagnoses; and, c) symptoms of comorbid disorders such as depression, anxiety-related, and externalizing problems, reported via self- and proxy-reports. Follow-up lengths to be included comprise assessments between one month and two years following the completion of therapy. During analysis, studies including a follow-up assessment between one and three months post-treatment will be grouped to form a short-term follow-up, and any later assessment points will be grouped per six month period (i.e., 6 months, 12 months). This will result in analysis of follow-up outcomes in the short-term (1-3 months), and at 6, 12, 18, and 24 months post-treatment.

Search methods for identification of studies and obtaining datasets

Figure 1 depicts our multi-layered search method in order to obtain all potential studies, published and unpublished (in line with The PRISMA-IPD statement[18]).

Please insert Figure 1 here

Figure 1. Flow-chart showing data acquisition.

IPD = individual participant data. NICE = National Institute for Health and Care Excellence.

Electronic searches

Publications identified by the latest NICE guideline for PTSD published in 2018 will be included (the NICE guideline search was completed on the 29th January 2018). To update the results of the NICE search, an electronic search using the same databases will be restricted to publications between the 1st January 2018 and 12th November 2019. We will replicate the NICE guideline search by using the same search terms related to trauma-focused cognitive

For peer review only

behavioural therapies. We will exclude specific search terms that are not related to a psychosocial intervention and thereby unlikely to include a cognitive-behavioural therapy approach. This includes physiotherapy and biological interventions (e.g., Transcranial Magnetic Stimulation). We will also exclude specific terms defining any intervention that is not cognitive behaviour therapy-based, e.g., hypnosis or dance therapy. Finally, we will remove terms referring to occupational / return to work support.

Searches in the databases PsycINFO via EBSCOhost, MEDLINE via Ovid, Embase via Ovid, Cochrane Central Register of Controlled Trials (CENTRAL) via Cochrane Library, CINAHL via EBSCOhost will use the following search plan: (trauma* OR stress*) AND (cognitive therap* OR psychotherap*) AND (trial* OR review*) (see supplementary material for the full search strategy).

No language restrictions will be applied to the search, but only studies published in English will be included. Search results will be deduplicated in Endnote (by IK), then imported into Rayyan (a web-based tool for managing systematic reviews[19]). This will allow for blind screening by raters. Two raters will independently review title and abstract of the records identified in the electronic search. The raters will review the records according to the following exclusion criteria applying the same exclusion order: 1) duplicate; 2) language other than English; 3) review or meta-analysis; 4) no randomized trial; 5) no applicable age range extractable; 6) no manualised trauma-focused cognitive behavioural therapy; 7) group-format; 8) single-session treatment; 9) no assessment post-treatment; 10) no standardized outcome measure to assess PTSS; 11) no clinically relevant PTSS extractable; 12) comparison condition outside protocol. Records deemed ineligible from title and abstract by both raters will be set aside. Records appearing eligible (i.e. not meeting any exclusion criterion) or where eligibility can not be determined due to insufficient information in the abstract will proceed to the full-

For peer review only

text stage. Again, at the full-text stage, two raters will examine the remaining records independently. Any disagreements will be resolved via discussion with RMS, MAL, and TD.

Grey literature

Clinical trial registries and archives will be searched up to the 12th November 2019 using the following search string: (child* OR adolesc* OR youth OR young*) AND (PTSD OR posttraumatic stress disorder). We will use the trial registries ClinicalTrials.gov and ISRCTN to identify any relevant unpublished trials, including those that are currently ongoing. Moreover, the archives PsyArXiv and bioRxiv will be searched to identify any relevant preprints up to 6 months prior (12th of May 2019) of the electronic search (12th of November 2019). Finally, we will check reference lists of included studies and relevant meta-analyses identified by the electronic search to make sure that all available trials will have been detected by the NICE guideline, our electronic search, and grey literature search.

Non-literature based searching

Key authors will be contacted via email to request any unpublished datasets, and Twitter will be used to raise awareness of the IPD-MA.

Data collection

Corresponding authors of eligible studies will be emailed to request data. A reminder email will be sent after two weeks. If an author does not respond after two emails, another author of the study will be contacted as well (either first, second, or last author). A second attempt to contact both authors together will follow. A maximum of three authors per article

For peer review only

will be contacted. We will consider study data unavailable if no study authors respond to multiple contact attempts, or if authors indicate that they no longer have access to the data or do not wish to make their data available. A single person for each included study will be designated to whom all queries about the data collection processes and transformation of individual variables will be addressed. When cleaning and preparing a specific data set, communication with the original investigators will take place by email or telephone.

For peer review only

Data extraction, quality checks, and storage

The primary variables to be requested from study investigators are listed in Table 1. We aim to collect data on all of these variables from all studies, regardless of whether such data were previously published. For all outcomes, unimputed and untransformed data will be requested. Data will be cleaned and stored separately for each study. Spot checks will be completed to ensure data quality. The pattern of treatment allocation for each included study will be checked to ensure that randomisation and allocation sequence appear appropriate, in accordance with guidelines recommended by Tierney et al.[20]. For final checks before analysis and the statistical analyses, the datasets will be combined into a single dataset. Data will be stored in password-protected files on an encrypted University of Cambridge server.

Table 1

Individual participant data to be extracted from included studies

**Treatment-related factors**	**Child-related factors**	**Outcomes**
Descriptives	*Demographics*	
Trial identifier	Anonymised participant identifier	
Country of completion	Gender	
Information about risk of bias	Age	
Type of trauma-focused cognitive behavioural therapy	Ethnicity	
Type of comparison group/s	Trauma type of index-event	

Number of sessions	Trauma history	
Length of treatment in weeks		
Involvement of caregivers		
Any potential covariates (e.g., mode of administration, profession of therapists)	Any potential covariates (e.g., pre-treatment levels of dysfunctional posttraumatic cognitions, IQ, social support, treatment expectancy, therapeutic alliance)	Any related outcome variables (e.g., post-treatment level of dysfunctional posttraumatic cognitions and changes in coping behaviours)
	Psychological symptoms pre-treatment	*Psychological symptoms post-treatment and follow-up*
	Pre-treatment self- and proxy-reported PTSS	Self- and proxy-reported PTSS post-treatment and follow-up
	Pre-treatment self- and proxy-reported depression symptoms	Self- and proxy-reported depression symptoms post-treatment and follow-up
	Pre-treatment self- and proxy-reported anxiety-related symptoms	Self- and proxy-reported anxiety-related symptoms post-treatment and follow-up
	Pre-treatment self- and proxy-reported externalizing problems	Self- and proxy-reported externalizing problems post-treatment and follow-up
	Diagnoses pre-treatment	*Diagnoses post-treatment and follow-up*
	Pre-treatment diagnostic status of PTSD	Diagnostic status of PTSD post-treatment and follow-up
	Pre-treatment diagnostic status of comorbid disorders	Diagnostic status of comorbid disorders post-treatment and follow-up
	Reason for missing data	Reason for missing data

Note. PTSS = posttraumatic stress symptoms. PTSD = posttraumatic stress disorder.

For peer review only

Risk of bias

Two raters will independently evaluate the risk of bias for the included studies by using the revised Cochrane Risk of Bias Tool (RoB 2[21]) to access study quality and risk of bias due to the randomisation process, deviations from intended interventions, missing outcome data, measurement of the outcome, and selection of the reported result. Each study will be rated as of high risk, some concerns, or low risk.

Strategy for data synthesis

Data will be analysed across a series of stages, in order to be guided by data availability and the degree of potential for harmonisation. Unimputed and untransformed data will be requested.

Firstly, for published studies, key variables will be re-analysed within each study (e.g., participant numbers per treatment condition, mean PTSS scores pre- and post-treatment, numbers gender, mean age), as to identify any potential inconsistencies in the supplied data.

Secondly, data will be harmonised as far as possible: (a) the definitions and scales of outcomes (e.g., standardizing PTSS total scores across different measures); (b) the timings of measurements (e.g., pre-treatment defined as the assessment directly before start of treatment; post-treatment defined as the assessment immediately after completing treatment, less than one month after the final treatment session); (c) the definitions, scales and/or subgroups used for covariates (e.g., the specific index trauma event will be grouped into accidental trauma, natural disaster, war trauma, or interpersonal trauma).

Thirdly, depending on the amount of missing data and whether missing at random assumptions are met, multiple imputation will be carried out.

For peer review only

Based on the step-wise approach described before, decisions may be made e.g. to put aside certain desired analyses or adjustment factors if it is felt that data are too limited and may bias results. Once the final constitution of the model has been agreed based on the above, we will proceed to the meta-analysis pooling itself. Note that modelling will be done for each outcome (as specified in Table 1) separately. All analyses will be completed using random-effects models employing restricted maximum likelihood estimation. However, if there is considerable heterogeneity in the quality of studies (indexed by RoB 2), a sensitivity analysis will be completed comparing random-effects and fixed-effects models.

Depending on the IPD data sets we receive, we will collect aggregate data (AD) from studies where IPD could not be obtained and combine it with the IPD to tackle inclusion bias[22]. In this case, sensitivity analyses will be performed comparing an IPD-only meta-analysis with a meta-analysis that combines IPD and AD.

One-stage approaches will be applied using the R software[23]. We will investigate the overall summary of treatment effect and we are further interested in the heterogeneity in treatment effect across- and within-studies. If the one-stage model fails to converge, a two-stage model will be calculated.

<u>Hypothesis 1</u>

A one-stage linear mixed effects (LME) model with random intercept and outcome baseline adjustment with different residual variance per study will be applied to analyse the effect of trauma-focused cognitive behavioural therapies on the continuous outcome of PTSS post-treatment. A mixed effects logistic regression model will be used to analyse the treatment effect on the binary outcome PTSD diagnosis post-treatment. Sensitivity analysis will be completed contrasting a (single) random intercept (as primary) with a separate fixed intercept

For peer review only

for each study (as sensitivity). As described before, if the one-stage model fails to converge, a two-stage model will be completed.

Hypothesis 2a

We will use meta-regression to explore whether the overall effect of trauma-focused cognitive behavioural therapies varies in relation to treatment-related factors such as pre-defined intended length of treatment and pre-defined intended involvement of caregivers.

Hypothesis 2b

To investigate whether the overall effect of trauma-focused cognitive behavioural therapies varies by child-related factors such as age, gender, trauma type of index-event, trauma-history, and symptom severity pre-treatment, subject-level interactions will be investigated. Interaction term between treatment status and subject-level covariates will be specified[22].

Random-effects distributions for the interaction effects will be specified. Effects on the continuous primary outcome of PTSS post-treatment will be analysed using a one-stage LME model with random intercept to account for correlation between the interaction estimate and other parameter estimates. Patient-level covariates will be centred to separate within-trial and across-trial effects. A mixed effects logistic regression model for the binary secondary outcome of PTSD diagnosis post-treatment will be applied. Sensitivity analysis will be completed contrasting a (single) random intercept (as primary) with a separate fixed intercept for each study (as sensitivity). As described before, if the one-stage model fails to converge, a two-stage model will be completed.

For peer review only

Additional analyses with sub-samples

Depending on the data provided, we will investigate further treatment-related factors such as pre-defined mode of administration, profession of therapists, and treatment expectancy pre-treatment. Moreover, additional child-related factors such as comorbidity pre-treatment, pre-treatment levels of dysfunctional posttraumatic cognitions, IQ, and pre-treatment parental mental health will be addressed.

Future analyses

In future investigations of the obtained data, we plan to conduct mediation analyses within the IPD-MA context to evaluate mechanisms of action of trauma-focused cognitive behavioural therapies. The most promising candidates seem to be changes in targeted cognitive and behavioural processes; for example, improvements in dysfunctional posttraumatic cognitions of the child regarding being permanently and disturbingly changed, or feeling vulnerable[24-26], as well as changes in safety-seeking behaviours[25].

Moreover, we are aiming to investigate non-responding, deterioration, and possible predictors for dropping out from trauma-focused cognitive behavioural therapies across studies.

For peer review only

ETHICS AND DISSEMINATION

We will cite the ethics code for each contributing study in the published paper. Contributing studies will be required to remove patient identifiers before providing their data. This includes names, addresses, and date of birth which will be converted to age at index trauma event and age at time of assessment. Contributing studies need to have permission to share anonymised data.

For peer review only

PATIENT AND PUBLIC INVOLVEMENT

Patients were not involved in this study. However, secondary data analysis ensures maximum return from patient involvement in research. The outcome of this IPD-MA will be published in an international peer-reviewed journal. The findings will further be presented at international conferences.

AUTHORS' CONTRIBUTIONS

AdH, CH, RMS, MAL, and TD designed the project. IK, MB, KK, SDP, and DJF contributed to study methods. All authors critically reviewed and approved the final manuscript.

COMPETING INTERESTS STATEMENT

The authors declare that they have no competing interests.

For peer review only

FUNDING STATEMENT

Anke de Haan is funded by the Swiss National Science Foundation (Grant Reference: P2ZHP1_187612). Caitlin Hitchcock and Shivam D. Patel are partly supported by the Economic and Social Research Council (Grant Reference: ES/R010781/1). Melissa Black is partly supported by the National Institute for Health Research Cambridge Biomedical Research Centre. Kristel Klaus is funded by the UK Medical Research Council (Grant Reference: MC_PC_17213). David J. Fisher is funded by the UK Medical Research Council (Grant Reference: MC_UU_12023/24). Tim Dalgleish is funded by the UK Medical Research Council (Grant Reference: SUAG/043 G101400) and partly supported by the National Institute for Health Research Cambridge Biomedical Research Centre.

REFERENCES

1. Forbes D, Creamer M, Bisson JI, et al. A guide to guidelines for the treatment of PTSD and related conditions. *J Trauma Stress* 2010;23(5):537-52. doi: 10.1002/jts.20565
2. Cohen JA, Mannarino AP, Deblinger E. Treating trauma and traumatic grief in children and adolescents. New York, NY, US: Guilford Press, 2006.
3. Smith P, Perrin S, Yule W, et al. Post traumatic stress disorder: cognitive therapy with children and young people. London: Routledge 2010.
4. Foa EB, Chrestman K, Gilboa-Schechtman E. Prolonged exposure manual for children and adolescents suffering from PTSD. New York, NY, US: Oxford University Press 2008.
5. Schauer M, Neuner F, Elbert T. Narrative Exposure Therapy for Children and Adolescents (KIDNET). In: Landolt MA, Cloitre M, Schnyder U, eds. Evidence-based treatments for trauma related disorders in children and adolescents. Cham: Springer International Publishing 2017:227-50.
6. National Institute for Health and Care Excellence. Post-traumatic stress disorder NICE guideline [NG116]. 2018. https://www.nice.org.uk/guidance/ng116/evidence (accessed 10.2019).
7. Dorsey S, McLaughlin KA, Kerns SEU, et al. Evidence base update for psychosocial treatments for children and adolescents exposed to traumatic events. *J Clin Child Adolesc Psychol* 2017;46(3):303-30. doi: 10.1080/15374416.2016.1220309
8. Gutermann J, Schreiber F, Matulis S, et al. Psychological treatments for symptoms of posttraumatic stress disorder in children, adolescents, and young adults: a meta-analysis. *Clin Child Fam Psychol Rev* 2016;19:77–93. doi: 10.1007/s10567-016-0202-5
9. Lenz AS, Hollenbaugh KM. Meta-analysis of trauma-focused cognitive behavioral therapy for treating PTSD and co-occurring depression among children and adolescents.

For peer review only

Counseling Outcome Research and Evaluation 2015;6(1):18-32. doi: 10.1177/2150137815573790

10. Mavranezouli I, Megnin-Viggars O, Daly C, et al. Psychological and psychosocial treatments for children and young people with post-traumatic stress disorder: a network meta-analysis. *J Child Psychol Psychiatry* 2020;61(1):18-29. doi: 10.1111/jcpp.13094
11. Morina N, Koerssen R, Pollet TV. Interventions for children and adolescents with posttraumatic stress disorder: a meta-analysis of comparative outcome studies. *Clin Psychol Rev* 2016;47:41-54. doi: 10.1016/j.cpr.2016.05.006
12. Deblinger E, Mannarino AP, Cohen JA, et al. Trauma-focused cognitive behavioral therapy for children: impact of the trauma narrative and treatment length. *Depress Anxiety* 2011;28(1):67-75. doi: 10.1002/da.20744
13. Goldbeck L, Muche R, Sachser C, et al. Effectiveness of trauma-focused cognitive behavioral therapy for children and adolescents: a randomized controlled trial in eight German mental health clinics. *Psychother Psychosom* 2016;85(3):159-70.
14. Knutsen M, Jensen TK. Changes in the trauma narratives of youth receiving trauma-focused cognitive behavioral therapy in relation to posttraumatic stress symptoms. *Psychother Res* 2019;29(1):99-111. doi: 10.1080/10503307.2017.1303208
15. Ormhaug SM, Jensen TK, Wentzel-Larsen T, et al. The therapeutic alliance in treatment of traumatized youths: relation to outcome in a randomized clinical trial. *J Consult Clin Psychol* 2014;82(1):52-64. doi: 10.1037/a0033884
16. Ready CB, Hayes AM, Yasinski CW, et al. Overgeneralized beliefs, accommodation, and treatment outcome in youth receiving trauma-focused cognitive behavioral therapy for childhood trauma. *Behav Ther* 2015;46(5):671-88. doi: 10.1016/j.beth.2015.03.004
17. Yasinski C, Hayes AM, Ready CB, et al. In-session caregiver behavior predicts symptom change in youth receiving trauma-focused cognitive behavioral therapy (TF-CBT). *J Consult Clin Psychol* 2016;84(12):1066-77. doi: 10.1037/ccp0000147

For peer review only

18. Stewart LA, Clarke M, Rovers M, et al. Preferred reporting items for systematic review and meta-analyses of individual participant data: the PRISMA-IPD statement. *JAMA* 2015;313(16):1657-65. doi: 10.1001/jama.2015.3656

19. Ouzzani M, Hammady H, Fedorowicz Z, et al. Rayyan - a web and mobile app for systematic reviews. *Syst Rev* 2016;5(1):210. doi: 10.1186/s13643-016-0384-4

20. Tierney JF, Vale C, Riley R, et al. Individual participant data (IPD) meta-analyses of randomised controlled trials: guidance on their use. *PLoS Med* 2015;12(7):e1001855. doi: 10.1371/journal.pmed.1001855

21. Sterne JAC, Savović J, Page MJ, et al. RoB 2: a revised tool for assessing risk of bias in randomised trials. *BMJ* 2019;366:l4898. doi: 10.1136/bmj.l4898

22. Debray TP, Moons KG, van Valkenhoef G, et al. Get real in individual participant data (IPD) meta-analysis: a review of the methodology. *Res Synth Methods* 2015;6(4):293-309. doi: 10.1002/jrsm.1160

23. R Core Team. R: a language and environment for statistical computing. Vienna: R Foundation for Statistical Computing 2020.

24. Jensen TK, Holt T, Mørup Ormhaug S, et al. Change in post-traumatic cognitions mediates treatment effects for traumatized youth - a randomized controlled trial. *J Couns Psychol* 2018;65(2):166-77. doi: 10.1037/cou0000258

25. Meiser-Stedman R, Smith P, McKinnon A, et al. Cognitive therapy as an early treatment for post-traumatic stress disorder in children and adolescents: a randomized controlled trial addressing preliminary efficacy and mechanisms of action. *J Child Psychol Psychiatry* 2017;58(5):623-33. doi: 10.1111/jcpp.12673

26. Pfeiffer E, Sachser C, de Haan A, et al. Dysfunctional posttraumatic cognitions as a mediator of symptom reduction in trauma-focused cognitive behavioral therapy with children and adolescents: results of a randomized controlled trial. *Behav Res Ther* 2017;97:178-82. doi: 10.1016/j.brat.2017.08.001

For peer review only

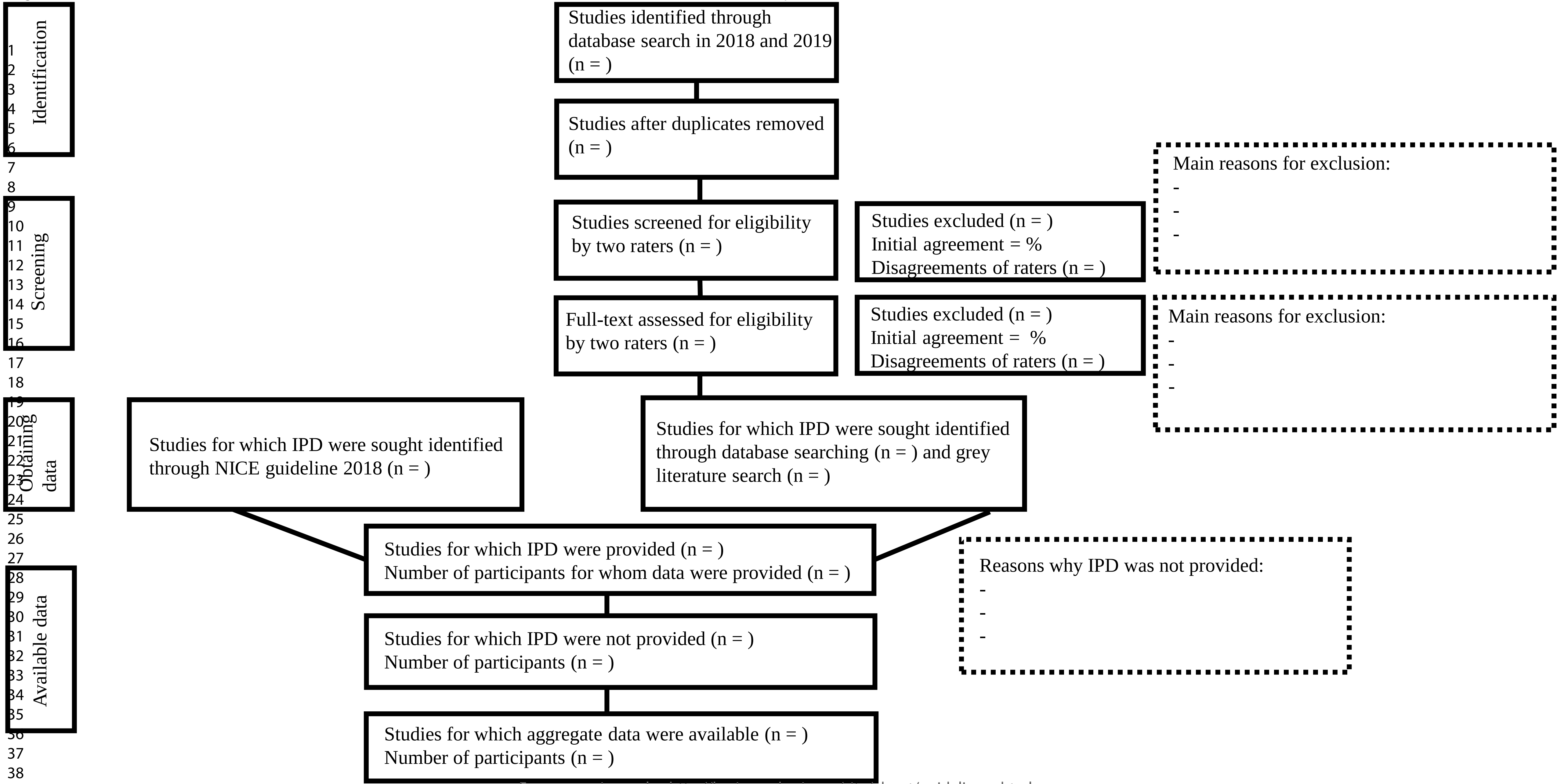
Identification
Screening
Obtaining data
Available data
Studies identified through database search in 2018 and 2019 (n =)
Studies after duplicates removed (n =)
Main reasons for exclusion:
-
-
-
Studies screened for eligibility by two raters (n =)
Studies excluded (n =)
Initial agreement = %
Disagreements of raters (n =)
Full-text assessed for eligibility by two raters (n =)
Studies excluded (n =)
Initial agreement = %
Disagreements of raters (n =)
Main reasons for exclusion:
-
-
-
Studies for which IPD were sought identified through NICE guideline 2018 (n =)
Studies for which IPD were sought identified through database searching (n =) and grey literature search (n =)
Studies for which IPD were provided (n =)
Number of participants for whom data were provided (n =)
Reasons why IPD was not provided:
-
-
-
Studies for which IPD were not provided (n =)
Number of participants (n =)
Studies for which aggregate data were available (n =)
Number of participants (n =)

Search strategy de Haan et al.

Database	Number of hits
Medline	1913
Embase	2000
CINAHL	2506
Cochrane	2413
PsycINFO	2105
Total	10937
Total deduplicated	7091

Searches run 12th November 2019

Limited by publication date 2018 to date

Medline

Database: Ovid MEDLINE(R) and Epub Ahead of Print, In-Process & Other Non-Indexed Citations, Daily and Versions(R) <1946 to November 08, 2019>

Search Strategy:

----

1 stress disorders, traumatic/ or combat disorders/ or psychological trauma/ or stress disorders, post-traumatic/ or stress disorders, traumatic, acute/ or stress, psychological/ (147421)

2 (railway spine or (rape adj2 trauma*) or reexperienc* or re experienc* or torture syndrome or traumatic neuros* or traumatic stress).ti,ab. (15096)

3 (trauma* and (avoidance or grief or horror or death* or nightmare* or night mare* or emotion*)).ti,ab. (35178)

4 (posttraumatic* or post traumatic* or stress disorder* or acute stress or ptsd or asd or desnos or (combat neuros* or combat syndrome or concentration camp syndrome or extreme stress or flashback* or flash back* or hypervigilan* or hypervigilen* or psych* stress or psych* trauma* or psycho?trauma* or psychotrauma*) or (posttrauma* or traumagenic* or traumatic stress*)).ti,ab. (106800)

5 or/1-4 (245567)

6 psychotherapy/ (53279)

7 (((psycholog* or psycho social* or psychosocial*) adj3 (intervention* or program* or therap* or treat*)) or psychotherap* or psycho therap* or talk* therap* or therapeutic technique* or therapist* or third wave or time limited).ti,ab,sh. (144180)

8 exp behavior therapy/ (72171)

9 (((behaviour* or behavior*) adj2 cognitiv*) or cbt or ccbt or ((behav* or cognitive*) adj3 (intervention* or manag* or program* or restructure* or therap* or treat*)) or (stress inoculation adj2 (intervention* or program* or therap* or train* or treat*)) or (behav* adj2 activat*) or ((trauma adj (based or focused or led)) or exposure based or prolonged exposure)).ti,ab. (115271)

10 emotions/ (63893)

For peer review only - http://bmjopen.bmj.com/site/about/guidelines.xhtml

11 (((compassion or emotion* or emotive*) adj (based or focused or led)) or emotional processing or ((compassion or emotion* or emotive*) adj3 (coach* or intervention* orprogram* or therap* or treat*))).ti,ab. (7907)

12 implosive therapy/ or narrative therapy/ or virtual reality exposure therapy/ (1886)

13 (((augmented or virtual) adj2 reality) or (virtual adj (environment or restorative)) or ((exposure or implosive or virtual reality) adj2 (intervention* or program* or therap* or train*))).ti,ab. (16289)

14 ((imagery adj2 (rehears* or re hears*)) or (((lower* or reduc*) adj3 (bad dream* or nightmare*)) and (intervention* or program* or therap* or treat*)) or ((intervention* or program* or therap* or treat*) adj3 nightmare*)).mp. or ((presleep or presleep) adj2 imagery).ti,ab. (267)

15 (mindfulness or ((exposure or narrative) adj therapy)).sh. (2743)

16 (kidnet or mindful* or narrative therap*).ti,ab. (8835)

17 debrief*.ti,ab. (3803)

18 eye movement desensitization reprocessing/ (205)

19 (emdr or (eye movement adj2 desensiti*)).ti,ab. (671)

20 psychotherapy, psychodynamic/ (529)

21 (psychodynamic or (dynamic adj (psychotherapy* or therap*)) or incident reduction or ((brain or transcranial) adj2 stimulat*) or rtms).ti,ab. (36027)

22 (psychoanal* or psychosomatic*).ti,ab. (26156)

23 exp counseling/ (42669)

24 counsel*.ti,ab. (103170)

25 (hg therap* or human givens).ti,ab. (14)

26 couples therapy/ or family therapy/ or marital therapy/ or exp parents/ed (22720)

27 (((con?joint or couple* or family or families or husband* or marriage* or marital* or partner* or relations* or spous* or wife or wives* or (child* adj5 parent*)) adj6 (counsel* or intervention* or program* or support* or therap* or treat*)) or ((couples* or family* or relations*) adj (based or focused or led)) or ecological therap* or expressed emotion or family dynamics or family relationships).tw. (171018)

28 ((child* adj2 family traumatic stress intervention) or cftsi).ti,ab. (2)

29 psychoeducation.sh. or (psychoed* or psycho ed*).ti,ab. (5975)

30 community networks/ or friends/ or exp peer group/ or self care/ or self-help groups/ or social networking/ or social support/ (133084)

31 ((self adj (administer* or assess* or attribut* or care or change or directed or efficacy or help* or guide* or instruct* or manag* or medicat* or monitor* or regulat* or reinforc* or re inforc* or support* or technique* or therap* or train* or treat*)) or selfadminister* or selfassess* or selfattribut* or selfcare or selfchange or selfdirected or selfefficacy or selfhelp* or selfguide* or selfinstruct* or selfmanag* or selfmedicat* or selfmonitor* or selfregulat* or selfreinforc* or self re inforc* or selfsupport* or selftechnique* or selftherap* or selftrain* or selftreat* or (wellness adj (therap* or train* or treat*))).ti,ab,sh. (139336)

32 (befriend* or be*1 friend* or buddy or buddies or ((community or lay or paid or support) adj (person or worker*))).ti,ab. (3080)

33 (((consumer* or famil* or friend* or lay or mutual* or peer* or social* or spous* or voluntary or volunteer*) adj3 (assist* or advice* or advis* or counsel* or educat* or forum* or help* or mentor* or network* or support* or visit*)) or ((consumer* or famil* or peer* or self help or social* or support* or voluntary or volunteer*) adj2 group*) or ((consumer* or famil* or friend* or lay or mutual* or peer* or self

For peer review only

help or social* or spous* or support* or voluntary or volunteer*) adj3 (intervention* or program* or rehab* or therap* or service* or skill* or treat*)) or (((consumer* or famil* or friend* or lay* or peer* or spous* or user* or support* or voluntary or volunteer*) adj (based or counsel* or deliver* or interact* or led or mediat* or operated or provides or provider* or run*)) or ((consumer* or famil* or friend* or lay* or peer* or relation* or spous* or support*) adj3 trust*) or voluntary work*)).ti,ab. (314043)

34 (((lay or peer*) adj3 (advis* or consultant or educator* or expert* or facilitator* or instructor* or leader* or mentor* or person* or tutor* or worker*)) or expert patient* or mutual aid).ti,ab. (6617)

35 (peer* adj3 (assist* or counsel* or educat* or program* or rehab* or service* or supervis*)).ti,ab. (5735)

36 ((psychoeducat* or psycho educat*) adj3 (group or network* or service*)).ti,ab. (774)

37 ((psychosocial or social) adj work*).ti,ab. (15927)

38 ((ptsd or posttrauma* or post trauma* or trauma*) adj2 support*).ti,ab. (1565)

39 recovery support.ti,ab. (173)

40 assisted living facilities/ or emergency shelter/ or group homes/ or halfway houses/ or housing/ or independent living/ or residential facilities/ (30265)

41 ((resident* or hous* or accommod* or commun* or comu* or home*) adj5 (support* or support* or shelter* or outreach* or visit* or appointment*)).ti,ab. (50036)

42 (residential treatm* or residential facility* or supported hous* or public hous*).ti,ab. (3249)

43 (accomod* or assertive community treatment* or home* or housing* or outreach* or residential*).ti,ab. (525321)

44 placement.ti,ab. (122669)

45 or/6-44 (1653237)

46 meta analysis.sh,pt. or "meta-analysis as topic"/ or "review literature as topic"/ (129841)

47 (exp databases, bibliographic/ or (((electronic or computer* or online) adj database*) or bids or cochrane or embase or index medicus or isi citation or medline or psyclit or psychlit or scisearch or science citation or (web adj2 science)).ti,ab.) and (review*.ti,ab,sh,pt. or systematic*.ti,ab.) (163499)

48 ((analy* or assessment* or evidence* or methodol* or quantativ* or systematic*) adj2 (overview* or review*)).tw. or ((analy* or assessment* or evidence* or methodol* or quantativ* or systematic*).ti. and review*.ti,pt.) or (systematic* adj2 search*).ti,ab. (329117)

49 (metaanal* or meta anal* or (research adj (review* or integration))).ti,ab. or reference list*.ab. or bibliograph*.ab. or published studies.ab. or relevant journals.ab. or selection criteria.ab. or (data adj (extraction or synthesis)).ab. or (handsearch* or ((hand or manual) adj search*)).ti,ab. or (mantel haenszel or peto or dersimonian or der simonian).ti,ab. or (fixed effect* or random effect*).ti,ab. or ((pool* or combined or combining) adj2 (data or trials or studies or results)).ti,ab. (300023)

50 or/46-49 (562928)

51 exp clinical trial/ or exp "clinical trials as topic"/ or cross-over studies/ or double-blind method/ or placebos/ or random allocation/ or single-blind method/ (1194973)

52 ((clinical adj2 trial*) or (crossover or cross over)).ti,ab. (429760)

53 (((single* or doubl* or trebl* or tripl*) adj2 blind*) or mask* or dummy or doubleblind* or singleblind* or trebleblind* or tripleblind*).ti,ab. (240549)

54 (placebo* or random*).ti,ab. (1162532)

55 or/51-54 (2043304)

For peer review only

56 5 and 45 and (50 or 55) (12008)

57 animals/ not human*.mp. (4342443)

58 56 not 57 (11757)

59 limit 58 to yr="2018 -Current" (1913)

****

Embase

For peer review only

Database: Embase <1974 to 2019 November 08>

Search Strategy:

----

1 *acute stress/ or *behavioural stress/ or *emotional stress/ or *critical incident stress/ or *mental stress/ or *posttraumatic stress disorder/ or *psychotrauma/ (69082)

2 (trauma* and (avoidance or grief or horror or death* or nightmare* or night mare* or emotion*)).ti,ab. (50168)

3 (railway spine or (rape adj2 trauma*) or reexperienc* or re experienc* or torture syndrome or traumatic neuros* or traumatic stress).ti,ab. (20306)

4 (posttraumatic* or post traumatic* or stress disorder* or acute stress or ptsd or asd or desnos or (combat neuros* or combat syndrome or concentration camp syndrome or extreme stress or flashback* or flash back* or hypervigilan* or hypervigilen* or psych* stress or psych* trauma* or psycho?trauma* or psychotrauma*) or (posttrauma* or traumagenic* or traumatic stress*)).ti,ab. (139168)

5 or/1-4 (212899)

6 *psychotherapy/ (38266)

7 exp *behavior therapy/ or exp *cognitive therapy/ (29422)

8 (((psycholog* or psycho social* or psychosocial*) adj3 (intervention* or program* or therap* or treat*)) or psychotherap* or psycho therap* or talk* therap* or therapeutic technique* or therapist* or third wave or time limited).ti,ab,sh. (246754)

9 (((behaviour* or behavior*) adj2 cognitiv*) or cbt or ccbt or ((behav* or cognitive*) adj3 (intervention* or manag* or program* or restructure* or therap* or treat*)) or (stress inoculation adj2 (intervention* or program* or therap* or train* or treat*)) or (behav* adj2 activat*) or ((trauma adj (based or focused or led)) or exposure based or prolonged exposure)).ti,ab. (158532)

10 *emotion/ (28919)

11 (((compassion or emotion* or emotive*) adj (based or focused or led)) or emotional processing or ((compassion or emotion* or emotive*) adj3 (coach* or intervention* orprogram* or therap* or treat*))).ti,ab. (10888)

12 exposure therapy/ or narrative therapy/ or virtual reality exposure therapy/ (1602)

13 (((augmented or virtual) adj2 reality) or (virtual adj (environment or restorative)) or ((exposure or implosive or virtual reality) adj2 (intervention* or program* or therap* or train*))).ti,ab. (21776)

14 ((imagery adj2 (rehears* or re hears*)) or (((lower* or reduc*) adj3 (bad dream* or nightmare*)) and (intervention* or program* or therap* or treat*)) or ((intervention* or program* or therap* or treat*) adj3 nightmare*)).mp. or ((presleep or presleep) adj2 imagery).ti,ab. (454)

15 (mindfulness or ((exposure or narrative) adj therapy)).sh. (23980)

16 (kidnet or mindful* or narrative therap*).ti,ab. (12154)

17 debrief*.ti,ab. (6509)

18 (emdr or (eye movement adj2 desensiti*)).ti,ab. (925)

19 psychodynamic psychotherapy/ (1214)

20 (psychodynamic or (dynamic adj (psychotherapy* or therap*)) or incident reduction or ((brain or transcranial) adj2 stimulat*) or rtms).ti,ab. (53209)

21 (psychoanal* or psychosomatic*).ti,ab. (35159)

22 exp counseling/ (159666)

23 counsel*.ti,ab. (148163)

24 (hg therap* or human givens).ti,ab. (21)

25 couple therapy/ or family therapy/ or marital therapy/ or exp parent/ (246712)

26 (((con?joint or couple* or family or families or husband* or marriage* or marital* or partner* or relations* or spous* or wife or wives* or (child* adj5 parent*)) adj6 (counsel* or intervention* or program* or support* or therap* or treat*)) or ((couples* or family* or relations*) adj (based or focused or led)) or ecological therap* or expressed emotion or family dynamics or family relationships).tw. (226599)

27 ((child* adj2 family traumatic stress intervention) or cftsi).ti,ab. (6)

28 psychoeducation.sh. or (psychoed* or psycho ed*).ti,ab. (12914)

29 friendship/ or peer counseling/ or peer group/ or self help/ or self care/ or social network/ or social support/ or support group/ (193307)

30 ((self adj (administer* or assess* or attribut* or care or change or directed or efficacy or help* or guide* or instruct* or manag* or medicat* or monitor* or regulat* or reinforc* or re inforc* or support* or technique* or therap* or train* or treat*)) or selfadminister* or selfassess* or selfattribut* or selfcare or selfchange or selfdirected or selfefficacy or selfhelp* or selfguide* or selfinstruct* or selfmanag* or selfmedicat* or selfmonitor* or selfregulat* or selfreinforc* or self re inforc* or selfsupport* or selftechnique* or selftherap* or selftrain* or selftreat* or (wellness adj (therap* or train* or treat*))).ti,ab,sh. (188037)

31 (befriend* or be*1 friend* or buddy or buddies or ((community or lay or paid or support) adj (person or worker*))).ti,ab. (4130)

32 (((consumer* or famil* or friend* or lay or mutual* or peer* or social* or spous* or voluntary or volunteer*) adj3 (assist* or advice* or advis* or counsel* or educat* or forum* or help* or mentor* or network* or support* or visit*)) or ((consumer* or famil* or peer* or self help or social* or support* or voluntary or volunteer*) adj2 group*) or ((consumer* or famil* or friend* or lay or mutual* or peer* or self help or social* or spous* or support* or voluntary or volunteer*) adj3 (intervention* or program* or rehab* or therap* or service* or skill* or treat*)) or (((consumer* or famil* or friend* or lay* or peer* or spous* or user* or support* or voluntary or volunteer*) adj (based or counsel* or deliver* or interact* or led or mediat* or operated or provides or provider* or run*)) or ((consumer* or famil* or friend* or lay* or peer* or relation* or spous* or support*) adj3 trust*) or voluntary work*)).ti,ab. (410161)

33 (((lay or peer*) adj3 (advis* or consultant or educator* or expert* or facilitator* or instructor* or leader* or mentor* or person* or tutor* or worker*)) or expert patient* or mutual aid).ti,ab. (8679)

34 (peer* adj3 (assist* or counsel* or educat* or program* or rehab* or service* or supervis*)).ti,ab. (7548)

For peer review only

35 ((psychoeducat* or psycho educat*) adj3 (group or network* or service*)).ti,ab. (1226)

36 ((psychosocial or social) adj work*).ti,ab. (22915)

37 ((ptsd or posttrauma* or post trauma* or trauma*) adj2 support*).ti,ab. (1890)

38 recovery support.ti,ab. (216)

39 assisted living facility/ or emergency shelter/ or halfway house/ or housing/ or independent living/ or residential home/ or residential home/ (36467)

40 ((resident* or hous* or accommod* or commun* or comu* or home*) adj5 (support* or support* or shelter* or outreach* or visit* or appointment*)).ti,ab. (66837)

41 (residential treatm* or residential facility* or supported hous* or public hous*).ti,ab. (3836)

42 (accomod* or assertive community treatment* or home* or housing* or outreach* or residential*).ti,ab. (693104)

43 placement.ti,ab. (174618)

44 or/6-43 (2352266)

45 meta analysis/ or "meta analysis (topic)"/ or systematic review/ (349285)

46 (exp bibliographic database/ or (((electronic or computer* or online) adj database*) or bids or cochrane or embase or index medicus or isi citation or medline or psyclit or psychlit orscisearch or science citation or (web adj2 science)).ti,ab.) and (review*.ti,ab,sh,pt. or systematic*.ti,ab.) (223431)

47 ((analy* or assessment* or evidence* or methodol* or quantativ* or systematic*) adj2 (overview* or review*)).tw. or ((analy* or assessment* or evidence* or methodol* or quantativ* or systematic*).ti. and review*.ti,pt.) or (systematic* adj2 search*).ti,ab. (392909)

48 (metaanal* or meta anal* or (research adj (review* or integration))).ti,ab. or reference list*.ab. or bibliograph*.ab. or published studies.ab. or relevant journals.ab. or selection criteria.ab. or (data adj (extraction or synthesis)).ab. or (handsearch* or ((hand or manual) adj search*)).ti,ab. or (mantel haenszel or peto or dersimonian or der simonian).ti,ab. or (fixed effect* or random effect*).ti,ab. or ((pool* or combined or combining) adj2 (data or trials or studies or results)).ti,ab. (393628)

49 or/45-48 (775067)

50 exp "clinical trial (topic)"/ or exp clinical trial/ or crossover procedure/ or double blind procedure/ or placebo/ or randomization/ or random sample/ or single blind procedure/ (1962363)

51 (clinical adj2 trial*).ti,ab. (504366)

52 (crossover or cross over).ti,ab. (101345)

53 (((single* or doubl* or trebl* or tripl*) adj2 blind*) or mask* or dummy or doubleblind* or singleblind* or trebleblind* or tripleblind*).ti,ab. (323907)

54 (placebo* or random*).ti,ab. (1578946)

55 or/50-54 (2965134)

56 5 and 44 and (49 or 55) (10225)

57 animals/ not human*.mp. (857762)

58 56 not 57 (10209)

59 limit 58 to yr="2018 -Current" (2000)

For peer review only

****

PsycINFO

#	Query	Results
S64	s59 not s60 Limiters - Publication Year: 2018-2020	2,105
S63		277,708
S62		1,761,949
S61	s59 not s60	16,053
S60	animal not human	289,401
S59	S5 AND S48 AND S58	16,256
S58	S56 OR S57	765,475
S57	((clinical trials or placebo or random sampling)) OR (clinical adj2 trial*) OR (crossover or cross over) OR ((((single* or doubl* or trebl* or tripl*) n2 blind*) or mask* or dummy or doubleblind* or singleblind* or trebleblind* or tripleblind*)) OR (placebo* or random*) OR treatment outcome*	347,654
S56	S49 OR S50 OR S51 OR S52 OR S53 OR S54 OR S55	468,514
S55	(metaanal* or meta anal* or (research n1 (review* or integration))) OR (reference list* or bibliograph* or published studies or relevant journals or selection criteria) OR ((data n1 (extraction or synthesis))) OR ((handsearch* or ((hand or manual) n1 search*))) OR ((mantel haenszel or peto or dersimonian or der simonian)) OR ((fixed effect* or random effect*)) OR (((pool* or combined or combining) n2 (data or trials or studies or results)))	142,371
S54	(systematic* n2 search*)	6,258
S53	(analy* or assessment* or evidence* or methodol* or quantativ* or systematic*) AND review*	297,715
S52	((analy* or assessment* or evidence* or methodol* or quantativ* or systematic*) n2 (overview* or review*))	317,006
S51	(((electronic or computer* or online) n1 database*) or bids or cochrane or embase or index medicus or isi citation or medline or psyclit or psychlit or scisearch or science citation or (web n2 science)) AND (review* or systematic*)	53,766
S50	computer searching	1,605

For peer review only

S49	((literature review or meta analysis)) OR systematic review	138,174
S48	S6 OR S7 OR S8 OR S9 OR S10 OR S11 OR S12 OR S13 OR S14 OR S15 OR S16 OR S17 OR S18 OR S19 OR S20 OR S21 OR S22 OR S23 OR S24 OR S25 OR S26 OR S27 OR S28 OR S29 OR S30 OR S31 OR S32 OR S33 OR S34 OR S35 OR S36 OR S37 OR S38 OR S39 OR S40 OR S41 OR S42 OR S43 OR S44 OR S45 OR S46 OR S47	1,682,227
S47	TI (placement or student placement) OR AB placement	26,717
S46	TI ((accomod* or assertive community treatment* or home* or housing* or outreach* or residential*)) OR AB ((accomod* or assertive community treatment* or home* or housing* or outreach* or residential*))	190,252
S45	TI ((residential treatm* or residential facility* or supported hous* or public hous*)) OR AB ((residential treatm* or residential facility* or supported hous* or public hous*))	9,115
S44	TI (((resident* or hous* or accommod* or commun* or comu* or home*) n5 (support* or support* or shelter* or outreach* or visit* or appointment*))) OR AB (((resident* or hous* or accommod* or commun* or comu* or home*) n5 (support* or support* or shelter* or outreach* or visit* or appointment*)))	39,683
S43	TI (((resident* or hous* or accommod* or commun* or comu* or home*) adj5 (support* or support* or shelter* or outreach* or visit* or appointment*))) OR AB (((resident* or hous* or accommod* or commun* or comu* or home*) adj5 (support* or support* or shelter* or outreach* or visit* or appointment*)))	0
S42	(((((DE "Assisted Living") OR (DE "Shelters")) OR (DE "Group Homes")) OR (DE "Halfway Houses")) OR (DE "Housing")) OR (DE "Residential Care Institutions")	19,622
S41	TI recovery support OR AB recovery support	2,144
S40	TI (((ptsd or posttrauma* or post trauma* or trauma*) n2 support*)) OR AB (((ptsd or posttrauma* or post trauma* or trauma*) n2 support*))	1,570
S39	TI (((psychosocial or social) n1 work*)) OR AB (((psychosocial or social) n1 work*))	48,187
S38	TI (((psychoeducat* or psycho educat*) n3 (group or network* or service*))) OR AB (((psychoeducat* or psycho educat*) n3 (group or network* or service*)))	1,967
S37	TI ((peer* n3 (assist* or counsel* or educat* or program* or rehab* or service* or supervis*))) OR AB ((peer* n3 (assist* or counsel* or educat* or program* or rehab* or service* or supervis*)))	7,910
S36	TI ((((lay or peer*) n3 (advis* or consultant or educator* or expert* or facilitator* or instructor* or leader* or mentor* or person* or tutor* or	10,195

For peer review only

	worker*)) or expert patient* or mutual aid)) OR AB ((((lay or peer*) n3 (advis* or consultant or educator* or expert* or facilitator* or instructor* or leader* or mentor* or person* or tutor* or worker*)) or expert patient* or mutual aid))	
S35	TI ((((consumer* or famil* or friend* or lay or mutual* or peer* or social* or spous* or voluntary or volunteer*) n3 (assist* or advice* or advis* or counsel* or educat* or forum* or help* or mentor* or network* or support* or visit*)) or ((consumer* or famil* or peer* or self help or social* or support* or voluntary or volunteer*) n2 group*) or ((consumer* or famil* or friend* or lay or mutual* or peer* or self help or social* or spous* or support* or voluntary or volunteer*) n3 (intervention* or program* or rehab* or therap* or service* or skill* or treat*)) or (((consumer* or famil* or friend* or lay* or peer* or spous* or user* or support* or voluntary or volunteer*) n1 (based or counsel* or deliver* or interact* or led or mediat* or operated or provides or provider* or run*)) or ((consumer* or famil* or friend* or lay* or peer* or relation* or spous* or support*) n3 trust*) or voluntary work*))) OR AB ((((consumer* or famil* or friend* or lay or mutual* or peer* or social* or spous* or voluntary or volunteer*) n3 (assist* or advice* or advis* or counsel* or educat* or forum* or help* or mentor* or network* or support* or visit*)) or ((consumer* or famil* or peer* or self help or social* or support* or voluntary or volunteer*) n2 group*) or ((consumer* or famil* or friend* or lay or mutual* or peer* or self help or social* or spous* or support* or voluntary or volunteer*) n3 (intervention* or program* or rehab* or therap* or service* or skill* or treat*)) or (((consumer* or famil* or friend* or lay* or peer* or spous* or user* or support* or voluntary or volunteer*) n1 (based or counsel* or deliver* or interact* or led or mediat* or operated or provides or provider* or run*)) or ((consumer* or famil* or friend* or lay* or peer* or relation* or spous* or support*) n3 trust*) or voluntary work*)))	413,615
S34	TI ((befriend* or be# friend* or buddy or buddies or ((community or lay or paid or support) n1 (person or worker*)))) OR AB ((befriend* or be# friend* or buddy or buddies or ((community or lay or paid or support) n1 (person or worker*))))	6,926
S33	SU (((self n1 (administer* or assess* or attribut* or care or change or directed or efficacy or help* or guide* or instruct* or manag* or medicat* or monitor* or regulat* or reinforc* or re inforc* or support* or technique* or therap* or train* or treat*)) or selfadminister* or selfassess* or selfattribut* or selfcare or selfchange or selfdirected or selfefficacy or selfhelp* or selfguide* or selfinstruct* or selfmanag* or selfmedicat* or selfmonitor* or selfregulat* or selfreinforc* or self re inforc* or selfsupport* or selftechnique* or selftherap* or selftrain* or selftreat* or (wellness n1 (therap* or train* or treat*)))) OR TI (((self n1 (administer* or assess* or attribut* or care or change or directed or efficacy or help* or guide* or instruct* or manag* or medicat* or monitor* or regulat* or reinforc* or re inforc* or support* or technique* or therap* or train* or treat*)) or selfadminister* or selfassess* or selfattribut* or selfcare or selfchange or selfdirected or selfefficacy or selfhelp* or selfguide* or selfinstruct* or selfmanag* or selfmedicat* or selfmonitor* or selfregulat* or selfreinforc* or self re inforc* or selfsupport* or selftechnique* or selftherap* or selftrain* or selftreat* or (wellness n1 (therap* or train* or treat*)))) OR AB (((self n1 (administer* or assess* or attribut* or care or change or directed or efficacy or help* or guide* or instruct* or manag* or medicat* or monitor* or regulat* or reinforc* or re inforc* or support* or technique* or therap* or train*	163,016

For peer review only

	or treat*)) or selfadminister* or selfassess* or selfattribut* or selfcare or selfchange or selfdirected or selfefficacy or selfhelp* or selfguide* or selfinstruct* or selfmanag* or selfmedicat* or selfmonitor* or selfregulat* or selfreinforc* or self re inforc* or selfsupport* or selftechnique* or selftherap* or selftrain* or selftreat* or (wellness n1 (therap* or train* or treat*))))	
S32	((((((((DE "Friendship") OR (DE "Network Therapy")) OR (DE "Social Networks" OR DE "Online Social Networks")) OR (DE "Peer Relations")) OR (DE "Peers")) OR (DE "Peer Counseling")) OR (DE "Self-Care Skills")) OR (DE "Self-Help Techniques" OR DE "Self-Management")) OR (DE "Social Support")) OR (DE "Support Groups" OR DE "Twelve Step Programs")	120,236
S31	SU psychoeducation OR AB ((psychoed* or psycho ed*)) OR TI ((psychoed* or psycho ed*))	11,472
S30	TI (((child* n2 family traumatic stress intervention) or cftsi)) OR AB (((child* n2 family traumatic stress intervention) or cftsi))	6
S29	(((con*joint or couple* or family or families or husband* or marriage* or marital* or partner* or relations* or spous* or wife or wives* or (child* n5 parent*)) n6 (counsel* or intervention* or program* or support* or therap* or treat*)) or ((couples* or family* or relations*) n1 (based or focused or led)) or ecological therap* or expressed emotion or family dynamics or family relationships)	860,043
S28	((((DE "Couples Therapy") OR (DE "Family Intervention")) OR (DE "Family Therapy" OR DE "Conjoint Therapy" OR DE "Strategic Family Therapy" OR DE "Structural Family Therapy")) OR (DE "Marriage Counseling" OR DE "Conjoint Therapy")) OR (DE "Parent Training")	39,528
S27	TI ((hg therap* or human givens)) OR AB ((hg therap* or human givens))	1,727
S26	TI counsel* OR AB counsel*	102,924
S25	DE "Counseling" OR DE "Community Counseling" OR DE "Cross Cultural Counseling" OR DE "Educational Counseling" OR DE "Genetic Counseling" OR DE "Gerontological Counseling" OR DE "Grief Counseling" OR DE "Group Counseling" OR DE "Marriage Counseling" OR DE "Microcounseling" OR DE "Multicultural Counseling" OR DE "Occupational Guidance" OR DE "Pastoral Counseling" OR DE "Peer Counseling" OR DE "Premarital Counseling" OR DE "Psychotherapeutic Counseling" OR DE "Rehabilitation Counseling" OR DE "School Counseling"	62,561
S24	TI (psychoanal* or psychosomatic*) OR AB (psychoanal* or psychosomatic*)	90,066
S23	TI (((psychodynamic or (dynamic adj (psychotherapy* or therap*)) or incident reduction) or ((brain or transcranial) n2 stimulat*) or rtms)) OR AB (((psychodynamic or (dynamic adj (psychotherapy* or therap*)) or incident reduction) or ((brain or transcranial) n2 stimulat*) or rtms))	38,235

For peer review only

S22	DE "Psychodynamic Psychotherapy"	3,370
S21	TI ((emdr or (eye movement n2 desensiti*))) OR AB ((emdr or (eye movement n2 desensiti*)))	1,872
S20	DE "Eye Movement Desensitization Therapy"	1,437
S19	TI debrief* OR AB debrief*	2,815
S18	DE "Debriefing (Psychological)"	283
S17	TI ((kidnet or mindful* or narrative therap*)) OR AB ((kidnet or mindful* or narrative therap*))	16,869
S16	SU (mindfulness or ((exposure or narrative) n1 therapy))	14,174
S15	TI (((presleep or presleep) n2 imagery)) OR AB (((presleep or presleep) n2 imagery))	0
S14	((imagery n2 (rehears* or re hears*)) or (((lower* or reduc*) n3 (bad dream* or nightmare*)) and (intervention* or program* or therap* or treat*)) or ((intervention* or program* or therap* or treat*) n3 nightmare*))	1,694
S13	TI ((((augmented or virtual) n2 reality) or (virtual n1 (environment or restorative)) or ((exposure or implosive or virtual reality) n2 (intervention* or program* or therap* or train*)))) OR AB ((((augmented or virtual) n2 reality) or (virtual n1 (environment or restorative)) or ((exposure or implosive or virtual reality) n2 (intervention* or program* or therap* or train*))))	13,974
S12	((DE "Exposure Therapy") OR (DE "Narrative Therapy")) OR (DE "Virtual Reality")	10,913
S11	TI ((((compassion or emotion* or emotive*) n1 (based or focused or led)) or emotional processing or ((compassion or emotion* or emotive*) n3 (coach* or intervention* or program* or therap* or treat*)))) OR AB ((((compassion or emotion* or emotive*) n1 (based or focused or led)) or emotional processing or ((compassion or emotion* or emotive*) n3 (coach* or intervention* or program* or therap* or treat*))))	25,414
S10	(DE "Emotion Focused Therapy") OR (DE "Sympathy")	3,110
S9	TI ((((behaviour* or behavior*) n2 cognitiv*) or cbt or ccbt or ((behav* or cognitive*) n3 (intervention* or manag* or program* or restructure* or therap* or treat*)) or (stress inoculation n2 (intervention* or program* or therap* or train* or treat*)) or (behav* n2 activat*) or ((trauma n1 (based or focused or led)) or exposure based or prolonged exposure))) OR AB ((((behaviour* or behavior*) n2 cognitiv*) or cbt or ccbt or ((behav* or cognitive*) n3 (intervention* or manag* or program* or restructure* or therap* or treat*)) or (stress inoculation n2 (intervention* or program* or therap* or train* or treat*))	154,978

For peer review only

	or (behav* n2 activat*) or ((trauma n1 (based or focused or led)) or exposure based or prolonged exposure)))	
S8	(DE "Behavior Therapy" OR DE "Aversion Therapy" OR DE "Conversion Therapy" OR DE "Dialectical Behavior Therapy" OR DE "Exposure Therapy" OR DE "Implosive Therapy" OR DE "Reciprocal Inhibition Therapy" OR DE "Response Cost" OR DE "Systematic Desensitization Therapy") OR (DE "Cognitive Behavior Therapy" OR DE "Acceptance and Commitment Therapy" OR DE "Cognitive Processing Therapy" OR DE "Prolonged Exposure Therapy")	49,013
S7	TI ((((psycholog* or psycho social* or psychosocial*) n3 (intervention* or program* or therap* or treat*)) or psychotherap* or psycho therap* or talk* therap* or therapeutic technique* or therapist* or third wave or time limited)) OR AB ((((psycholog* or psycho social* or psychosocial*) n3 (intervention* or program* or therap* or treat*)) or psychotherap* or psycho therap* or talk* therap* or therapeutic technique* or therapist* or third wave or time limited)) OR SU ((((psycholog* or psycho social* or psychosocial*) n3 (intervention* or program* or therap* or treat*)) or psychotherap* or psycho therap* or talk* therap* or therapeutic technique* or therapist* or third wave or time limited))	281,242
S6	DE "Psychotherapy"	67,719
S5	S1 OR S2 OR S3 OR S4	140,653
S4	TI ((railway spine or (rape n2 trauma*) or reexperienc* or re experienc* or torture syndrome or traumatic neuros* or traumatic stress)) OR AB ((railway spine or (rape n2 trauma*) or reexperienc* or re experienc* or torture syndrome or traumatic neuros* or traumatic stress))	19,098
S3	TI ((trauma* and (avoidance or grief or horror or death* or nightmare* or night mare* or emotion*))) OR AB ((trauma* and (avoidance or grief or horror or death* or nightmare* or night mare* or emotion*)))	23,709
S2	TI ((posttraumatic* or post traumatic* or stress disorder* or acute stress or ptsd or asd or desnos or (combat neuros* or combat syndrome or concentration camp syndrome or extreme stress or flashback* or flash back* or hypervigilan* or hypervigilen* or psych* stress or psych* trauma* or psycho?trauma* or psychotrauma*) or (posttrauma* or traumagenic* or traumatic stress*))) OR AB ((posttraumatic* or post traumatic* or stress disorder* or acute stress or ptsd or asd or desnos or (combat neuros* or combat syndrome or concentration camp syndrome or extreme stress or flashback* or flash back* or hypervigilan* or hypervigilen* or psych* stress or psych* trauma* or psycho?trauma* or psychotrauma*) or (posttrauma* or traumagenic* or traumatic stress*)))	104,519
S1	(((((((DE "Posttraumatic Stress Disorder" OR DE "Complex PTSD" OR DE "DESNOS") OR (DE "Acute Stress Disorder")) OR (DE "Combat Experience")) OR (DE "Emotional Trauma")) OR (DE "Post-Traumatic Stress")) OR (DE "Traumatic Neurosis")) OR (DE "Trauma")) OR (DE "Psychological Stress")) OR (DE "Chronic Stress")	70,398

For peer review only

CINAHL

#	Query	Results
S54	S51 NOT S49	11,937
S53	S51 NOT S49 Limiters - Published Date: 20180101-20191231	2,506
S52	S51 NOT S49	11,937
S51	S6 AND S50	12,028
S50	S40 OR S48	670,268
S49	MH ANIMALS NOT MH HUMANS	79,628
S48	s41 or s42 or s43 or s44 or s45 or s46 or s47	505,183
S47	TI (placebo* or random*) OR AB (placebo* or random*)	318,823
S46	TI (single blind* or double blind* or treble blind* or mask* or dummy* or singleblind* or doubleblind* or trebleblind* or tripleblind*) OR AB (single blind* or double blind* or treble blind* or mask* or dummy* or singleblind* or doubleblind* or trebleblind* or tripleblind*)	54,242
S45	TI (crossover or cross over) OR AB (crossover or cross over)	17,078
S44	TI clinical n2 trial* OR AB clinical n2 trial*	95,858
S43	(MH "Crossover Design") OR (MH "Placebos") OR (MH "Random Assignment")	78,307
S42	MW double blind* or single blind* or triple blind*	55,488
S41	(MH "Clinical Trials+")	268,307
S40	s7 or s8 or s9 or s10 or s11 or s12 or s13 or s14 or s15 or s16 or s17 or s18 or s19 or s20 or s21 or s22 or s23 or s29 or s30 or s31 or s34 or s35 or s36 or s37 or s38 or s39	236,549
S39	TI ((analy* n5 review*) or (evidence* n5 review*) or (methodol* n5 review*) or (quantativ* n5 review*) or (systematic* n5 review*)) OR AB ((analy* n5 review*) or (evidence* n5 review*) or (methodol* n5 review*) or (quantativ* n5 review*) or (systematic* n5 review*))	121,320

For peer review only

S38	TI ((pool* n2 results) or (combined n2 results) or (combining n2 results)) OR AB ((pool* n2 results) or (combined n2 results) or (combining n2 results))	5,060
S37	TI ((pool* n2 studies) or (combined n2 studies) or (combining n2 studies)) OR AB ((pool* n2 studies) or (combined n2 studies) or (combining n2 studies))	5,591
S36	TI ((pool* n2 trials) or (combined n2 trials) or (combining n2 trials)) OR AB ((pool* n2 trials) or (combined n2 trials) or (combining n2 trials))	1,344
S35	TI ((pool* n2 data) or (combined n2 data) or (combining n2 data)) OR AB ((pool* n2 data) or (combined n2 data) or (combining n2 data))	8,792
S34	s32 and s33	3,735
S33	TI review* AND PT review*	24,679
S32	TI analy* or assessment* or evidence* or methodol* or quantativ* or qualitativ* or systematic*	393,433
S31	TI systematic* n5 search* OR AB systematic* n5 search*	14,939
S30	TI systematic* n5 review* OR AB systematic* n5 review*	88,888
S29	(s24 or s25 or s26) and (s27 or s28)	81,701
S28	TI systematic* OR AB systematic*	133,890
S27	TX review* OR MW review* OR PT review*	4,746,766
S26	(MH "Cochrane Library")	23,041
S25	TI (bids or cochrane or embase or “index medicus” or “isi citation” or medline or psyclit or psychlit or scisearch or “science citation” or (web n2 science)) OR AB (bids or cochrane or embase or “index medicus” or “isi citation” or medline or psyclit or psychlit or scisearch or “science citation” or (web n2 science))	65,455
S24	TI (“electronic database*” or “bibliographic database*” or “computeri?ed database*” or “online database*”) OR AB (“electronic database*” or “bibliographic database*” or “computeri?ed database*” or “online database*”)	14,423
S23	(MH "Literature Review")	7,803
S22	PT (systematic*) or PT(meta*)	86,982

For peer review only

S21	TI (“fixed effect*” or “random effect*”) OR AB (“fixed effect*” or “random effect*”)	17,363
S20	TI (“mantel haenszel” or peto or dersimonian or “der simonian”) OR AB (“mantel haenszel” or peto or dersimonian or “der simonian”)	1,726
S19	TI (handsearch* or "hand search*" or "manual search*") OR AB (handsearch* or "hand search*" or "manual search*")	5,430
S18	AB "data extraction" or "data synthesis"	8,188
S17	AB "selection criteria"	4,257
S16	AB "relevant journals"	433
S15	AB "published studies"	5,562
S14	AB bibliograph*	6,982
S13	TI "reference list*"	21
S12	AB "reference list*"	7,455
S11	TI (“research review*” or “research integration”) OR AB (“research review*” or “research integration”)	1,467
S10	(MH "Meta Analysis")	40,851
S9	TI (metaanal* or “meta anal*” or metasynthes* or “meta synethes*”) OR AB (metaanal* or “meta anal*” or metasynthes* or “meta synethes*”)	61,476
S8	(MH "Systematic Review")	74,342
S7	(MH "Literature Searching+")	7,587
S6	s1 or s2 or s3 or s4 or s5	91,542
S5	TI ((posttraumatic* or "post traumatic*" or "stress disorder*" or "acute stress" or ptsd or asd or desnos or ("combat neuros*" or "combat syndrome" or "concentration camp syndrome" or "extreme stress" or flashback* or "flash back*" or hypervigilan* or hypervigilen* or "psych* stress" or "psych* trauma*" or psychotrauma* or psychotrauma*) or (posttrauma* or traumagenic* or "traumatic stress*"))) or AB ((posttraumatic* or "post traumatic*" or "stress disorder*" or "acute stress" or ptsd or asd or desnos or ("combat neuros*" or "combat syndrome" or "concentration camp syndrome" or "extreme stress" or flashback* or "flash back*" or hypervigilan* or hypervigilen* or "psych* stress" or "psych* trauma*" or psychotrauma* or psychotrauma*) or (posttrauma* or traumagenic* or "traumatic stress*")))	35,785

For peer review only

S4	TI ((trauma* and (avoidance or grief or horror or death* or nightmare* or "night mare*" or emotion*))) or AB ((trauma* and (avoidance or grief or horror or death* or nightmare* or "night mare*" or emotion*)))	11,611
S3	TI (("railway spine" or (rape near/2 trauma*) or reexperienc* or "re experienc*" or "torture syndrome" or "traumatic neuros*" or "traumatic stress")) or AB (("railway spine" or (rape near/2 trauma*) or reexperienc* or "re experienc*" or "torture syndrome" or "traumatic neuros*" or "traumatic stress"))	6,744
S2	(MH "stress, psychological")	44,491
S1	(MH "stress disorders, post-traumatic")	20,118

Cochrane Library

Search Name: anke

Date Run: 13/11/2019 09:24:52

Comment:

ID Search Hits

#1 MeSH descriptor: [Stress Disorders, Traumatic] explode all trees 2394

#2 MeSH descriptor: [Combat Disorders] explode all trees 125

#3 MeSH descriptor: [Psychological Trauma] explode all trees 57

#4 MeSH descriptor: [Stress Disorders, Post-Traumatic] explode all trees 2275

#5 MeSH descriptor: [Stress Disorders, Traumatic, Acute] explode all trees 43

#6 MeSH descriptor: [Stress, Psychological] explode all trees 5703

#7 ("railway spine" or (rape near/2 trauma*) or reexperienc* or "re experienc*" or "torture syndrome" or "traumatic neuros*" or "traumatic stress"):ti 716

#8 ("railway spine" or (rape near/2 trauma*) or reexperienc* or "re experienc*" or "torture syndrome" or "traumatic neuros*" or "traumatic stress"):ab 1672

#9 (trauma* and (avoidance or grief or horror or death* or nightmare* or "night mare*" or emotion*)):ti 162

#10 (trauma* and (avoidance or grief or horror or death* or nightmare* or "night mare*" or emotion*)):ab 2642

#11 (posttraumatic* or "post traumatic*" or "stress disorder*" or "acute stress" or ptsd or asd or desnos or ("combat neuros*" or "combat syndrome" or "concentration camp syndrome" or "extreme stress" or flashback* or "flash back*" or hypervigilan* or hypervigilen* or "psych* stress" or "psych*

For peer review only

trauma*" or psychotrauma* or psychotrauma*) or (posttrauma* or traumagenic* or "traumatic stress*")):ti 4046

#12 (posttraumatic* or "post traumatic*" or "stress disorder*" or "acute stress" or ptsd or asd or desnos or ("combat neuros*" or "combat syndrome" or "concentration camp syndrome" or "extreme stress" or flashback* or "flash back*" or hypervigilan* or hypervigilen* or "psych* stress" or "psych* trauma*" or psychotrauma* or psychotrauma*) or (posttrauma* or traumagenic* or "traumatic stress*")):ab 7920

#13 #1 or #2 or #3 or #4 or #5 or #6 or #7 or #8 or #9 or #10 or #11 or #12 with Publication Year from 2018 to 2019, with Cochrane Library publication date Between Jan 2018 and Dec 2019, in Trials 2413

For peer review only

Efficacy and moderators of efficacy of trauma-focused cognitive behavioural therapies in children and adolescents – protocol for an individual participant data meta-analysis from randomized trials

Journal:	*BMJ Open*
Manuscript ID	bmjopen-2020-047212.R1
Article Type:	Protocol
Date Submitted by the Author:	29-Jan-2021
Complete List of Authors:	de Haan, Anke; University of Cambridge, MRC Cognition and Brain Sciences Unit; University Children's Hospital Zurich, Department of Psychosomatics and Psychiatry<br>Hitchcock, Caitlin; University of Cambridge, MRC Cognition and Brain Sciences Unit<br>Meiser-Stedman , Richard ; University of East Anglia, Norwich Medical School, Department of Clinical Psychology and Psychological Therapies<br>Landolt, Markus; University Children's Hospital Zurich, Department of Psychosomatics and Psychiatry; University of Zurich, Department of Psychology - Division of Child and Adolescent Health Psychology<br>Kuhn, Isla; University of Cambridge, Medical Library<br>Black, Melissa; University of Cambridge, MRC Cognition and Brain Sciences Unit; Cambridgeshire and Peterborough NHS Foundation Trust (CPFT)<br>Klaus, Kristel; University of Cambridge, MRC Cognition and Brain Sciences Unit<br>Patel, Shivam; University of Cambridge, MRC Cognition and Brain Sciences Unit<br>Fisher, David; University College London, MRC Clinical Trials Unit at UCL, Institute of Clinical Trials and Methodology<br>Dalgleish, Tim; University of Cambridge, MRC Cognition and Brain Sciences Unit; Cambridgeshire and Peterborough NHS Foundation Trust (CPFT)
**Primary Subject Heading**:	Mental health
Secondary Subject Heading:	Evidence based practice, Paediatrics
Keywords:	Child & adolescent psychiatry < PSYCHIATRY, Anxiety disorders < PSYCHIATRY, MENTAL HEALTH

For peer review only - http://bmjopen.bmj.com/site/about/guidelines.xhtml

I, the Submitting Author has the right to grant and does grant on behalf of all authors of the Work (as defined in the below author licence), an exclusive licence and/or a non-exclusive licence for contributions from authors who are: i) UK Crown employees; ii) where BMJ has agreed a CC-BY licence shall apply, and/or iii) in accordance with the terms applicable for US Federal Government officers or employees acting as part of their official duties; on a worldwide, perpetual, irrevocable, royalty-free basis to BMJ Publishing Group Ltd ("BMJ") its licensees and where the relevant Journal is co-owned by BMJ to the co-owners of the Journal, to publish the Work in this journal and any other BMJ products and to exploit all rights, as set out in our licence.

The Submitting Author accepts and understands that any supply made under these terms is made by BMJ to the Submitting Author unless you are acting as an employee on behalf of your employer or a postgraduate student of an affiliated institution which is paying any applicable article publishing charge ("APC") for Open Access articles. Where the Submitting Author wishes to make the Work available on an Open Access basis (and intends to pay the relevant APC), the terms of reuse of such Open Access shall be governed by a Creative Commons licence – details of these licences and which Creative Commons licence will apply to this Work are set out in our licence referred to above.

Other than as permitted in any relevant BMJ Author's Self Archiving Policies, I confirm this Work has not been accepted for publication elsewhere, is not being considered for publication elsewhere and does not duplicate material already published. I confirm all authors consent to publication of this Work and authorise the granting of this licence.

For peer review only

Efficacy and moderators of efficacy of trauma-focused cognitive behavioural therapies in children and adolescents – protocol for an individual participant data meta-analysis from randomized trials

Anke de Haan[1,2,3], Caitlin Hitchcock[1], Richard Meiser-Stedman[4], Markus A. Landolt[2,3], Isla Kuhn[5], Melissa Black[1,6], Kristel Klaus[1], Shivam D. Patel[1], David J. Fisher[7] , Tim Dalgleish[1,6]

1 Medical Research Council Cognition and Brain Sciences Unit, University of Cambridge, Cambridge, UK

2 Department of Psychology - Division of Child and Adolescent Health Psychology, University of Zurich, Zurich, Switzerland

3 Department of Psychosomatics and Psychiatry, University Children's Hospital Zurich, Zurich, Switzerland

4 Department of Clinical Psychology and Psychological Therapies, Norwich Medical School, University of East Anglia, Norwich, UK

5 Medical Library, University of Cambridge, Cambridge, UK

6 Cambridgeshire and Peterborough NHS Foundation Trust (CPFT), Cambridge, UK

7 MRC Clinical Trials Unit at UCL, Institute of Clinical Trials and Methodology, University College London, London, UK

Correspondence concerning this article should be addressed to Dr. Anke de Haan, Medical Research Council Cognition and Brain Sciences Unit, University of Cambridge, 15 Chaucer Rd, Cambridge CB2 7EF, United Kingdom.

E-mail: Anke.deHaan@kispi.uzh.ch

Telephone: (+44) 01223 767528

ORCID: 0000-0002-4676-348X

For peer review only

Keywords: children and adolescents; individual participant data meta-analysis; posttraumatic stress disorder (PTSD); trauma; trauma-focused cognitive behavioural therapy

Word count: 3439 words

For peer review only

ABSTRACT

Introduction. Trauma-focused cognitive behavioural therapies are the first-line treatment for posttraumatic stress disorder (PTSD) in children and adolescents. Nevertheless, open questions remain with respect to efficacy: why does this first-line treatment not work for everyone? For whom does it work best? Individual clinical trials often do not provide sufficient statistical power to examine and substantiate moderating factors. To overcome the issue of limited power, an individual participant data meta-analysis (IPD-MA) of randomized trials evaluating forms of trauma-focused cognitive behavioural therapy in children and adolescents aged 6 to 18 years will be conducted.

Methods and analysis. We will update the National Institute for Health and Care Excellence (NICE) guideline literature search from 2018 with an electronic search in the databases PsycINFO, MEDLINE, Embase, Cochrane Central Register of Controlled Trials, and CINAHL with the terms (trauma* OR stress*) AND (cognitive therap* OR psychotherap*) AND (trial* OR review*). Electronic searches will be supplemented by a comprehensive grey literature search in archives and trial registries. Only randomized trials that used any manualised psychological treatment – that is a trauma-focused cognitive behavioural therapy for children and adolescents – will be included. The primary outcome variable will be child-reported posttraumatic stress symptoms (PTSS) post-treatment. Proxy-reports (teacher, parent, and caregiver) will be analysed separately. Secondary outcomes will include follow-up assessments of PTSS, PTSD diagnosis, and symptoms of comorbid disorders such as depression, anxiety-related, and externalizing problems. Random-effects models applying restricted maximum likelihood estimation will be used for all analyses. We will utilize the Revised Cochrane Risk of Bias tool to measure risk of bias.

Ethics and dissemination. Contributing study authors need to have permission to share anonymised data. Contributing studies will be required to remove patient identifiers before

For peer review only

providing their data. Results will be published in a peer-reviewed journal and presented at international conferences.

PROSPERO registration number CRD42019151954.

STRENGTHS AND LIMITATIONS OF THIS STUDY

- This is the first individual participant data meta-analysis (IPD-MA) of trauma-focused cognitive behavioural therapies in children and adolescents.
- In contrast to existing individual studies, an IPD-MA will provide the statistical power to examine moderating factors of trauma-focused cognitive behavioural therapies in children and adolescents.
- Only randomized controlled trials will be included to allow us to evaluate the efficacy of trauma-focused cognitive behavioural therapies over and above the non-specific effects of comparator conditions.
- A variety of measures of the primary and secondary outcomes will have been used in the individual studies bringing commensurate methodological and statistical complexity.
- Study findings will enhance the future provision and development of trauma-focused cognitive behavioural therapies in children and adolescents.

For peer review only

INTRODUCTION

Within the last two decades, research in children and adolescents has tremendously increased our knowledge about trauma-related disorders such as posttraumatic stress disorder (PTSD), the long-lasting impact of potentially traumatic events (PTEs), and the efficacy of trauma-focused therapies in younger populations. Trauma-focused cognitive behavioural therapies are the first-line treatment for PTSD in children, adolescents, and adults[1]. They are a category of psychological interventions including Trauma-focused Cognitive Behavioral Therapy (Tf-CBT[2]), Cognitive Therapy for PTSD (CT for PTSD[3]), Prolonged Exposure Therapy for Adolescents (PE-A[4]), and the child-friendly version of Narrative Exposure Therapy (KidNET[5]) (see the recent guideline from the National Institute for Health and Care Excellence [NICE][6]).

Classic meta-analyses synthesising aggregated data from randomized controlled trials (RCTs) have shown that trauma-focused cognitive behavioural therapies are effective in reducing psychological distress including PTSD in children and adolescents[7-11]. However, open questions remain with respect to clinical outcome: Why does this first-line treatment not work for everyone? For whom does it work best? Factors that might impact the efficacy of trauma-focused cognitive behavioural therapies in children and adolescents form two broad categories: treatment-related and child-related factors. Treatment-related factors may include the length of therapy, involvement of parents in the intervention, and the balance of behavioural and cognitive intervention components. Child-related factors may include the type of trauma, the severity of symptoms, comorbid diagnoses, gender, age, and other trauma-related or demographic variables. Current stand-alone RCTs invariably lack the power to explore the contribution of these factors to clinical outcomes, and have produced a mixed pattern of findings (e.g.,[12-17]). Further, classical meta-analysis, due to its reliance on summary data, is typically unable to comprehensively evaluate such moderating factors.

For peer review only - http://bmjopen.bmj.com/site/about/guidelines.xhtml

To overcome these problems of limited power we propose an individual participant data meta-analysis (IPD-MA) of randomized trials. By addressing the critical question about what works for whom, we hope to enhance the future provision and development of trauma-focused cognitive behavioural therapies in children and adolescents. In a first step, our aim is to determine the efficacy of trauma-focused cognitive behavioural therapies for children and adolescents, relative to control and active comparison conditions. A second step then addresses our central aim to explore moderators of treatment effects, both treatment-related factors and child-related factors. Both of these aims are theory-driven and of high clinical relevance for successfully treating children and adolescents who have been exposed to trauma. The following hypotheses will be examined:

- Hypothesis 1: Trauma-focused cognitive behavioural therapies will produce a greater reduction in posttraumatic stress symptoms (PTSS) in children and adolescents compared with either (1) no intervention (no treatment, waitlist), (2) treatment-as-usual (TAU), (3) individual non-trauma focused psychosocial interventions, or (4) other individual trauma-focused psychosocial interventions.

- Hypothesis 2a: Efficacy of trauma-focused cognitive behavioural therapies will be significantly predicted by pre-defined treatment-related factors available at trial baseline. Due to the mixed findings from previous studies, non-directional hypotheses will be tested. Post-treatment PTSS will be significantly predicted by:
  - Pre-defined intended length of treatment (number of sessions).
  - Pre-defined intended involvement of caregivers.

- Hypothesis 2b: Child-related factors will serve as prognostic predictors for the efficacy of trauma-focused cognitive behavioural therapies. Due to the mixed

For peer review only

findings from previous studies, non-directional hypotheses will be tested. Post-treatment PTSS will be significantly predicted by:

- Age of the participants at the start of treatment.
- Gender.
- Trauma-type of index-event.
- Trauma-history.
- Severity of PTSS pre-treatment.

For peer review only

METHODS AND ANALYSIS

Study registration and management

This IPD-MA will be conducted in accordance with Preferred Reporting Items for a Systematic Review and Meta-analysis of Individual Participant Data (The PRISMA-IPD statement[18]), and is registered on PROSPERO (CRD42019151954). Regular email updates will be sent to inform the collaborating network of study progress. End-to-end encrypted electronic data-sharing clouds and email will be used to exchange pseudo-anonymised data and paperwork between researchers.

Patient and public involvement

Patients were not involved in this study. However, secondary data analysis ensures maximum return from patient involvement in research. The outcome of this IPD-MA will be published in an international peer-reviewed journal. The findings will further be presented at international conferences.

Ethics and dissemination

We will cite the ethics code for each contributing study in the published paper. Contributing studies will be required to remove patient identifiers before providing their data. This includes names, addresses, and date of birth which will be converted to age-at-index-trauma-event and age-at-time-of-assessment. Contributing studies will need to have permission to share anonymised data.

For peer review only

Criteria for included studies

Types of studies

Only randomized studies will be included in this IPD-MA. Articles must be written in English. Unpublished data will be actively sought; hence, non-peer-reviewed studies will also be included. We will perform sensitivity analyses to evaluate the impact of published vs. unpublished studies on our results.

Participants

Studies must have recruited children and adolescents aged 6 to 18 years exposed to a single-event trauma (e.g. road traffic accident) or multi-event trauma (e.g. domestic violence) sufficient to meet the DSM-IV or DSM-5 definitions of a qualifying traumatic event. We will request studies with a broader age range; however, only participants within our defined age range will be included in the IPD. Sensitivity analyses will be conducted if the adult version of a treatment were administered to an adolescent sample. A standardised outcome measure comprising either a diagnostic interview indexing symptom severity or a self-report measure of PTSS must have been administered before and after treatment. Furthermore, a clinically-relevant degree of severity of PTSS at trial baseline must have been present as assessed either

by scoring above a validated cut-off on a PTSS rating scale or by meeting criteria for PTSD. We will also request studies that include both children and adolescents with and without clinically relevant severity of PTSS as defined above. However, again, only those participants with clinically relevant severity of PTSS will be included in the analyses.

Treatments

In line with the NICE guideline[6], we will include studies that used any manualised psychological treatment that we deem to be a trauma-focused cognitive behavioural therapy for children and adolescents. This includes cognitive therapy, cognitive processing therapy (CPT), compassion focused therapy, exposure therapy/prolonged exposure (PE), virtual reality exposure therapy (VRET), imagery rehearsal therapy, and KidNET. We furthermore adopt the NICE guideline description of trauma-focused cognitive behavioural therapy as laid out in the associated paper (Mavranezouli et al.[10], p. 19); namely, "a broad class of psychological interventions that predominantly use trauma-focused cognitive, behavioural or cognitive behavioural techniques and exposure approaches to treatment. Although some interventions place their main emphasis on exposure (e.g. imaginal reliving, producing a written narrative or in vivo exposure) and others on cognitive techniques (e.g. restructuring of trauma-related appraisals), most use a combination." Independent raters will evaluate author descriptions of their treatment with respect to this definition to determine inclusion within the IPD-MA. In contrast to the NICE guideline[6], we will not include mindfulness-based cognitive therapy (MBCT) as a trauma-focused cognitive behavioural therapy.

In addition, treatment may be delivered in-person or online, but must comprise an individual-, rather than group-format, and a multi-session treatment protocol. A minimum of at least one post-treatment / follow-up assessment must have been reported.

For peer review only

Comparison conditions

Trauma-focused cognitive behavioural therapies will be compared: (1) against no intervention (no treatment, waitlist); (2) against TAU; (3) against individual non-trauma focused psychosocial interventions; or (4) against other individual trauma-focused psychosocial interventions. Again, comparison condition type will be determined by two independent raters, based on the author descriptions.

Primary outcomes

The primary outcome variable will be child-reported PTSS using a standardised self-report post-treatment (see paragraph "Strategy for data synthesis" for further information). Proxy-reports (teacher, parent, and caregiver) will be analysed separately. The primary endpoint of post-treatment will be indexed as the assessment completed immediately after completion of trauma-focused cognitive behavioural therapy, less than one month after the final treatment session.

Secondary outcomes

Secondary outcomes will include: a) follow-up assessments of PTSS; b) PTSD diagnoses; and, c) symptoms of comorbid disorders such as depression, anxiety-related, and externalizing problems, reported via self- and proxy-reports. Follow-up lengths to be included comprise assessments between one month and two years following the completion of therapy. During analysis, studies including a follow-up assessment between one and three months post-treatment will be grouped to form a short-term follow-up, and any later assessment points will be grouped per six month period (i.e., 6 months, 12 months). This will result in analysis of

For peer review only

follow-up outcomes in the short-term (1-3 months), and at 6, 12, 18, and 24 months post-treatment.

Search methods for identification of studies and obtaining datasets

Figure 1 depicts our multi-layered search method in order to obtain all potential studies, published and unpublished (in line with The PRISMA-IPD statement[18]).

Please insert Figure 1 here

Figure 1. Flow-chart showing data acquisition.
IPD = individual participant data. NICE = National Institute for Health and Care Excellence.

<u>Electronic searches</u>

Publications identified by the latest NICE guideline for PTSD published in 2018 will be included (the NICE guideline search was completed on the 29th January 2018). To update the results of the NICE search, an electronic search using the same databases will be restricted to publications between the 1st January 2018 and 12th November 2019. We will replicate the NICE guideline search by using the same search terms related to trauma-focused cognitive behavioural therapies. We will exclude specific search terms that are not related to a psychosocial intervention and thereby unlikely to include a cognitive-behavioural therapy approach. This includes physiotherapy and biological interventions (e.g., Transcranial Magnetic Stimulation). We will also exclude specific terms defining any intervention that is not cognitive behaviour therapy-based, e.g., hypnosis or dance therapy. Finally, we will remove terms referring to occupational / return to work support.

Searches in the databases PsycINFO via EBSCOhost, MEDLINE via Ovid, Embase via Ovid, Cochrane Central Register of Controlled Trials (CENTRAL) via Cochrane Library,

For peer review only

CINAHL via EBSCOhost will use the following search plan: (trauma* OR stress*) AND (cognitive therap* OR psychotherap*) AND (trial* OR review*) (see supplementary material for the full search strategy).

No language restrictions will be applied to the search, but only studies published in English will be included. Search results will be deduplicated in Endnote (by IK), then imported into Rayyan (a web-based tool for managing systematic reviews[19]). This will allow for blind screening by raters. Two raters will independently review title and abstract of the records identified in the electronic search. The raters will review the records according to the following exclusion criteria applying the same exclusion order: 1) duplicate; 2) language other than English; 3) review or meta-analysis; 4) no randomized trial; 5) no applicable age range extractable; 6) no manualised trauma-focused cognitive behavioural therapy; 7) group-format; 8) single-session treatment; 9) no assessment post-treatment; 10) no standardized outcome measure to assess PTSS; 11) no clinically relevant PTSS extractable; 12) comparison condition outside protocol. Records deemed ineligible from title and abstract by both raters will be set aside. Records appearing eligible (i.e. not meeting any exclusion criterion) or where eligibility can not be determined due to insufficient information in the abstract will proceed to the full-text stage. Again, at the full-text stage, two raters will examine the remaining records independently. Any disagreements will be resolved via discussion with RMS, MAL, and TD.

For peer review only

Grey literature

Clinical trial registries and archives will be searched up to the 12th November 2019 using the following search string: (child* OR adolesc* OR youth OR young*) AND (PTSD OR posttraumatic stress disorder). We will use the trial registries ClinicalTrials.gov and ISRCTN to identify any relevant unpublished trials, including those that are currently ongoing. Moreover, the archives PsyArXiv and bioRxiv will be searched to identify any relevant

preprints up to 6 months prior (12th of May 2019) of the electronic search (12th of November 2019). Finally, we will check reference lists of included studies and relevant meta-analyses identified by the electronic search to make sure that all available trials will have been detected by the NICE guideline, our electronic search, and grey literature search.

Non-literature based searching

Key authors will be contacted via email to request any unpublished datasets, and Twitter will be used to raise awareness of the IPD-MA.

Data collection

Corresponding authors of eligible studies will be emailed to request data. A reminder email will be sent after two weeks. If an author does not respond after two emails, another author of the study will be contacted as well (either first, second, or last author). A second attempt to contact both authors together will follow. A maximum of three authors per article will be contacted. We will consider study data unavailable if no study authors respond to multiple contact attempts, or if authors indicate that they no longer have access to the data or do not wish to make their data available. A single person for each included study will be designated to whom all queries about the data collection processes and transformation of individual variables will be addressed. When cleaning and preparing a specific data set, communication with the original investigators will take place by email or telephone.

For peer review only

Data extraction, quality checks, and storage

The primary variables to be requested from study investigators are listed in Table 1. We aim to collect data on all of these variables from all studies, regardless of whether such data were previously published. For all outcomes, unimputed and untransformed data will be requested. Data will be cleaned and stored separately for each study. Spot checks will be completed to ensure data quality. The pattern of treatment allocation for each included study will be checked to ensure that randomisation and allocation sequence appear appropriate, in accordance with guidelines recommended by Tierney et al.[20]. For final checks before analysis and the statistical analyses, the datasets will be combined into a single dataset. Data will be stored in password-protected files on an encrypted University of Cambridge server.

Table 1

Individual participant data to be extracted from included studies

**Treatment-related factors**	**Child-related factors**	**Outcomes**
Descriptives	*Demographics*	
Trial identifier	Anonymised participant identifier	
Country of completion	Gender	
Information about risk of bias	Age	
Type of trauma-focused cognitive behavioural therapy	Ethnicity	
Type of comparison group/s	Trauma type of index-event	
Number of sessions	Trauma history	
Length of treatment in weeks		
Involvement of caregivers		
Any potential covariates (e.g., mode of administration, profession of therapists)	Any potential covariates (e.g., pre-treatment levels of dysfunctional posttraumatic cognitions, IQ, social support, treatment expectancy, therapeutic alliance)	Any related outcome variables (e.g., post-treatment level of dysfunctional posttraumatic cognitions and changes in coping behaviours)

For peer review only

	Psychological symptoms pre-treatment	*Psychological symptoms post-treatment and follow-up*
	Pre-treatment self- and proxy-reported PTSS	Self- and proxy-reported PTSS post-treatment and follow-up
	Pre-treatment self- and proxy-reported depression symptoms	Self- and proxy-reported depression symptoms post-treatment and follow-up
	Pre-treatment self- and proxy-reported anxiety-related symptoms	Self- and proxy-reported anxiety-related symptoms post-treatment and follow-up
	Pre-treatment self- and proxy-reported externalizing problems	Self- and proxy-reported externalizing problems post-treatment and follow-up
	Diagnoses pre-treatment	*Diagnoses post-treatment and follow-up*
	Pre-treatment diagnostic status of PTSD	Diagnostic status of PTSD post-treatment and follow-up
	Pre-treatment diagnostic status of comorbid disorders	Diagnostic status of comorbid disorders post-treatment and follow-up
	Reason for missing data	Reason for missing data

Note. PTSS = posttraumatic stress symptoms. PTSD = posttraumatic stress disorder.

For peer review only

Risk of bias

Two raters will independently evaluate the risk of bias for the included studies by using the revised Cochrane Risk of Bias Tool (RoB 2[21]) to access study quality and risk of bias due to the randomisation process, deviations from intended interventions, missing outcome data, measurement of the outcome, and selection of the reported result. Each study will be rated as of high risk, some concerns, or low risk.

Strategy for data synthesis

Data will be analysed across a series of stages, in order to be guided by data availability and the degree of potential for harmonisation. Unimputed and untransformed data will be requested.

Firstly, for published studies, key variables will be re-analysed within each study (e.g., participant numbers per treatment condition, mean PTSS scores pre- and post-treatment, numbers gender, mean age), as to identify any potential inconsistencies in the supplied data.

Secondly, data will be harmonised as far as possible: (a) the definitions and scales of outcomes (e.g., standardizing PTSS total scores across different measures); (b) the timings of measurements (e.g., pre-treatment defined as the assessment directly before start of treatment; post-treatment defined as the assessment immediately after completing treatment, less than one month after the final treatment session); (c) the definitions, scales and/or subgroups used for covariates (e.g., the specific index trauma event will be grouped into accidental trauma, natural disaster, war trauma, or interpersonal trauma).

Thirdly, depending on the amount of missing data and whether missing at random assumptions are met, multiple imputation will be carried out.

Based on the step-wise approach described before, decisions may be made e.g. to put aside certain desired analyses or adjustment factors if it is felt that data are too limited and may bias results. Once the final constitution of the model has been agreed based on the above, we will proceed to the meta-analysis pooling itself. Note that modelling will be done for each outcome (as specified in Table 1) separately. All analyses will be completed using random-effects models employing restricted maximum likelihood estimation. However, if there is considerable heterogeneity in the quality of studies (indexed by RoB 2), a sensitivity analysis will be completed comparing random-effects and fixed-effects models.

For peer review only

Depending on the IPD data sets we receive, we will collect aggregate data (AD) from studies where IPD could not be obtained and combine it with the IPD to tackle inclusion bias[22]. In this case, sensitivity analyses will be performed comparing an IPD-only meta-analysis with a meta-analysis that combines IPD and AD.

One-stage approaches will be applied using the R software[23]. We will investigate the overall summary of treatment effect and we are further interested in the heterogeneity in treatment effect across- and within-studies. If the one-stage model fails to converge, a two-stage model will be calculated.

Hypothesis 1

A one-stage linear mixed effects (LME) model with random intercept and outcome baseline adjustment with different residual variance per study will be applied to analyse the effect of trauma-focused cognitive behavioural therapies on the continuous outcome of PTSS post-treatment. A mixed effects logistic regression model will be used to analyse the treatment effect on the binary outcome PTSD diagnosis post-treatment. Sensitivity analysis will be completed contrasting a (single) random intercept (as primary) with a separate fixed intercept for each study (as sensitivity). As described before, if the one-stage model fails to converge, a two-stage model will be completed.

Hypothesis 2a

We will use meta-regression to explore whether the overall effect of trauma-focused cognitive behavioural therapies varies in relation to treatment-related factors such as pre-defined intended length of treatment and pre-defined intended involvement of caregivers.

For peer review only

Hypothesis 2b

To investigate whether the overall effect of trauma-focused cognitive behavioural therapies varies by child-related factors such as age, gender, trauma type of index-event, trauma-history, and symptom severity pre-treatment, subject-level interactions will be investigated. Interaction term between treatment status and subject-level covariates will be specified[22].

Random-effects distributions for the interaction effects will be specified. Effects on the continuous primary outcome of PTSS post-treatment will be analysed using a one-stage LME model with random intercept to account for correlation between the interaction estimate and other parameter estimates. Patient-level covariates will be centred to separate within-trial and across-trial effects. A mixed effects logistic regression model for the binary secondary outcome of PTSD diagnosis post-treatment will be applied. Sensitivity analysis will be completed contrasting a (single) random intercept (as primary) with a separate fixed intercept for each study (as sensitivity). As described before, if the one-stage model fails to converge, a two-stage model will be completed.

Additional analyses with sub-samples

Depending on the data provided, we will investigate further treatment-related factors such as pre-defined mode of administration, profession of therapists, and treatment expectancy pre-treatment. Moreover, additional child-related factors such as comorbidity pre-treatment, pre-treatment levels of dysfunctional posttraumatic cognitions, IQ, and pre-treatment parental mental health will be addressed.

For peer review only

Future analyses

In future investigations of the obtained data, we plan to conduct mediation analyses within the IPD-MA context to evaluate mechanisms of action of trauma-focused cognitive behavioural therapies. The most promising candidates seem to be changes in targeted cognitive and behavioural processes; for example, improvements in dysfunctional posttraumatic cognitions of the child regarding being permanently and disturbingly changed, or feeling vulnerable[24-26], as well as changes in safety-seeking behaviours[25].

Moreover, we are aiming to investigate non-responding, deterioration, and possible predictors for dropping out from trauma-focused cognitive behavioural therapies across studies.

For peer review only

AUTHORS' CONTRIBUTIONS

AdH, CH, RMS, MAL, and TD designed the project. IK, MB, KK, SDP, and DJF contributed to study methods. All authors critically reviewed and approved the final manuscript.

COMPETING INTERESTS STATEMENT

The authors declare that they have no competing interests.

FUNDING STATEMENT

Anke de Haan is funded by the Swiss National Science Foundation (Grant Reference: P2ZHP1_187612). Caitlin Hitchcock and Shivam D. Patel are partly supported by the Economic and Social Research Council (Grant Reference: ES/R010781/1). Melissa Black is partly supported by the National Institute for Health Research Cambridge Biomedical Research Centre. Kristel Klaus is funded by the UK Medical Research Council (Grant Reference: MC_PC_17213). David J. Fisher is funded by the UK Medical Research Council (Grant Reference: MC_UU_12023/24). Tim Dalgleish is funded by the UK Medical Research Council (Grant Reference: SUAG/043 G101400) and partly supported by the National Institute for Health Research Cambridge Biomedical Research Centre.

For peer review only

REFERENCES

1. Forbes D, Creamer M, Bisson JI, et al. A guide to guidelines for the treatment of PTSD and related conditions. *J Trauma Stress* 2010;23(5):537-52. doi: 10.1002/jts.20565
2. Cohen JA, Mannarino AP, Deblinger E. Treating trauma and traumatic grief in children and adolescents. New York, NY, US: Guilford Press, 2006.
3. Smith P, Perrin S, Yule W, et al. Post traumatic stress disorder: cognitive therapy with children and young people. London: Routledge 2010.
4. Foa EB, Chrestman K, Gilboa-Schechtman E. Prolonged exposure manual for children and adolescents suffering from PTSD. New York, NY, US: Oxford University Press 2008.
5. Schauer M, Neuner F, Elbert T. Narrative Exposure Therapy for Children and Adolescents (KIDNET). In: Landolt MA, Cloitre M, Schnyder U, eds. Evidence-based treatments for trauma related disorders in children and adolescents. Cham: Springer International Publishing 2017:227-50.
6. National Institute for Health and Care Excellence. Post-traumatic stress disorder NICE guideline [NG116]. 2018. https://www.nice.org.uk/guidance/ng116/evidence (accessed 10.2019).
7. Dorsey S, McLaughlin KA, Kerns SEU, et al. Evidence base update for psychosocial treatments for children and adolescents exposed to traumatic events. *J Clin Child Adolesc Psychol* 2017;46(3):303-30. doi: 10.1080/15374416.2016.1220309
8. Gutermann J, Schreiber F, Matulis S, et al. Psychological treatments for symptoms of posttraumatic stress disorder in children, adolescents, and young adults: a meta-analysis. *Clin Child Fam Psychol Rev* 2016;19:77–93. doi: 10.1007/s10567-016-0202-5
9. Lenz AS, Hollenbaugh KM. Meta-analysis of trauma-focused cognitive behavioral therapy for treating PTSD and co-occurring depression among children and adolescents.

For peer review only

Counseling Outcome Research and Evaluation 2015;6(1):18-32. doi: 10.1177/2150137815573790

10. Mavranezouli I, Megnin-Viggars O, Daly C, et al. Psychological and psychosocial treatments for children and young people with post-traumatic stress disorder: a network meta-analysis. *J Child Psychol Psychiatry* 2020;61(1):18-29. doi: 10.1111/jcpp.13094
11. Morina N, Koerssen R, Pollet TV. Interventions for children and adolescents with posttraumatic stress disorder: a meta-analysis of comparative outcome studies. *Clin Psychol Rev* 2016;47:41-54. doi: 10.1016/j.cpr.2016.05.006
12. Deblinger E, Mannarino AP, Cohen JA, et al. Trauma-focused cognitive behavioral therapy for children: impact of the trauma narrative and treatment length. *Depress Anxiety* 2011;28(1):67-75. doi: 10.1002/da.20744
13. Goldbeck L, Muche R, Sachser C, et al. Effectiveness of trauma-focused cognitive behavioral therapy for children and adolescents: a randomized controlled trial in eight German mental health clinics. *Psychother Psychosom* 2016;85(3):159-70.
14. Knutsen M, Jensen TK. Changes in the trauma narratives of youth receiving trauma-focused cognitive behavioral therapy in relation to posttraumatic stress symptoms. *Psychother Res* 2019;29(1):99-111. doi: 10.1080/10503307.2017.1303208
15. Ormhaug SM, Jensen TK, Wentzel-Larsen T, et al. The therapeutic alliance in treatment of traumatized youths: relation to outcome in a randomized clinical trial. *J Consult Clin Psychol* 2014;82(1):52-64. doi: 10.1037/a0033884
16. Ready CB, Hayes AM, Yasinski CW, et al. Overgeneralized beliefs, accommodation, and treatment outcome in youth receiving trauma-focused cognitive behavioral therapy for childhood trauma. *Behav Ther* 2015;46(5):671-88. doi: 10.1016/j.beth.2015.03.004
17. Yasinski C, Hayes AM, Ready CB, et al. In-session caregiver behavior predicts symptom change in youth receiving trauma-focused cognitive behavioral therapy (TF-CBT). *J Consult Clin Psychol* 2016;84(12):1066-77. doi: 10.1037/ccp0000147

For peer review only

18. Stewart LA, Clarke M, Rovers M, et al. Preferred reporting items for systematic review and meta-analyses of individual participant data: the PRISMA-IPD statement. *JAMA* 2015;313(16):1657-65. doi: 10.1001/jama.2015.3656

19. Ouzzani M, Hammady H, Fedorowicz Z, et al. Rayyan - a web and mobile app for systematic reviews. *Syst Rev* 2016;5(1):210. doi: 10.1186/s13643-016-0384-4

20. Tierney JF, Vale C, Riley R, et al. Individual participant data (IPD) meta-analyses of randomised controlled trials: guidance on their use. *PLoS Med* 2015;12(7):e1001855. doi: 10.1371/journal.pmed.1001855

21. Sterne JAC, Savović J, Page MJ, et al. RoB 2: a revised tool for assessing risk of bias in randomised trials. *BMJ* 2019;366:l4898. doi: 10.1136/bmj.l4898

22. Debray TP, Moons KG, van Valkenhoef G, et al. Get real in individual participant data (IPD) meta-analysis: a review of the methodology. *Res Synth Methods* 2015;6(4):293-309. doi: 10.1002/jrsm.1160

23. R Core Team. R: a language and environment for statistical computing. Vienna: R Foundation for Statistical Computing 2020.

24. Jensen TK, Holt T, Mørup Ormhaug S, et al. Change in post-traumatic cognitions mediates treatment effects for traumatized youth - a randomized controlled trial. *J Couns Psychol* 2018;65(2):166-77. doi: 10.1037/cou0000258

25. Meiser-Stedman R, Smith P, McKinnon A, et al. Cognitive therapy as an early treatment for post-traumatic stress disorder in children and adolescents: a randomized controlled trial addressing preliminary efficacy and mechanisms of action. *J Child Psychol Psychiatry* 2017;58(5):623-33. doi: 10.1111/jcpp.12673

26. Pfeiffer E, Sachser C, de Haan A, et al. Dysfunctional posttraumatic cognitions as a mediator of symptom reduction in trauma-focused cognitive behavioral therapy with children and adolescents: results of a randomized controlled trial. *Behav Res Ther* 2017;97:178-82. doi: 10.1016/j.brat.2017.08.001

For peer review only

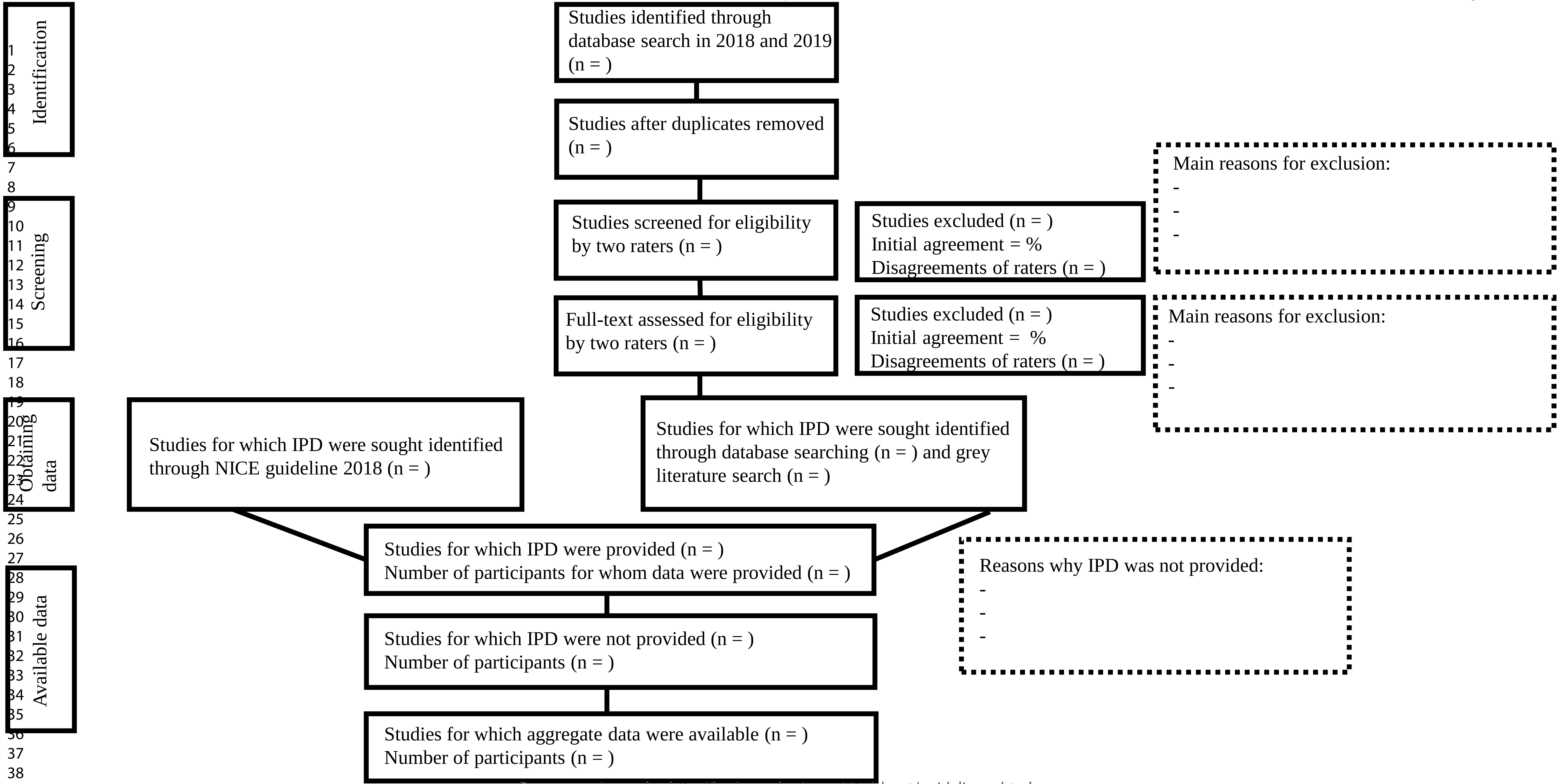
Identification
Studies identified through database search in 2018 and 2019 (n =)
Studies after duplicates removed (n =)
Screening
Studies screened for eligibility by two raters (n =)
Studies excluded (n =)
Initial agreement = %
Disagreements of raters (n =)
Main reasons for exclusion:
-
-
-
Full-text assessed for eligibility by two raters (n =)
Studies excluded (n =)
Initial agreement = %
Disagreements of raters (n =)
Main reasons for exclusion:
-
-
-
Obtaining data
Studies for which IPD were sought identified through NICE guideline 2018 (n =)
Studies for which IPD were sought identified through database searching (n =) and grey literature search (n =)
Available data
Studies for which IPD were provided (n =)
Number of participants for whom data were provided (n =)
Reasons why IPD was not provided:
-
-
-
Studies for which IPD were not provided (n =)
Number of participants (n =)
Studies for which aggregate data were available (n =)
Number of participants (n =)

Search strategy de Haan et al.

Database	Number of hits
Medline	1913
Embase	2000
CINAHL	2506
Cochrane	2413
PsycINFO	2105
Total	10937
Total deduplicated	7091

Searches run 12th November 2019

Limited by publication date 2018 to date

Medline

Database: Ovid MEDLINE(R) and Epub Ahead of Print, In-Process & Other Non-Indexed Citations, Daily and Versions(R) <1946 to November 08, 2019>

Search Strategy:

----

1 stress disorders, traumatic/ or combat disorders/ or psychological trauma/ or stress disorders, post-traumatic/ or stress disorders, traumatic, acute/ or stress, psychological/ (147421)

2 (railway spine or (rape adj2 trauma*) or reexperienc* or re experienc* or torture syndrome or traumatic neuros* or traumatic stress).ti,ab. (15096)

3 (trauma* and (avoidance or grief or horror or death* or nightmare* or night mare* or emotion*)).ti,ab. (35178)

4 (posttraumatic* or post traumatic* or stress disorder* or acute stress or ptsd or asd or desnos or (combat neuros* or combat syndrome or concentration camp syndrome or extreme stress or flashback* or flash back* or hypervigilan* or hypervigilen* or psych* stress or psych* trauma* or psycho?trauma* or psychotrauma*) or (posttrauma* or traumagenic* or traumatic stress*)).ti,ab. (106800)

5 or/1-4 (245567)

6 psychotherapy/ (53279)

7 (((psycholog* or psycho social* or psychosocial*) adj3 (intervention* or program* or therap* or treat*)) or psychotherap* or psycho therap* or talk* therap* or therapeutic technique* or therapist* or third wave or time limited).ti,ab,sh. (144180)

8 exp behavior therapy/ (72171)

9 (((behaviour* or behavior*) adj2 cognitiv*) or cbt or ccbt or ((behav* or cognitive*) adj3 (intervention* or manag* or program* or restructure* or therap* or treat*)) or (stress inoculation adj2 (intervention* or program* or therap* or train* or treat*)) or (behav* adj2 activat*) or ((trauma adj (based or focused or led)) or exposure based or prolonged exposure)).ti,ab. (115271)

10 emotions/ (63893)

For peer review only

11 (((compassion or emotion* or emotive*) adj (based or focused or led)) or emotional processing or ((compassion or emotion* or emotive*) adj3 (coach* or intervention* orprogram* or therap* or treat*))).ti,ab. (7907)

12 implosive therapy/ or narrative therapy/ or virtual reality exposure therapy/ (1886)

13 (((augmented or virtual) adj2 reality) or (virtual adj (environment or restorative)) or ((exposure or implosive or virtual reality) adj2 (intervention* or program* or therap* or train*))).ti,ab. (16289)

14 ((imagery adj2 (rehears* or re hears*)) or (((lower* or reduc*) adj3 (bad dream* or nightmare*)) and (intervention* or program* or therap* or treat*)) or ((intervention* or program* or therap* or treat*) adj3 nightmare*)).mp. or ((presleep or presleep) adj2 imagery).ti,ab. (267)

15 (mindfulness or ((exposure or narrative) adj therapy)).sh. (2743)

16 (kidnet or mindful* or narrative therap*).ti,ab. (8835)

17 debrief*.ti,ab. (3803)

18 eye movement desensitization reprocessing/ (205)

19 (emdr or (eye movement adj2 desensiti*)).ti,ab. (671)

20 psychotherapy, psychodynamic/ (529)

21 (psychodynamic or (dynamic adj (psychotherapy* or therap*)) or incident reduction or ((brain or transcranial) adj2 stimulat*) or rtms).ti,ab. (36027)

22 (psychoanal* or psychosomatic*).ti,ab. (26156)

23 exp counseling/ (42669)

24 counsel*.ti,ab. (103170)

25 (hg therap* or human givens).ti,ab. (14)

26 couples therapy/ or family therapy/ or marital therapy/ or exp parents/ed (22720)

27 (((con?joint or couple* or family or families or husband* or marriage* or marital* or partner* or relations* or spous* or wife or wives* or (child* adj5 parent*)) adj6 (counsel* or intervention* or program* or support* or therap* or treat*)) or ((couples* or family* or relations*) adj (based or focused or led)) or ecological therap* or expressed emotion or family dynamics or family relationships).tw. (171018)

28 ((child* adj2 family traumatic stress intervention) or cftsi).ti,ab. (2)

29 psychoeducation.sh. or (psychoed* or psycho ed*).ti,ab. (5975)

30 community networks/ or friends/ or exp peer group/ or self care/ or self-help groups/ or social networking/ or social support/ (133084)

31 ((self adj (administer* or assess* or attribut* or care or change or directed or efficacy or help* or guide* or instruct* or manag* or medicat* or monitor* or regulat* or reinforc* or re inforc* or support* or technique* or therap* or train* or treat*)) or selfadminister* or selfassess* or selfattribut* or selfcare or selfchange or selfdirected or selfefficacy or selfhelp* or selfguide* or selfinstruct* or selfmanag* or selfmedicat* or selfmonitor* or selfregulat* or selfreinforc* or self re inforc* or selfsupport* or selftechnique* or selftherap* or selftrain* or selftreat* or (wellness adj (therap* or train* or treat*))).ti,ab,sh. (139336)

32 (befriend* or be*1 friend* or buddy or buddies or ((community or lay or paid or support) adj (person or worker*))).ti,ab. (3080)

33 (((consumer* or famil* or friend* or lay or mutual* or peer* or social* or spous* or voluntary or volunteer*) adj3 (assist* or advice* or advis* or counsel* or educat* or forum* or help* or mentor* or network* or support* or visit*)) or ((consumer* or famil* or peer* or self help or social* or support* or voluntary or volunteer*) adj2 group*) or ((consumer* or famil* or friend* or lay or mutual* or peer* or self

For peer review only

help or social* or spous* or support* or voluntary or volunteer*) adj3 (intervention* or program* or rehab* or therap* or service* or skill* or treat*)) or (((consumer* or famil* or friend* or lay* or peer* or spous* or user* or support* or voluntary or volunteer*) adj (based or counsel* or deliver* or interact* or led or mediat* or operated or provides or provider* or run*)) or ((consumer* or famil* or friend* or lay* or peer* or relation* or spous* or support*) adj3 trust*) or voluntary work*)).ti,ab. (314043)

34 (((lay or peer*) adj3 (advis* or consultant or educator* or expert* or facilitator* or instructor* or leader* or mentor* or person* or tutor* or worker*)) or expert patient* or mutual aid).ti,ab. (6617)

35 (peer* adj3 (assist* or counsel* or educat* or program* or rehab* or service* or supervis*)).ti,ab. (5735)

36 ((psychoeducat* or psycho educat*) adj3 (group or network* or service*)).ti,ab. (774)

37 ((psychosocial or social) adj work*).ti,ab. (15927)

38 ((ptsd or posttrauma* or post trauma* or trauma*) adj2 support*).ti,ab. (1565)

39 recovery support.ti,ab. (173)

40 assisted living facilities/ or emergency shelter/ or group homes/ or halfway houses/ or housing/ or independent living/ or residential facilities/ (30265)

41 ((resident* or hous* or accommod* or commun* or comu* or home*) adj5 (support* or support* or shelter* or outreach* or visit* or appointment*)).ti,ab. (50036)

42 (residential treatm* or residential facility* or supported hous* or public hous*).ti,ab. (3249)

43 (accomod* or assertive community treatment* or home* or housing* or outreach* or residential*).ti,ab. (525321)

44 placement.ti,ab. (122669)

45 or/6-44 (1653237)

46 meta analysis.sh,pt. or "meta-analysis as topic"/ or "review literature as topic"/ (129841)

47 (exp databases, bibliographic/ or (((electronic or computer* or online) adj database*) or bids or cochrane or embase or index medicus or isi citation or medline or psyclit or psychlit or scisearch or science citation or (web adj2 science)).ti,ab.) and (review*.ti,ab,sh,pt. or systematic*.ti,ab.) (163499)

48 ((analy* or assessment* or evidence* or methodol* or quantativ* or systematic*) adj2 (overview* or review*)).tw. or ((analy* or assessment* or evidence* or methodol* or quantativ* or systematic*).ti. and review*.ti,pt.) or (systematic* adj2 search*).ti,ab. (329117)

49 (metaanal* or meta anal* or (research adj (review* or integration))).ti,ab. or reference list*.ab. or bibliograph*.ab. or published studies.ab. or relevant journals.ab. or selection criteria.ab. or (data adj (extraction or synthesis)).ab. or (handsearch* or ((hand or manual) adj search*)).ti,ab. or (mantel haenszel or peto or dersimonian or der simonian).ti,ab. or (fixed effect* or random effect*).ti,ab. or ((pool* or combined or combining) adj2 (data or trials or studies or results)).ti,ab. (300023)

50 or/46-49 (562928)

51 exp clinical trial/ or exp "clinical trials as topic"/ or cross-over studies/ or double-blind method/ or placebos/ or random allocation/ or single-blind method/ (1194973)

52 ((clinical adj2 trial*) or (crossover or cross over)).ti,ab. (429760)

53 (((single* or doubl* or trebl* or tripl*) adj2 blind*) or mask* or dummy or doubleblind* or singleblind* or trebleblind* or tripleblind*).ti,ab. (240549)

54 (placebo* or random*).ti,ab. (1162532)

55 or/51-54 (2043304)

For peer review only

56 5 and 45 and (50 or 55) (12008)

57 animals/ not human*.mp. (4342443)

58 56 not 57 (11757)

59 limit 58 to yr="2018 -Current" (1913)

****

Embase

Database: Embase <1974 to 2019 November 08>

Search Strategy:

----

1 *acute stress/ or *behavioural stress/ or *emotional stress/ or *critical incident stress/ or *mental stress/ or *posttraumatic stress disorder/ or *psychotrauma/ (69082)

2 (trauma* and (avoidance or grief or horror or death* or nightmare* or night mare* or emotion*)).ti,ab. (50168)

3 (railway spine or (rape adj2 trauma*) or reexperienc* or re experienc* or torture syndrome or traumatic neuros* or traumatic stress).ti,ab. (20306)

4 (posttraumatic* or post traumatic* or stress disorder* or acute stress or ptsd or asd or desnos or (combat neuros* or combat syndrome or concentration camp syndrome or extreme stress or flashback* or flash back* or hypervigilan* or hypervigilen* or psych* stress or psych* trauma* or psycho?trauma* or psychotrauma*) or (posttrauma* or traumagenic* or traumatic stress*)).ti,ab. (139168)

5 or/1-4 (212899)

6 *psychotherapy/ (38266)

7 exp *behavior therapy/ or exp *cognitive therapy/ (29422)

8 (((psycholog* or psycho social* or psychosocial*) adj3 (intervention* or program* or therap* or treat*)) or psychotherap* or psycho therap* or talk* therap* or therapeutic technique* or therapist* or third wave or time limited).ti,ab,sh. (246754)

9 (((behaviour* or behavior*) adj2 cognitiv*) or cbt or ccbt or ((behav* or cognitive*) adj3 (intervention* or manag* or program* or restructure* or therap* or treat*)) or (stress inoculation adj2 (intervention* or program* or therap* or train* or treat*)) or (behav* adj2 activat*) or ((trauma adj (based or focused or led)) or exposure based or prolonged exposure)).ti,ab. (158532)

10 *emotion/ (28919)

11 (((compassion or emotion* or emotive*) adj (based or focused or led)) or emotional processing or ((compassion or emotion* or emotive*) adj3 (coach* or intervention* orprogram* or therap* or treat*))).ti,ab. (10888)

12 exposure therapy/ or narrative therapy/ or virtual reality exposure therapy/ (1602)

13 (((augmented or virtual) adj2 reality) or (virtual adj (environment or restorative)) or ((exposure or implosive or virtual reality) adj2 (intervention* or program* or therap* or train*))).ti,ab. (21776)

For peer review only

14 ((imagery adj2 (rehears* or re hears*)) or (((lower* or reduc*) adj3 (bad dream* or nightmare*)) and (intervention* or program* or therap* or treat*)) or ((intervention* or program* or therap* or treat*) adj3 nightmare*)).mp. or ((presleep or presleep) adj2 imagery).ti,ab. (454)

15 (mindfulness or ((exposure or narrative) adj therapy)).sh. (23980)

16 (kidnet or mindful* or narrative therap*).ti,ab. (12154)

17 debrief*.ti,ab. (6509)

18 (emdr or (eye movement adj2 desensiti*)).ti,ab. (925)

19 psychodynamic psychotherapy/ (1214)

20 (psychodynamic or (dynamic adj (psychotherapy* or therap*)) or incident reduction or ((brain or transcranial) adj2 stimulat*) or rtms).ti,ab. (53209)

21 (psychoanal* or psychosomatic*).ti,ab. (35159)

22 exp counseling/ (159666)

23 counsel*.ti,ab. (148163)

24 (hg therap* or human givens).ti,ab. (21)

25 couple therapy/ or family therapy/ or marital therapy/ or exp parent/ (246712)

26 (((con?joint or couple* or family or families or husband* or marriage* or marital* or partner* or relations* or spous* or wife or wives* or (child* adj5 parent*)) adj6 (counsel* or intervention* or program* or support* or therap* or treat*)) or ((couples* or family* or relations*) adj (based or focused or led)) or ecological therap* or expressed emotion or family dynamics or family relationships).tw. (226599)

27 ((child* adj2 family traumatic stress intervention) or cftsi).ti,ab. (6)

28 psychoeducation.sh. or (psychoed* or psycho ed*).ti,ab. (12914)

29 friendship/ or peer counseling/ or peer group/ or self help/ or self care/ or social network/ or social support/ or support group/ (193307)

30 ((self adj (administer* or assess* or attribut* or care or change or directed or efficacy or help* or guide* or instruct* or manag* or medicat* or monitor* or regulat* or reinforc* or re inforc* or support* or technique* or therap* or train* or treat*)) or selfadminister* or selfassess* or selfattribut* or selfcare or selfchange or selfdirected or selfefficacy or selfhelp* or selfguide* or selfinstruct* or selfmanag* or selfmedicat* or selfmonitor* or selfregulat* or selfreinforc* or self re inforc* or selfsupport* or selftechnique* or selftherap* or selftrain* or selftreat* or (wellness adj (therap* or train* or treat*))).ti,ab,sh. (188037)

31 (befriend* or be*1 friend* or buddy or buddies or ((community or lay or paid or support) adj (person or worker*))).ti,ab. (4130)

32 (((consumer* or famil* or friend* or lay or mutual* or peer* or social* or spous* or voluntary or volunteer*) adj3 (assist* or advice* or advis* or counsel* or educat* or forum* or help* or mentor* or network* or support* or visit*)) or ((consumer* or famil* or peer* or self help or social* or support* or voluntary or volunteer*) adj2 group*) or ((consumer* or famil* or friend* or lay or mutual* or peer* or self help or social* or spous* or support* or voluntary or volunteer*) adj3 (intervention* or program* or rehab* or therap* or service* or skill* or treat*)) or (((consumer* or famil* or friend* or lay* or peer* or spous* or user* or support* or voluntary or volunteer*) adj (based or counsel* or deliver* or interact* or led or mediat* or operated or provides or provider* or run*)) or ((consumer* or famil* or friend* or lay* or peer* or relation* or spous* or support*) adj3 trust*) or voluntary work*)).ti,ab. (410161)

33 (((lay or peer*) adj3 (advis* or consultant or educator* or expert* or facilitator* or instructor* or leader* or mentor* or person* or tutor* or worker*)) or expert patient* or mutual aid).ti,ab. (8679)

34 (peer* adj3 (assist* or counsel* or educat* or program* or rehab* or service* or supervis*)).ti,ab. (7548)

For peer review only

35 ((psychoeducat* or psycho educat*) adj3 (group or network* or service*)).ti,ab. (1226)

36 ((psychosocial or social) adj work*).ti,ab. (22915)

37 ((ptsd or posttrauma* or post trauma* or trauma*) adj2 support*).ti,ab. (1890)

38 recovery support.ti,ab. (216)

39 assisted living facility/ or emergency shelter/ or halfway house/ or housing/ or independent living/ or residential home/ or residential home/ (36467)

40 ((resident* or hous* or accommod* or commun* or comu* or home*) adj5 (support* or support* or shelter* or outreach* or visit* or appointment*)).ti,ab. (66837)

41 (residential treatm* or residential facility* or supported hous* or public hous*).ti,ab. (3836)

42 (accomod* or assertive community treatment* or home* or housing* or outreach* or residential*).ti,ab. (693104)

43 placement.ti,ab. (174618)

44 or/6-43 (2352266)

45 meta analysis/ or "meta analysis (topic)"/ or systematic review/ (349285)

46 (exp bibliographic database/ or (((electronic or computer* or online) adj database*) or bids or cochrane or embase or index medicus or isi citation or medline or psyclit or psychlit orscisearch or science citation or (web adj2 science)).ti,ab.) and (review*.ti,ab,sh,pt. or systematic*.ti,ab.) (223431)

47 ((analy* or assessment* or evidence* or methodol* or quantativ* or systematic*) adj2 (overview* or review*)).tw. or ((analy* or assessment* or evidence* or methodol* or quantativ* or systematic*).ti. and review*.ti,pt.) or (systematic* adj2 search*).ti,ab. (392909)

48 (metaanal* or meta anal* or (research adj (review* or integration))).ti,ab. or reference list*.ab. or bibliograph*.ab. or published studies.ab. or relevant journals.ab. or selection criteria.ab. or (data adj (extraction or synthesis)).ab. or (handsearch* or ((hand or manual) adj search*)).ti,ab. or (mantel haenszel or peto or dersimonian or der simonian).ti,ab. or (fixed effect* or random effect*).ti,ab. or ((pool* or combined or combining) adj2 (data or trials or studies or results)).ti,ab. (393628)

49 or/45-48 (775067)

50 exp "clinical trial (topic)"/ or exp clinical trial/ or crossover procedure/ or double blind procedure/ or placebo/ or randomization/ or random sample/ or single blind procedure/ (1962363)

51 (clinical adj2 trial*).ti,ab. (504366)

52 (crossover or cross over).ti,ab. (101345)

53 (((single* or doubl* or trebl* or tripl*) adj2 blind*) or mask* or dummy or doubleblind* or singleblind* or trebleblind* or tripleblind*).ti,ab. (323907)

54 (placebo* or random*).ti,ab. (1578946)

55 or/50-54 (2965134)

56 5 and 44 and (49 or 55) (10225)

57 animals/ not human*.mp. (857762)

58 56 not 57 (10209)

59 limit 58 to yr="2018 -Current" (2000)

For peer review only

****

PsycINFO

#	Query	Results
S64	s59 not s60 Limiters - Publication Year: 2018-2020	2,105
S63		277,708
S62		1,761,949
S61	s59 not s60	16,053
S60	animal not human	289,401
S59	S5 AND S48 AND S58	16,256
S58	S56 OR S57	765,475
S57	((clinical trials or placebo or random sampling)) OR (clinical adj2 trial*) OR (crossover or cross over) OR ((((single* or doubl* or trebl* or tripl*) n2 blind*) or mask* or dummy or doubleblind* or singleblind* or trebleblind* or tripleblind*)) OR (placebo* or random*) OR treatment outcome*	347,654
S56	S49 OR S50 OR S51 OR S52 OR S53 OR S54 OR S55	468,514
S55	(metaanal* or meta anal* or (research n1 (review* or integration))) OR (reference list* or bibliograph* or published studies or relevant journals or selection criteria) OR ((data n1 (extraction or synthesis))) OR ((handsearch* or ((hand or manual) n1 search*))) OR ((mantel haenszel or peto or dersimonian or der simonian)) OR ((fixed effect* or random effect*)) OR (((pool* or combined or combining) n2 (data or trials or studies or results)))	142,371
S54	(systematic* n2 search*)	6,258
S53	(analy* or assessment* or evidence* or methodol* or quantativ* or systematic*) AND review*	297,715
S52	((analy* or assessment* or evidence* or methodol* or quantativ* or systematic*) n2 (overview* or review*))	317,006
S51	(((electronic or computer* or online) n1 database*) or bids or cochrane or embase or index medicus or isi citation or medline or psyclit or psychlit or scisearch or science citation or (web n2 science)) AND (review* or systematic*)	53,766
S50	computer searching	1,605

S49	((literature review or meta analysis)) OR systematic review	138,174
S48	S6 OR S7 OR S8 OR S9 OR S10 OR S11 OR S12 OR S13 OR S14 OR S15 OR S16 OR S17 OR S18 OR S19 OR S20 OR S21 OR S22 OR S23 OR S24 OR S25 OR S26 OR S27 OR S28 OR S29 OR S30 OR S31 OR S32 OR S33 OR S34 OR S35 OR S36 OR S37 OR S38 OR S39 OR S40 OR S41 OR S42 OR S43 OR S44 OR S45 OR S46 OR S47	1,682,227
S47	TI (placement or student placement) OR AB placement	26,717
S46	TI ((accomod* or assertive community treatment* or home* or housing* or outreach* or residential*)) OR AB ((accomod* or assertive community treatment* or home* or housing* or outreach* or residential*))	190,252
S45	TI ((residential treatm* or residential facility* or supported hous* or public hous*)) OR AB ((residential treatm* or residential facility* or supported hous* or public hous*))	9,115
S44	TI (((resident* or hous* or accommod* or commun* or comu* or home*) n5 (support* or support* or shelter* or outreach* or visit* or appointment*))) OR AB (((resident* or hous* or accommod* or commun* or comu* or home*) n5 (support* or support* or shelter* or outreach* or visit* or appointment*)))	39,683
S43	TI (((resident* or hous* or accommod* or commun* or comu* or home*) adj5 (support* or support* or shelter* or outreach* or visit* or appointment*))) OR AB (((resident* or hous* or accommod* or commun* or comu* or home*) adj5 (support* or support* or shelter* or outreach* or visit* or appointment*)))	0
S42	(((((DE "Assisted Living") OR (DE "Shelters")) OR (DE "Group Homes")) OR (DE "Halfway Houses")) OR (DE "Housing")) OR (DE "Residential Care Institutions")	19,622
S41	TI recovery support OR AB recovery support	2,144
S40	TI (((ptsd or posttrauma* or post trauma* or trauma*) n2 support*)) OR AB (((ptsd or posttrauma* or post trauma* or trauma*) n2 support*))	1,570
S39	TI (((psychosocial or social) n1 work*)) OR AB (((psychosocial or social) n1 work*))	48,187
S38	TI (((psychoeducat* or psycho educat*) n3 (group or network* or service*))) OR AB (((psychoeducat* or psycho educat*) n3 (group or network* or service*)))	1,967
S37	TI ((peer* n3 (assist* or counsel* or educat* or program* or rehab* or service* or supervis*))) OR AB ((peer* n3 (assist* or counsel* or educat* or program* or rehab* or service* or supervis*)))	7,910
S36	TI ((((lay or peer*) n3 (advis* or consultant or educator* or expert* or facilitator* or instructor* or leader* or mentor* or person* or tutor* or	10,195

For peer review only

	worker*)) or expert patient* or mutual aid)) OR AB ((((lay or peer*) n3 (advis* or consultant or educator* or expert* or facilitator* or instructor* or leader* or mentor* or person* or tutor* or worker*)) or expert patient* or mutual aid))	
S35	TI ((((consumer* or famil* or friend* or lay or mutual* or peer* or social* or spous* or voluntary or volunteer*) n3 (assist* or advice* or advis* or counsel* or educat* or forum* or help* or mentor* or network* or support* or visit*)) or ((consumer* or famil* or peer* or self help or social* or support* or voluntary or volunteer*) n2 group*) or ((consumer* or famil* or friend* or lay or mutual* or peer* or self help or social* or spous* or support* or voluntary or volunteer*) n3 (intervention* or program* or rehab* or therap* or service* or skill* or treat*)) or (((consumer* or famil* or friend* or lay* or peer* or spous* or user* or support* or voluntary or volunteer*) n1 (based or counsel* or deliver* or interact* or led or mediat* or operated or provides or provider* or run*)) or ((consumer* or famil* or friend* or lay* or peer* or relation* or spous* or support*) n3 trust*) or voluntary work*))) OR AB ((((consumer* or famil* or friend* or lay or mutual* or peer* or social* or spous* or voluntary or volunteer*) n3 (assist* or advice* or advis* or counsel* or educat* or forum* or help* or mentor* or network* or support* or visit*)) or ((consumer* or famil* or peer* or self help or social* or support* or voluntary or volunteer*) n2 group*) or ((consumer* or famil* or friend* or lay or mutual* or peer* or self help or social* or spous* or support* or voluntary or volunteer*) n3 (intervention* or program* or rehab* or therap* or service* or skill* or treat*)) or (((consumer* or famil* or friend* or lay* or peer* or spous* or user* or support* or voluntary or volunteer*) n1 (based or counsel* or deliver* or interact* or led or mediat* or operated or provides or provider* or run*)) or ((consumer* or famil* or friend* or lay* or peer* or relation* or spous* or support*) n3 trust*) or voluntary work*)))	413,615
S34	TI ((befriend* or be# friend* or buddy or buddies or ((community or lay or paid or support) n1 (person or worker*)))) OR AB ((befriend* or be# friend* or buddy or buddies or ((community or lay or paid or support) n1 (person or worker*))))	6,926
S33	SU (((self n1 (administer* or assess* or attribut* or care or change or directed or efficacy or help* or guide* or instruct* or manag* or medicat* or monitor* or regulat* or reinforc* or re inforc* or support* or technique* or therap* or train* or treat*)) or selfadminister* or selfassess* or selfattribut* or selfcare or selfchange or selfdirected or selfefficacy or selfhelp* or selfguide* or selfinstruct* or selfmanag* or selfmedicat* or selfmonitor* or selfregulat* or selfreinforc* or self re inforc* or selfsupport* or selftechnique* or selftherap* or selftrain* or selftreat* or (wellness n1 (therap* or train* or treat*)))) OR TI (((self n1 (administer* or assess* or attribut* or care or change or directed or efficacy or help* or guide* or instruct* or manag* or medicat* or monitor* or regulat* or reinforc* or re inforc* or support* or technique* or therap* or train* or treat*)) or selfadminister* or selfassess* or selfattribut* or selfcare or selfchange or selfdirected or selfefficacy or selfhelp* or selfguide* or selfinstruct* or selfmanag* or selfmedicat* or selfmonitor* or selfregulat* or selfreinforc* or self re inforc* or selfsupport* or selftechnique* or selftherap* or selftrain* or selftreat* or (wellness n1 (therap* or train* or treat*)))) OR AB (((self n1 (administer* or assess* or attribut* or care or change or directed or efficacy or help* or guide* or instruct* or manag* or medicat* or monitor* or regulat* or reinforc* or re inforc* or support* or technique* or therap* or train*	163,016

For peer review only

	or treat*)) or selfadminister* or selfassess* or selfattribut* or selfcare or selfchange or selfdirected or selfefficacy or selfhelp* or selfguide* or selfinstruct* or selfmanag* or selfmedicat* or selfmonitor* or selfregulat* or selfreinforc* or self re inforc* or selfsupport* or selftechnique* or selftherap* or selftrain* or selftreat* or (wellness n1 (therap* or train* or treat*))))	
S32	((((((((DE "Friendship") OR (DE "Network Therapy")) OR (DE "Social Networks" OR DE "Online Social Networks")) OR (DE "Peer Relations")) OR (DE "Peers")) OR (DE "Peer Counseling")) OR (DE "Self-Care Skills")) OR (DE "Self-Help Techniques" OR DE "Self-Management")) OR (DE "Social Support")) OR (DE "Support Groups" OR DE "Twelve Step Programs")	120,236
S31	SU psychoeducation OR AB ((psychoed* or psycho ed*)) OR TI ((psychoed* or psycho ed*))	11,472
S30	TI (((child* n2 family traumatic stress intervention) or cftsi)) OR AB (((child* n2 family traumatic stress intervention) or cftsi))	6
S29	(((con*joint or couple* or family or families or husband* or marriage* or marital* or partner* or relations* or spous* or wife or wives* or (child* n5 parent*)) n6 (counsel* or intervention* or program* or support* or therap* or treat*)) or ((couples* or family* or relations*) n1 (based or focused or led)) or ecological therap* or expressed emotion or family dynamics or family relationships)	860,043
S28	((((DE "Couples Therapy") OR (DE "Family Intervention")) OR (DE "Family Therapy" OR DE "Conjoint Therapy" OR DE "Strategic Family Therapy" OR DE "Structural Family Therapy")) OR (DE "Marriage Counseling" OR DE "Conjoint Therapy")) OR (DE "Parent Training")	39,528
S27	TI ((hg therap* or human givens)) OR AB ((hg therap* or human givens))	1,727
S26	TI counsel* OR AB counsel*	102,924
S25	DE "Counseling" OR DE "Community Counseling" OR DE "Cross Cultural Counseling" OR DE "Educational Counseling" OR DE "Genetic Counseling" OR DE "Gerontological Counseling" OR DE "Grief Counseling" OR DE "Group Counseling" OR DE "Marriage Counseling" OR DE "Microcounseling" OR DE "Multicultural Counseling" OR DE "Occupational Guidance" OR DE "Pastoral Counseling" OR DE "Peer Counseling" OR DE "Premarital Counseling" OR DE "Psychotherapeutic Counseling" OR DE "Rehabilitation Counseling" OR DE "School Counseling"	62,561
S24	TI (psychoanal* or psychosomatic*) OR AB (psychoanal* or psychosomatic*)	90,066
S23	TI (((psychodynamic or (dynamic adj (psychotherapy* or therap*)) or incident reduction) or ((brain or transcranial) n2 stimulat*) or rtms)) OR AB (((psychodynamic or (dynamic adj (psychotherapy* or therap*)) or incident reduction) or ((brain or transcranial) n2 stimulat*) or rtms))	38,235

For peer review only

S22	DE "Psychodynamic Psychotherapy"	3,370
S21	TI ((emdr or (eye movement n2 desensiti*))) OR AB ((emdr or (eye movement n2 desensiti*)))	1,872
S20	DE "Eye Movement Desensitization Therapy"	1,437
S19	TI debrief* OR AB debrief*	2,815
S18	DE "Debriefing (Psychological)"	283
S17	TI ((kidnet or mindful* or narrative therap*)) OR AB ((kidnet or mindful* or narrative therap*))	16,869
S16	SU (mindfulness or ((exposure or narrative) n1 therapy))	14,174
S15	TI (((presleep or presleep) n2 imagery)) OR AB (((presleep or presleep) n2 imagery))	0
S14	((imagery n2 (rehears* or re hears*)) or (((lower* or reduc*) n3 (bad dream* or nightmare*)) and (intervention* or program* or therap* or treat*)) or ((intervention* or program* or therap* or treat*) n3 nightmare*))	1,694
S13	TI ((((augmented or virtual) n2 reality) or (virtual n1 (environment or restorative)) or ((exposure or implosive or virtual reality) n2 (intervention* or program* or therap* or train*)))) OR AB ((((augmented or virtual) n2 reality) or (virtual n1 (environment or restorative)) or ((exposure or implosive or virtual reality) n2 (intervention* or program* or therap* or train*))))	13,974
S12	((DE "Exposure Therapy") OR (DE "Narrative Therapy")) OR (DE "Virtual Reality")	10,913
S11	TI ((((compassion or emotion* or emotive*) n1 (based or focused or led)) or emotional processing or ((compassion or emotion* or emotive*) n3 (coach* or intervention* or program* or therap* or treat*)))) OR AB ((((compassion or emotion* or emotive*) n1 (based or focused or led)) or emotional processing or ((compassion or emotion* or emotive*) n3 (coach* or intervention* or program* or therap* or treat*))))	25,414
S10	(DE "Emotion Focused Therapy") OR (DE "Sympathy")	3,110
S9	TI ((((behaviour* or behavior*) n2 cognitiv*) or cbt or ccbt or ((behav* or cognitive*) n3 (intervention* or manag* or program* or restructure* or therap* or treat*)) or (stress inoculation n2 (intervention* or program* or therap* or train* or treat*)) or (behav* n2 activat*) or ((trauma n1 (based or focused or led)) or exposure based or prolonged exposure))) OR AB ((((behaviour* or behavior*) n2 cognitiv*) or cbt or ccbt or ((behav* or cognitive*) n3 (intervention* or manag* or program* or restructure* or therap* or treat*)) or (stress inoculation n2 (intervention* or program* or therap* or train* or treat*))	154,978

For peer review only

	or (behav* n2 activat*) or ((trauma n1 (based or focused or led)) or exposure based or prolonged exposure)))	
S8	(DE "Behavior Therapy" OR DE "Aversion Therapy" OR DE "Conversion Therapy" OR DE "Dialectical Behavior Therapy" OR DE "Exposure Therapy" OR DE "Implosive Therapy" OR DE "Reciprocal Inhibition Therapy" OR DE "Response Cost" OR DE "Systematic Desensitization Therapy") OR (DE "Cognitive Behavior Therapy" OR DE "Acceptance and Commitment Therapy" OR DE "Cognitive Processing Therapy" OR DE "Prolonged Exposure Therapy")	49,013
S7	TI ((((psycholog* or psycho social* or psychosocial*) n3 (intervention* or program* or therap* or treat*)) or psychotherap* or psycho therap* or talk* therap* or therapeutic technique* or therapist* or third wave or time limited)) OR AB ((((psycholog* or psycho social* or psychosocial*) n3 (intervention* or program* or therap* or treat*)) or psychotherap* or psycho therap* or talk* therap* or therapeutic technique* or therapist* or third wave or time limited)) OR SU ((((psycholog* or psycho social* or psychosocial*) n3 (intervention* or program* or therap* or treat*)) or psychotherap* or psycho therap* or talk* therap* or therapeutic technique* or therapist* or third wave or time limited))	281,242
S6	DE "Psychotherapy"	67,719
S5	S1 OR S2 OR S3 OR S4	140,653
S4	TI ((railway spine or (rape n2 trauma*) or reexperienc* or re experienc* or torture syndrome or traumatic neuros* or traumatic stress)) OR AB ((railway spine or (rape n2 trauma*) or reexperienc* or re experienc* or torture syndrome or traumatic neuros* or traumatic stress))	19,098
S3	TI ((trauma* and (avoidance or grief or horror or death* or nightmare* or night mare* or emotion*))) OR AB ((trauma* and (avoidance or grief or horror or death* or nightmare* or night mare* or emotion*)))	23,709
S2	TI ((posttraumatic* or post traumatic* or stress disorder* or acute stress or ptsd or asd or desnos or (combat neuros* or combat syndrome or concentration camp syndrome or extreme stress or flashback* or flash back* or hypervigilan* or hypervigilen* or psych* stress or psych* trauma* or psycho?trauma* or psychotrauma*) or (posttrauma* or traumagenic* or traumatic stress*))) OR AB ((posttraumatic* or post traumatic* or stress disorder* or acute stress or ptsd or asd or desnos or (combat neuros* or combat syndrome or concentration camp syndrome or extreme stress or flashback* or flash back* or hypervigilan* or hypervigilen* or psych* stress or psych* trauma* or psycho?trauma* or psychotrauma*) or (posttrauma* or traumagenic* or traumatic stress*)))	104,519
S1	((((((((DE "Posttraumatic Stress Disorder" OR DE "Complex PTSD" OR DE "DESNOS") OR (DE "Acute Stress Disorder")) OR (DE "Combat Experience")) OR (DE "Emotional Trauma")) OR (DE "Post-Traumatic Stress")) OR (DE "Traumatic Neurosis")) OR (DE "Trauma")) OR (DE "Psychological Stress")) OR (DE "Chronic Stress")	70,398

For peer review only

CINAHL

#	Query	Results
S54	S51 NOT S49	11,937
S53	S51 NOT S49 Limiters - Published Date: 20180101-20191231	2,506
S52	S51 NOT S49	11,937
S51	S6 AND S50	12,028
S50	S40 OR S48	670,268
S49	MH ANIMALS NOT MH HUMANS	79,628
S48	s41 or s42 or s43 or s44 or s45 or s46 or s47	505,183
S47	TI (placebo* or random*) OR AB (placebo* or random*)	318,823
S46	TI (single blind* or double blind* or treble blind* or mask* or dummy* or singleblind* or doubleblind* or trebleblind* or tripleblind*) OR AB (single blind* or double blind* or treble blind* or mask* or dummy* or singleblind* or doubleblind* or trebleblind* or tripleblind*)	54,242
S45	TI (crossover or cross over) OR AB (crossover or cross over)	17,078
S44	TI clinical n2 trial* OR AB clinical n2 trial*	95,858
S43	(MH "Crossover Design") OR (MH "Placebos") OR (MH "Random Assignment")	78,307
S42	MW double blind* or single blind* or triple blind*	55,488
S41	(MH "Clinical Trials+")	268,307
S40	s7 or s8 or s9 or s10 or s11 or s12 or s13 or s14 or s15 or s16 or s17 or s18 or s19 or s20 or s21 or s22 or s23 or s29 or s30 or s31 or s34 or s35 or s36 or s37 or s38 or s39	236,549
S39	TI ((analy* n5 review*) or (evidence* n5 review*) or (methodol* n5 review*) or (quantativ* n5 review*) or (systematic* n5 review*)) OR AB ((analy* n5 review*) or (evidence* n5 review*) or (methodol* n5 review*) or (quantativ* n5 review*) or (systematic* n5 review*))	121,320

For peer review only

S38	TI ((pool* n2 results) or (combined n2 results) or (combining n2 results)) OR AB ((pool* n2 results) or (combined n2 results) or (combining n2 results))	5,060
S37	TI ((pool* n2 studies) or (combined n2 studies) or (combining n2 studies)) OR AB ((pool* n2 studies) or (combined n2 studies) or (combining n2 studies))	5,591
S36	TI ((pool* n2 trials) or (combined n2 trials) or (combining n2 trials)) OR AB ((pool* n2 trials) or (combined n2 trials) or (combining n2 trials))	1,344
S35	TI ((pool* n2 data) or (combined n2 data) or (combining n2 data)) OR AB ((pool* n2 data) or (combined n2 data) or (combining n2 data))	8,792
S34	s32 and s33	3,735
S33	TI review* AND PT review*	24,679
S32	TI analy* or assessment* or evidence* or methodol* or quantativ* or qualitativ* or systematic*	393,433
S31	TI systematic* n5 search* OR AB systematic* n5 search*	14,939
S30	TI systematic* n5 review* OR AB systematic* n5 review*	88,888
S29	(s24 or s25 or s26) and (s27 or s28)	81,701
S28	TI systematic* OR AB systematic*	133,890
S27	TX review* OR MW review* OR PT review*	4,746,766
S26	(MH "Cochrane Library")	23,041
S25	TI (bids or cochrane or embase or “index medicus” or “isi citation” or medline or psyclit or psychlit or scisearch or “science citation” or (web n2 science)) OR AB (bids or cochrane or embase or “index medicus” or “isi citation” or medline or psyclit or psychlit or scisearch or “science citation” or (web n2 science))	65,455
S24	TI (“electronic database*” or “bibliographic database*” or “computeri?ed database*” or “online database*”) OR AB (“electronic database*” or “bibliographic database*” or “computeri?ed database*” or “online database*”)	14,423
S23	(MH "Literature Review")	7,803
S22	PT (systematic*) or PT(meta*)	86,982

For peer review only

S21	TI (“fixed effect*” or “random effect*”) OR AB (“fixed effect*” or “random effect*”)	17,363
S20	TI (“mantel haenszel” or peto or dersimonian or “der simonian”) OR AB (“mantel haenszel” or peto or dersimonian or “der simonian”)	1,726
S19	TI (handsearch* or "hand search*" or "manual search*") OR AB (handsearch* or "hand search*" or "manual search*")	5,430
S18	AB "data extraction" or "data synthesis"	8,188
S17	AB "selection criteria"	4,257
S16	AB "relevant journals"	433
S15	AB "published studies"	5,562
S14	AB bibliograph*	6,982
S13	TI "reference list*"	21
S12	AB "reference list*"	7,455
S11	TI (“research review*” or “research integration”) OR AB (“research review*” or “research integration”)	1,467
S10	(MH "Meta Analysis")	40,851
S9	TI (metaanal* or “meta anal*” or metasynthes* or “meta synethes*”) OR AB (metaanal* or “meta anal*” or metasynthes* or “meta synethes*”)	61,476
S8	(MH "Systematic Review")	74,342
S7	(MH "Literature Searching+")	7,587
S6	s1 or s2 or s3 or s4 or s5	91,542
S5	TI ((posttraumatic* or "post traumatic*" or "stress disorder*" or "acute stress" or ptsd or asd or desnos or ("combat neuros*" or "combat syndrome" or "concentration camp syndrome" or "extreme stress" or flashback* or "flash back*" or hypervigilan* or hypervigilen* or "psych* stress" or "psych* trauma*" or psychotrauma* or psychotrauma*) or (posttrauma* or traumagenic* or "traumatic stress*"))) or AB ((posttraumatic* or "post traumatic*" or "stress disorder*" or "acute stress" or ptsd or asd or desnos or ("combat neuros*" or "combat syndrome" or "concentration camp syndrome" or "extreme stress" or flashback* or "flash back*" or hypervigilan* or hypervigilen* or "psych* stress" or "psych* trauma*" or psychotrauma* or psychotrauma*) or (posttrauma* or traumagenic* or "traumatic stress*")))	35,785

For peer review only

S4	TI ((trauma* and (avoidance or grief or horror or death* or nightmare* or "night mare*" or emotion*))) or AB ((trauma* and (avoidance or grief or horror or death* or nightmare* or "night mare*" or emotion*)))	11,611
S3	TI (("railway spine" or (rape near/2 trauma*) or reexperienc* or "re experienc*" or "torture syndrome" or "traumatic neuros*" or "traumatic stress")) or AB (("railway spine" or (rape near/2 trauma*) or reexperienc* or "re experienc*" or "torture syndrome" or "traumatic neuros*" or "traumatic stress"))	6,744
S2	(MH "stress, psychological")	44,491
S1	(MH "stress disorders, post-traumatic")	20,118

Cochrane Library

Search Name: anke

Date Run: 13/11/2019 09:24:52

Comment:

ID Search Hits

#1 MeSH descriptor: [Stress Disorders, Traumatic] explode all trees 2394

#2 MeSH descriptor: [Combat Disorders] explode all trees 125

#3 MeSH descriptor: [Psychological Trauma] explode all trees 57

#4 MeSH descriptor: [Stress Disorders, Post-Traumatic] explode all trees 2275

#5 MeSH descriptor: [Stress Disorders, Traumatic, Acute] explode all trees 43

#6 MeSH descriptor: [Stress, Psychological] explode all trees 5703

#7 ("railway spine" or (rape near/2 trauma*) or reexperienc* or "re experienc*" or "torture syndrome" or "traumatic neuros*" or "traumatic stress"):ti 716

#8 ("railway spine" or (rape near/2 trauma*) or reexperienc* or "re experienc*" or "torture syndrome" or "traumatic neuros*" or "traumatic stress"):ab 1672

#9 (trauma* and (avoidance or grief or horror or death* or nightmare* or "night mare*" or

emotion*)):ti 162

#10 (trauma* and (avoidance or grief or horror or death* or nightmare* or "night mare*" or

emotion*)):ab 2642

#11 (posttraumatic* or "post traumatic*" or "stress disorder*" or "acute stress" or ptsd or asd or desnos or ("combat neuros*" or "combat syndrome" or "concentration camp syndrome" or "extreme stress" or flashback* or "flash back*" or hypervigilan* or hypervigilen* or "psych* stress" or "psych*

For peer review only

trauma*" or psychotrauma* or psychotrauma*) or (posttrauma* or traumagenic* or "traumatic stress*")):ti 4046

#12 (posttraumatic* or "post traumatic*" or "stress disorder*" or "acute stress" or ptsd or asd or desnos or ("combat neuros*" or "combat syndrome" or "concentration camp syndrome" or "extreme stress" or flashback* or "flash back*" or hypervigilan* or hypervigilen* or "psych* stress" or "psych* trauma*" or psychotrauma* or psychotrauma*) or (posttrauma* or traumagenic* or "traumatic stress*")):ab 7920

#13 #1 or #2 or #3 or #4 or #5 or #6 or #7 or #8 or #9 or #10 or #11 or #12 with Publication Year from 2018 to 2019, with Cochrane Library publication date Between Jan 2018 and Dec 2019, in Trials 2413

For peer review only
